# Supplementary material for: Randomised controlled pilot trial of an exercise plus behaviour change intervention in people with multiple sclerosis: the Step it Up study
Source: BMJ Open. 2017 Oct 12;7(10):e016336. doi: 10.1136/bmjopen-2017-016336 (PMC5654468; doi:10.1136/bmjopen-2017-016336)
Supplement: Supplementary file 3 [file bmjopen-2017-016336supp003.pdf]

Your Step it Up exercise Log book

Please use this log book to record **BOTH** the strengthening exercises and the walking exercise that you do throughout this programme. This log book is to be used **both during your exercise classes** with your physiotherapist and **while you are doing your exercises at home**. The reason we ask you to complete this log is so you can keep a reliable record of progress that you are making with the Step it Up programme. It is also important for the research team at UL to track your progress with the programme.

Each week you should fill the relevant table. As you can see, in the last 2 columns of each of the tables- we have asked you to rate your “BORG score” and your “enjoyment score”- at the end of this document you will see instructions on how to complete these ratings.

If you have any questions regarding filling this log book out please do not hesitate to contact Dr. Susan Coote (susan.coote@ul.ie) or the physiotherapist who is delivering your programme. When the 10-week exercise class is finished, please give your log book to the physiotherapist who completes your follow-up assessment (Dr. Sara Hayes). You will be given a date for this assessment closer to the time.

© University of Limerick/University of Illinois, Urbana-Champaign 2014. This information is being provided solely for the purpose of delivering this intervention as part of the HRB funded Step it Up Trial and should not to be reproduced without permission or used for any other purpose.

Week 1

Date	Strengthening exercises										BORG score (6-20)	Enjoyment score (1-7)
Class	Knee bend	Squat	Shoulder bend-forward	Shoulder bend-sideways	Elbow bend	Lunge	Hip bend-backward	Hip bend-sideways	Heel raises-standing	Ankle bend-sitting		
	Reps	Reps	Reps	Reps	Reps	Reps	Reps	Reps	Reps	Reps		
	Sets	Sets	Sets	Sets	Sets	Sets	Sets	Sets	Sets	Sets		
	Colour	Colour	Colour	Colour	Colour	Colour	Colour	Colour	Colour	Colour		
Home	Knee bend	Squat	Shoulder bend-forward	Shoulder bends-sideways	Elbow bend	Lunge	Hip bend-backward	Hip bends-sideways	Heel raises-standing	Ankle bend-sitting		
	Reps	Reps	Reps	Reps	Reps	Reps	Reps	Reps	Reps	Reps		
	Sets	Sets	Sets	Sets	Sets	Sets	Sets	Sets	Sets	Sets		
	Colour	Colour	Colour	Colour	Colour	Colour	Colour	Colour	Colour	Colour		
	Walking exercise											
Home	Time spent walking (minutes):				Number of steps:							
Home	Time spent walking (minutes):				Number of steps:							

Week 2

**Date**	**Strengthening exercises**										**BORG score (6-20)**	**Enjoyment score (1-7)**
**Class**	**Knee bend**	**Squat**	**Shoulder bend-forward**	**Shoulder bends-sideways**	**Elbow bend**	**Lunge**	**Hip bend-backward**	**Hip bend-sideways**	**Heel raises-standing**	**Ankle bend-sitting**		
	Reps	Reps	Reps	Reps	Reps	Reps	Reps	Reps	Reps	Reps		
	Sets	Sets	Sets	Sets	Sets	Sets	Sets	Sets	Sets	Sets		
	Colour	Colour	Colour	Colour	Colour	Colour	Colour	Colour	Colour	Colour		
**Home**	**Knee bend**	**Squat**	**Shoulder bend-forward**	**Shoulder bends-sideways**	**Elbow bend**	**Lunge**	**Hip bend-backward**	**Hip bends-sideways**	**Heel raises-standing**	**Ankle bend-sitting**		
	Reps	Reps	Reps	Reps	Reps	Reps	Reps	Reps	Reps	Reps		
	Sets	Sets	Sets	Sets	Sets	Sets	Sets	Sets	Sets	Sets		
	Colour	Colour	Colour	Colour	Colour	Colour	Colour	Colour	Colour	Colour		
	**Walking exercise**											
**Home**	**Time spent walking (minutes):**					**Number of steps:**						
**Home**	**Time spent walking (minutes):**					**Number of steps:**						

Week 3

Date	Strengthening exercises										BORG score (6-20)	Enjoyment score (1-7)
**Class**	**Knee bend**	**Squat**	**Shoulder bend-forward**	**Shoulder bends-sideways**	**Elbow bend**	**Lunge**	**Hip bend-backward**	**Hip bend-sideways**	**Heel raises-standing**	**Ankle bend-sitting**		
	Reps	Reps	Reps	Reps	Reps	Reps	Reps	Reps	Reps	Reps		
	Sets	Sets	Sets	Sets	Sets	Sets	Sets	Sets	Sets	Sets		
	Colour	Colour	Colour	Colour	Colour	Colour	Colour	Colour	Colour	Colour		
**Home**	**Knee bend**	**Squat**	**Shoulder bend-forward**	**Shoulder bends-sideways**	**Elbow bend**	**Lunge**	**Hip bend-backward**	**Hip bend-sideways**	**Heel raises-standing**	**Ankle bend-sitting**		
	Reps	Reps	Reps	Reps	Reps	Reps	Reps	Reps	Reps	Reps		
	Sets	Sets	Sets	Sets	Sets	Sets	Sets	Sets	Sets	Sets		
	Colour	Colour	Colour	Colour	Colour	Colour	Colour	Colour	Colour	Colour		
	**Walking exercise**											
**Home**	**Time spent walking (minutes):**				**Number of steps:**							
**Home**	**Time spent walking (minutes):**				**Number of steps:**							

Week 4

Date	Strengthening exercises										BORG score (6-20)	Enjoyment score (1-7)
Home	**Knee bend**	**Squat**	**Shoulder bend-forward**	**Shoulder bends-sideways**	**Elbow bend**	**Lunge**	**Hip bend-backward**	**Hip bends-sideways**	**Heel raises-standing**	**Ankle bend-sitting**		
	Reps	Reps	Reps	Reps	Reps	Reps	Reps	Reps	Reps	Reps		
	Sets	Sets	Sets	Sets	Sets	Sets	Sets	Sets	Sets	Sets		
	Colour	Colour	Colour	Colour	Colour	Colour	Colour	Colour	Colour	Colour		
Home	**Knee bend**	**Squat**	**Shoulder bend-forward**	**Shoulder bends-sideways**	**Elbow bend**	**Lunge**	**Hip bend-backward**	**Hip bends-sideways**	**Heel raises-standing**	**Ankle bend-sitting**		
	Reps	Reps	Reps	Reps	Reps	Reps	Reps	Reps	Reps	Reps		
	Sets	Sets	Sets	Sets	Sets	Sets	Sets	Sets	Sets	Sets		
	Colour	Colour	Colour	Colour	Colour	Colour	Colour	Colour	Colour	Colour		
	**Walking exercise**											
Home	**Time spent walking (minutes):**				**Number of steps:**							
Home	**Time spent walking (minutes):**				**Number of steps:**							

Week 5

Date	Strengthening exercises										BORG score (6-20)	Enjoyment score (1-7)
**Class**	**Knee bend**	**Squat**	**Shoulder bend-forward**	**Shoulder bends-sideways**	**Elbow bend**	**Lunge**	**Hip bend-backward**	**Hip bends-sideways**	**Heel raises-standing**	**Ankle bend-sitting**		
	Reps	Reps	Reps	Reps	Reps	Reps	Reps	Reps	Reps	Reps		
	Sets	Sets	Sets	Sets	Sets	Sets	Sets	Sets	Sets	Sets		
	Colour	Colour	Colour	Colour	Colour	Colour	Colour	Colour	Colour	Colour		
**Home**	**Knee bend**	**Squat**	**Shoulder bend-forward**	**Shoulder bends-sideways**	**Elbow bend**	**Lunge**	**Hip bend-backward**	**Hip bend-sideways**	**Heel raises-standing**	**Ankle bend-sitting**		
	Reps	Reps	Reps	Reps	Reps	Reps	Reps	Reps	Reps	Reps		
	Sets	Sets	Sets	Sets	Sets	Sets	Sets	Sets	Sets	Sets		
	Colour	Colour	Colour	Colour	Colour	Colour	Colour	Colour	Colour	Colour		
	**Walking exercise**											
**Home**	**Time spent walking (minutes):**				**Number of steps:**							
**Home**	**Time spent walking (minutes):**				**Number of steps:**							

Week 6

Date	Strengthening exercises										BORG score (6-20)	Enjoyment score (1-7)
**Home**	**Knee bend**	**Squat**	**Shoulder bend-forward**	**Shoulder bends-sideways**	**Elbow bend**	**Lunge**	**Hip bend-backward**	**Hip bend-sideways**	**Heel raises-standing**	**Ankle bend-sitting**		
	Reps	Reps	Reps	Reps	Reps	Reps	Reps	Reps	Reps	Reps		
	Sets	Sets	Sets	Sets	Sets	Sets	Sets	Sets	Sets	Sets		
	Colour	Colour	Colour	Colour	Colour	Colour	Colour	Colour	Colour	Colour		
**Home**	**Knee bend**	**Squat**	**Shoulder bend-forward**	**Shoulder bends-sideways**	**Elbow bend**	**Lunge**	**Hip bend-backward**	**Hip bend-sideways**	**Heel raises-standing**	**Ankle bend-sitting**		
	Reps	Reps	Reps	Reps	Reps	Reps	Reps	Reps	Reps	Reps		
	Sets	Sets	Sets	Sets	Sets	Sets	Sets	Sets	Sets	Sets		
	Colour	Colour	Colour	Colour	Colour	Colour	Colour	Colour	Colour	Colour		
	**Walking exercise**											
**Home**	**Time spent walking (minutes):**				**Number of steps:**							
**Home**	**Time spent walking (minutes):**				**Number of steps:**							

Week 7

Date	Strengthening exercises										BORG score (6-20)	Enjoyment score (1-7)
**Home**	**Knee bend**	**Squat**	**Shoulder bend-forward**	**Shoulder bends-sideways**	**Elbow bend**	**Lunge**	**Hip bend-backward**	**Hip bend-sideways**	**Heel raises-standing**	**Ankle bend-sitting**		
	Reps	Reps	Reps	Reps	Reps	Reps	Reps	Reps	Reps	Reps		
	Sets	Sets	Sets	Sets	Sets	Sets	Sets	Sets	Sets	Sets		
	Colour	Colour	Colour	Colour	Colour	Colour	Colour	Colour	Colour	Colour		
**Home**	**Knee bend**	**Squat**	**Shoulder bend-forward**	**Shoulder bends-sideways**	**Elbow bend**	**Lunge**	**Hip bend-backward**	**Hip bend-sideways**	**Heel raises-standing**	**Ankle bend-sitting**		
	Reps	Reps	Reps	Reps	Reps	Reps	Reps	Reps	Reps	Reps		
	Sets	Sets	Sets	Sets	Sets	Sets	Sets	Sets	Sets	Sets		
	Colour	Colour	Colour	Colour	Colour	Colour	Colour	Colour	Colour	Colour		
	**Walking exercise**											
**Home**	**Time spent walking (minutes):**				**Number of steps:**							
**Home**	**Time spent walking (minutes):**				**Number of steps:**							

Week 8

**Date**	**Strengthening exercises**										**BORG score (6-20)**	**Enjoyment score (1-7)**
**Class**	**Knee bend**	**Squat**	**Shoulder bend-forward**	**Shoulder bends-sideways**	**Elbow bend**	**Lunge**	**Hip bend-backward**	**Hip bend-sideways**	**Heel raises-standing**	**Ankle bend-sitting**		
	Reps	Reps	Reps	Reps	Reps	Reps	Reps	Reps	Reps	Reps		
	Sets	Sets	Sets	Sets	Sets	Sets	Sets	Sets	Sets	Sets		
	Colour	Colour	Colour	Colour	Colour	Colour	Colour	Colour	Colour	Colour		
**Home**	**Knee bend**	**Squat**	**Shoulder bend-forward**	**Shoulder bends-sideways**	**Elbow bend**	**Lunge**	**Hip bend-backward**	**Hip bend-sideways**	**Heel raises-standing**	**Ankle bend-sitting**		
	Reps	Reps	Reps	Reps	Reps	Reps	Reps	Reps	Reps	Reps		
	Sets	Sets	Sets	Sets	Sets	Sets	Sets	Sets	Sets	Sets		
	Colour	Colour	Colour	Colour	Colour	Colour	Colour	Colour	Colour	Colour		
	**Walking exercise**											
**Home**	**Time spent walking (minutes):**				**Number of steps:**							
**Home**	**Time spent walking (minutes):**				**Number of steps:**							

Week 9

Date	Strengthening exercises										BORG score (6-20)	Enjoyment score (1-7)
Home	Knee bend	Squat	Shoulder bend-forward	Shoulder bends-sideways	Elbow bend	Lunge	Hip bend-backward	Hip bend-sideways	Heel raises-standing	Ankle bend-sitting		
	Reps	Reps	Reps	Reps	Reps	Reps	Reps	Reps	Reps	Reps		
	Sets	Sets	Sets	Sets	Sets	Sets	Sets	Sets	Sets	Sets		
	Colour	Colour	Colour	Colour	Colour	Colour	Colour	Colour	Colour	Colour		
Home	Knee bend	Squat	Shoulder bend-forward	Shoulder bends-sideways	Elbow bend	Lunge	Hip bend-backward	Hip bend-sideways	Heel raises-standing	Ankle bend-sitting		
	Reps	Reps	Reps	Reps	Reps	Reps	Reps	Reps	Reps	Reps		
	Sets	Sets	Sets	Sets	Sets	Sets	Sets	Sets	Sets	Sets		
	Colour	Colour	Colour	Colour	Colour	Colour	Colour	Colour	Colour	Colour		
	**Walking exercise**											
**Home**	**Time spent walking (minutes):**					**Number of steps:**						
**Home**	**Time spent walking (minutes):**					**Number of steps:**						

Week 10

Date	Strengthening exercises										BORG score (6-20)	Enjoyment score (1-7)
Class	**Knee bend**	**Squat**	**Shoulder bend-forward**	**Shoulder bends-sideways**	**Elbow bend**	**Lunge**	**Hip bend-backward**	**Hip bend-sideways**	**Heel raises-standing**	**Ankle bend-sitting**		
	Reps	Reps	Reps	Reps	Reps	Reps	Reps	Reps	Reps	Reps		
	Sets	Sets	Sets	Sets	Sets	Sets	Sets	Sets	Sets	Sets		
	Colour	Colour	Colour	Colour	Colour	Colour	Colour	Colour	Colour	Colour		
Home	**Knee bend**	**Squat**	**Shoulder bend-forward**	**Shoulder bends-sideways**	**Elbow bend**	**Lunge**	**Hip bend-backward**	**Hip bend-sideways**	**Heel raises-standing**	**Ankle bend-sitting**		
	Reps	Reps	Reps	Reps	Reps	Reps	Reps	Reps	Reps	Reps		
	Sets	Sets	Sets	Sets	Sets	Sets	Sets	Sets	Sets	Sets		
	Colour	Colour	Colour	Colour	Colour	Colour	Colour	Colour	Colour	Colour		
	**Walking exercise**											
**Home**	**Time spent walking (minutes):**				**Number of steps:**							
**Home**	**Time spent walking (minutes):**				**Number of steps:**							

***BORG scoring instructions**

While doing physical activity, we want you to rate your perception of exertion. This feeling should reflect how heavy and strenuous the exercise feels to you, Combining all sensations and feelings of physical stress, effort, and fatigue. Do not concern yourself with any one factor such as leg pain or shortness of breath, but try to focus on your total feeling of exertion.

Look at the rating scale below while you are engaging in an activity; it ranges from 6 to 20, where 6 means "no exertion at all" and 20 means "maximal exertion." Choose the number from below that best describes your level of exertion. This will give you a good idea of the intensity level of your activity, and you can use this information to speed up or slow down your movements to reach your desired range.

Try to appraise your feeling of exertion as honestly as possible, without thinking about what the actual physical load is. Your own feeling of effort and exertion is important, not how it compares to other people's. Look at the scales and the expressions and then give a number.

---

6 No exertion at all

7 Extremely light

8

9 Very light - (easy walking slowly at a comfortable pace)

10

11 Light

12

13 Somewhat hard (It is quite an effort; you feel tired but can continue)

14

15 Hard (heavy)

16

17 Very hard (very strenuous, and you are very fatigued)

18

19 Extremely hard (You cannot continue for long at this pace)

20 Maximal exertion

© University of Limerick/University of Illinois, Urbana-Champaign 2014. This information is being provided solely for the purpose of delivering this intervention as part of the HRB funded Step it Trial and should not to be reproduced without permission or used for any other purpose.

** **Your Enjoyment Scale Score**

Enjoyment Scale<br>“How much did you enjoy your exercise session today?”						
**1**<br>**not at all**	**2**	**3**	**4**<br>**somewhat**	**5**	**6**	**7**<br>**very much**

© University of Limerick/University of Illinois, Urbana-Champaign 2014. This information is being provided solely for the purpose of delivering this intervention as part of the HRB funded Step it Trial and should not to be reproduced without permission or used for any other purpose.
