# Supplementary material for: Randomised controlled pilot trial of an exercise plus behaviour change intervention in people with multiple sclerosis: the Step it Up study
Source: BMJ Open. 2017 Oct 12;7(10):e016336. doi: 10.1136/bmjopen-2017-016336 (PMC5654468; doi:10.1136/bmjopen-2017-016336)

Knee bend

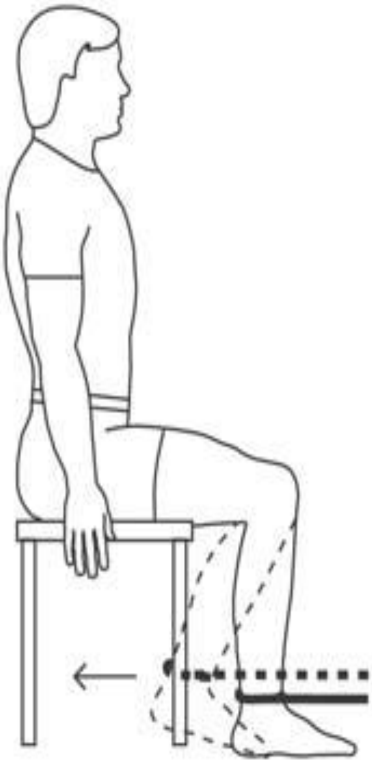

Squat

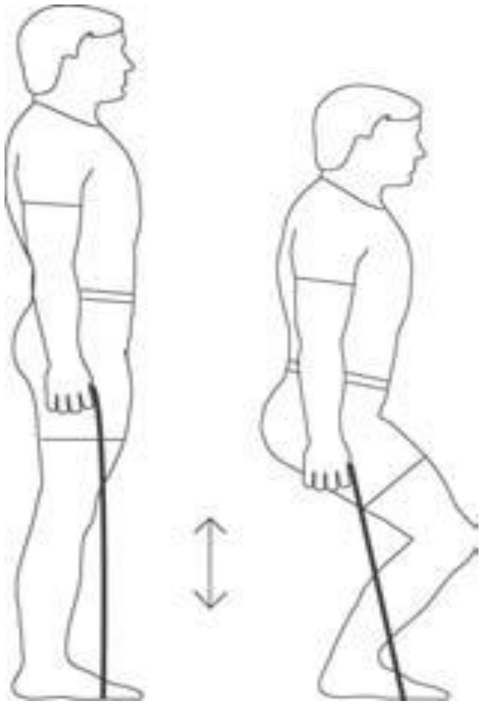

Shoulder bend- forward

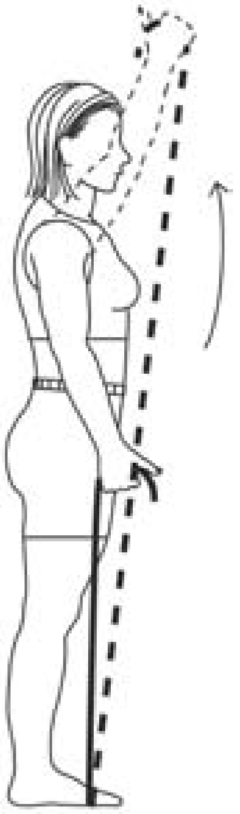

Shoulder bend-sideways

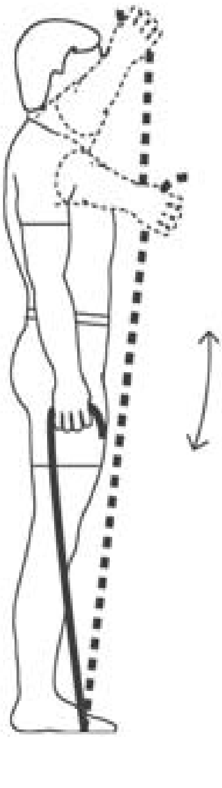

Elbow bend

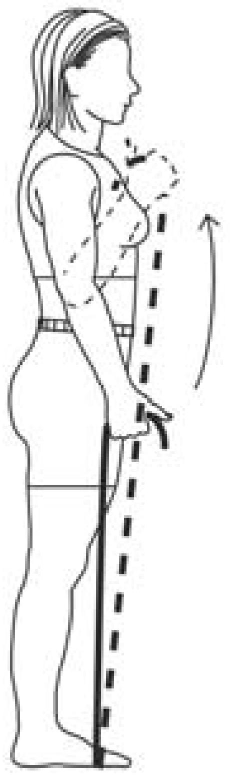

Lunge

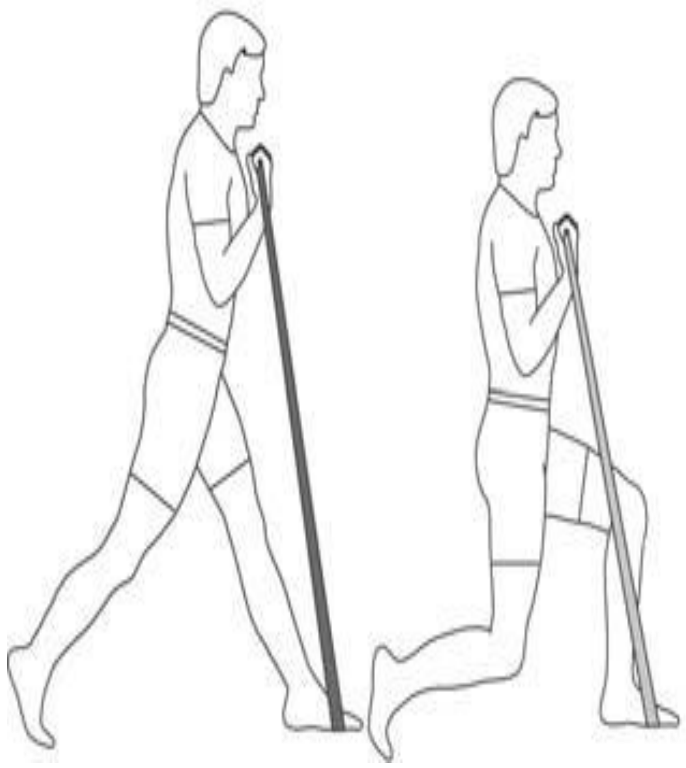

Hip bend- backward

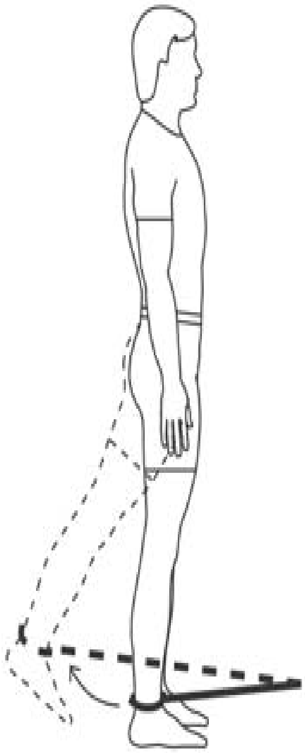

Hip bend- sideways

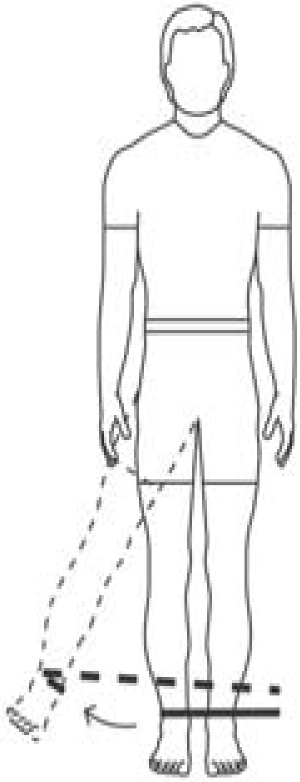

Heel raises- standing

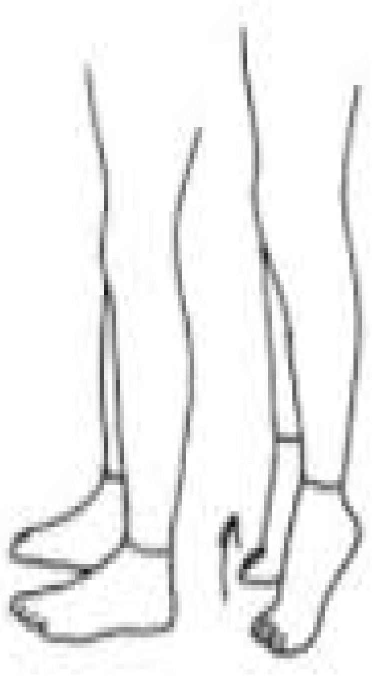

Ankle bend- sitting

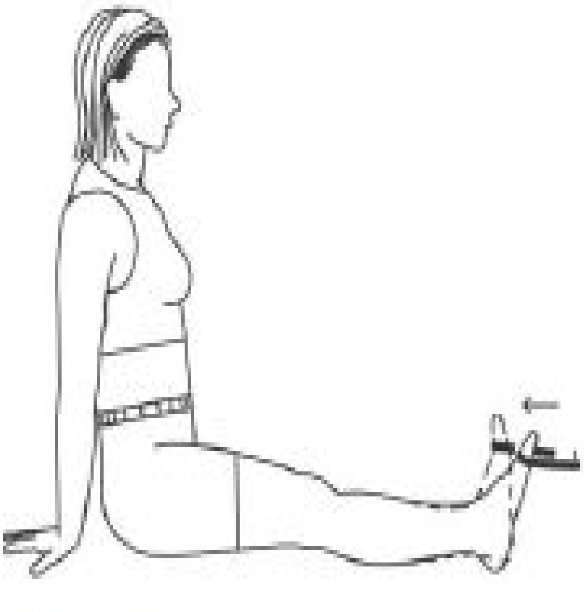

Supplement: Supplementary file 1 [file bmjopen-2017-016336supp001.pdf]
