# Supplementary material for: Randomised controlled pilot trial of an exercise plus behaviour change intervention in people with multiple sclerosis: the Step it Up study
Source: BMJ Open. 2017 Oct 12;7(10):e016336. doi: 10.1136/bmjopen-2017-016336 (PMC5654468; doi:10.1136/bmjopen-2017-016336)
Supplement: Supplementary file 2 [file bmjopen-2017-016336supp002.pdf]

TIDieR Template for Intervention Description and Replication

The TIDieR (Template for Intervention Description and Replication) Checklist*:

Information to include when describing an intervention and the location of the information

Item number	Item	Where located **	
		Primary paper (page or appendix number)	Other † (details)
	**BRIEF NAME**		
**1.**	Provide the name or a phrase that describes the intervention.	1	Title; Exercise plus behaviour change intervention
	**WHY**		
**2.**	Describe any rationale, theory, or goal of the elements essential to the intervention.	5,6	We designed a randomised controlled pilot trial called 'Step it Up' that combined a group exercise programme with a theory-based education component for augmenting the effect of exercise on walking outcomes and sustaining these changes over time. We compared SCT based education to attention control education on topics unrelated to exercise. SCT was used to develop the content of the educational element as it has been widely investigated and associated with PA behaviour in people with MS
	**WHAT**		
**3.**	Materials: Describe any physical or informational materials used in the intervention, including those provided to participants or used in intervention delivery or in training of intervention providers. Provide information on where the	9 Protocol paper page 3, 4 https://bmcneu	The exercise log book and exercise pictures are available as an online appendix

	materials can be accessed (e.g. online appendix, URL).	rol.biomedcentral.com/articles/10.1186/s12883-014-0241-9	
**4.**	Procedures: Describe each of the procedures, activities, and/or processes used in the intervention, including any enabling or support activities.	9<br>Protocol paper page 3	Over the 10-week programme participants attended the group exercise class on six occasions, supplemented with a telephone coaching call in the weeks without classes (intervention weeks 4, 6, 7 and 9). After each of the group exercise classes both groups received an education session and the content is described in the protocol paper
	**WHO PROVIDED**		
**5.**	For each category of intervention provider (e.g. psychologist, nursing assistant), describe their expertise, background and any specific training given.	10	The physiotherapists who delivered the intervention or control group sessions were provided with a one-day training course on the delivery of the intervention for their group.
	**HOW**		
**6.**	Describe the modes of delivery (e.g. face-to-face or by some other mechanism, such as internet or telephone) of the intervention and whether it was provided individually or in a group.	9	There were 44 sessions over 10 weeks; 6 group strengthening classes followed by education, 14 home strengthening classes, 20 home walking sessions and 4 telephone calls
	**WHERE**		
**7.**	Describe the type(s) of location(s) where the intervention occurred, including any necessary infrastructure or relevant features.	9	Recruitment and interventions took place in Cork Galway and Limerick Ireland. All classes happened at community venues, the other sessions were home based.
	**WHEN and HOW MUCH**		
**8.**	Describe the number of times the intervention was delivered	See 6 above	The target walking exercise intensity

	and over what period of time including the number of sessions, their schedule, and their duration, intensity or dose.	Protocol paper page 3 describes intensity of waling and strengthening sessions.	for both groups in the current study was at a rate of 100 steps per minute. Participants started with 10 minutes of walking twice weekly at a rate of 100 steps/minute and increased incrementally in 5 minute intervals over 5 weeks wherein they aimed to reach the guideline of30 minutes twice weekly<br>The intensity and duration of the strengthening component of the intervention was progressed by increasing the number of repetitions and sets and changing the resistance of the elastic resistance band used for each strengthening exercise. Participants started with one set of 10–15 repetitions and gradually increased the number of sets, repetitions and level of resistance until they meet the target of two sets of each exercise twice weekly with sufficient resistance that they are failing on the 12th repetition.
	**TAILORING**		
**9.**	If the intervention was planned to be personalised, titrated or adapted, then describe what, why, when, and how.	Protocol paper page 3	Intensity was personalised based on each participants ability/performance of resistance and aerobic exercise Progression through the programme was based on individual performance in the previous session.
	**MODIFICATIONS**		
**10.**[‡]	If the intervention was modified during the course of the study, describe the changes (what, why, when, and how).	13, 14	Not all participants met the guideline target by week 6. The proportion of participants in each group reaching the guideline and reasons for not reaching guideline are described in the results

	**HOW WELL**		
**11.**	Planned: If intervention adherence or fidelity was assessed, describe how and by whom, and if any strategies were used to maintain or improve fidelity, describe them.	12, Protocol paper page 4	Exercise logs, video or/audio recording of sessions and independent evaluation of those recorded sessions were utilised to evaluate fidelity
**12.‡**	Actual: If intervention adherence or fidelity was assessed, describe the extent to which the intervention was delivered as planned.	13,14	Adherence to the programme evaluated using the exercise logs<br>Fidelity was assessed by an independent person using the video/audio recordings

** **Authors** - use N/A if an item is not applicable for the intervention being described. **Reviewers** – use '?' if information about the element is not reported/not sufficiently reported.

† If the information is not provided in the primary paper, give details of where this information is available. This may include locations such as a published protocol or other published papers (provide citation details) or a website (provide the URL).
‡ If completing the TIDieR checklist for a protocol, these items are not relevant to the protocol and cannot be described until the study is complete.

* We strongly recommend using this checklist in conjunction with the TIDieR guide (see *BMJ* 2014;348:g1687) which contains an explanation and elaboration for each item.

* The focus of TIDieR is on reporting details of the intervention elements (and where relevant, comparison elements) of a study. Other elements and methodological features of studies are covered by other reporting statements and checklists and have not been duplicated as part of the TIDieR checklist. When a **randomised trial** is being reported, the TIDieR checklist should be used in conjunction with the CONSORT statement (see www.consort-statement.org) as an extension of **Item 5 of the CONSORT 2010 Statement.** When a **clinical trial protocol** is being reported, the TIDieR checklist should be used in conjunction with the SPIRIT statement as an extension of **Item 11 of the SPIRIT 2013 Statement** (see www.spirit-statement.org). For alternate study designs, TIDieR can be used in conjunction with the appropriate checklist for that study design (see www.equator-network.org).
