# Supplementary material for: Randomised controlled pilot trial of an exercise plus behaviour change intervention in people with multiple sclerosis: the Step it Up study
Source: BMJ Open. 2017 Oct 12;7(10):e016336. doi: 10.1136/bmjopen-2017-016336 (PMC5654468; doi:10.1136/bmjopen-2017-016336)
Supplement: Author's manuscript [file bmjopen-2017-016336.draft_revisions.pdf]

BMJ Open is committed to open peer review. As part of this commitment we make the peer review history of every article we publish publicly available.

When an article is published we post the peer reviewers' comments and the authors' responses online. We also post the versions of the paper that were used during peer review. These are the versions that the peer review comments apply to.

The versions of the paper that follow are the versions that were submitted during the peer review process. They are not the versions of record or the final published versions. They should not be cited or distributed as the published version of this manuscript.

BMJ Open is an open access journal and the full, final, typeset and author-corrected version of record of the manuscript is available on our site with no access controls, subscription charges or pay-per-view fees (http://bmjopen.bmj.com).

If you have any questions on BMJ Open's open peer review process please email editorial.bmjopen@bmj.com

BMJ Open

A randomised controlled pilot trial of an exercise plus behaviour change intervention in people with multiple sclerosis: the Step it Up study

Journal:	*BMJ Open*
Manuscript ID	bmjopen-2017-016336
Article Type:	Research
Date Submitted by the Author:	07-Feb-2017
Complete List of Authors:	Hayes, Sara; University of Limerick Faculty of Education and Health Sciences<br>Uszynski, Marcin; University of Limerick, Clinical Therapies<br>Motl, Robert; University of Alabama at Birmingham<br>Larkin, Aidan; Multiple Sclerosis Society of Ireland<br>Gallagher, Stephen; University of Limerick, Department of Psychology<br>Newell, John; NUI Galway<br>Scarrott, Carl; University of Canterbury, Mathematics and Statistics<br>Coote, Susan ; University of Limerick, Clinical Therapies; University of Limerick
**Primary Subject Heading**:	Neurology
Secondary Subject Heading:	Rehabilitation medicine
Keywords:	exercise, walking mobility, behaviour change, Multiple sclerosis < NEUROLOGY

SCHOLARONE™
Manuscripts

TITLE PAGE

A randomised controlled pilot trial of an exercise plus behaviour change intervention in people with multiple sclerosis: the Step it Up study

Sara Hayes[1]

Email: sara.hayes@ul.ie

Marcin Uszynski[1]

Email: marcin.uszynski@ul.ie

Robert W. Motl[5]

Email: robmotl@uab.edu

Stephen Gallagher[2]

Email: stephen.gallagher@ul.ie

Aidan Larkin[3]

Email: aidanL@MS-Society.ie

John Newell[4]

Email: john.newell@nuigalway.ie

Carl Scarrott[6]

Email: carl.scarrott@canterbury.ac.nz

*Susan Coote[1]

Email: susan.coote@ul.ie

[1] Department of Clinical Therapies, Faculty of Education and Health Sciences, Health Research Institute, University of Limerick, Limerick, Ireland

[2] Centre for Social Issues Research, Department of Psychology, Faculty of Education and Health Sciences, University of Limerick, Limerick, Ireland

[3] Multiple Sclerosis Society of Ireland, Dublin, Ireland

[4] HRB Clinical Research Facility and School of Mathematics, Statistics and Applied Mathematics, National University of Ireland, Galway, Ireland

For peer review only

For peer review only - http://bmjopen.bmj.com/site/about/guidelines.xhtml

[5] Department of Physical Therapy, School of Health Professions, University of Alabama at Birmingham

[6] HRB Clinical Research Facility, National University of Ireland, Galway, Ireland and School of Mathematics and Statistics, University of Canterbury, New Zealand

*Author for correspondence

For peer review only

For peer review only - http://bmjopen.bmj.com/site/about/guidelines.xhtml

ABSTRACT

Objective: to investigate the feasibility of MS exercise guidelines for inactive people with MS and to examine the efficacy for walking. To investigate the effect of augmenting that intervention with education based on Social Cognitive Theory (SCT)

Design: pilot multicentre, double blind, randomised controlled trial

Setting: community-delivered exercise programme

Participants: Sixty-five pwMS who walked independently, scored 0–3 on the Patient Determined Disease Steps Scale, had no MS relapse or change in MS medication in 12 weeks and were physically inactive

Interventions: 10-week exercise plus SCT education compared with exercise plus attention control intervention

Outcome measures: The Six Minute Walk Test (6MWT). Timed Up and Go (TUG) test and Multiple Sclerosis Walking Scale-12 (MSWS-12) were secondary outcomes.

Results: One hundred and seventy-four expressed interest, 92 were eligible and 65 enrolled. The intervention was feasible and delivered as intended. Sixty-eight percent of SCT group and 50% of control group met the exercise guidelines after intervention.

Both groups improved significantly in 6MWT following 10-week intervention (intervention mean Δ=83.02, sd=60.1, p= <0.01, control mean Δ=56.92, sd=73.5 p=<0.01), TUG (intervention Δ=-0.70, sd=1.25, p=<0.01, control Δ=-0.54, sd=0.95, p=<0.01), and MSWS-12 (intervention Δ=-8.03, sd=16.18, p=0.02, control Δ=-0.86, sd=18.74, p=0.81). Using linear mixed effects models, intention to treat basis, there was insufficient evidence for difference between the groups over the trial (6MWT p=0.30, TUG p=0.4, MSWS p=0.8). Using per protocol analysis, there was significant treatment effect favouring the intervention group (p=0.04) with mean effect for 6MWT 39.0m (95%CI 2.26, 75.73) at 12 weeks and 40.0m (95%CI 2.3, 77.8) at 36 weeks

For peer review only - http://bmjopen.bmj.com/site/about/guidelines.xhtml

Conclusions: Positive effects of 10-week exercise programme based on the MS exercise guidelines for improving walking in previously inactive people with MS were demonstrated. There is marginal evidence of a treatment effect in favour of the exercise plus SCT intervention at 12 and 36 weeks.

Trial registration NCT02301442.

Keywords Exercise, walking mobility, Social Cognitive Theory, behaviour change, multiple sclerosis

For peer review only

STRENGTHS AND LIMITATIONS

- New evidence demonstrating the feasibility and clinical efficacy of delivering a pragmatic, combined community-based exercise and theory-based Social Cognitive Theory education intervention for physically inactive people with MS based on the MS Exercise Guideline
- The use of measures of fidelity, assessments of the target variables of the intervention (strength, fitness and physical activity) and both self-report and objective measures of walking mobility
- While treatment fidelity was considered and evaluated, a limitation relates to the use of a limited 1-day training course for physiotherapists, in particular relating to the novel use of education techniques throughout the exercise programme.
- Attrition of participants between determining eligibility and starting the intervention; the long wait times meant that 29% of eligible participants were lost at this phase.

For peer review only - http://bmjopen.bmj.com/site/about/guidelines.xhtml

INTRODUCTION

Walking limitations are the hallmark of multiple sclerosis (MS)[1] and people with MS report that walking limitations are a significant concern[2]. Indeed, walking limitations are cited as the primary reason for unemployment[3] and influence a range of other outcomes such as cognition and depression[4]. Exercise training remains the cornerstone therapeutic intervention for the management of walking limitations in MS. Many studies report positive effects from a range of exercise interventions and recent reviews [5] and meta-analyses[6 7] confirm that combined aerobic and resistance exercises can improve both walking speed and walking endurance. The recent exercise guidelines recommend aerobic exercise twice a week and resistance exercise twice a week as the minimum target for improving walking outcomes among people with mild-to-moderate MS [8]. To that end, we demonstrated using a pragmatic, randomised controlled trial design, that 10 weeks of combined aerobic and resistance training delivered in groups in the community yielded positive improvements in 6MWT [9]. Of concern, however, was that these improvements were not maintained at three-month follow-up[10]. The maintenance of long-term exercise behaviour change is not a problem that is unique to MS, and researchers have highlighted the need for inclusion of behavioural approaches based on theory for long-term behaviour change [11]. Social cognitive theory (SCT) has been widely investigated in MS and its domains of exercise self-efficacy and goal setting are consistently associated with physical activity (PA) behaviour [12]. We have reported improvements in PA, and secondary outcomes including walking, from an SCT-based online intervention in MS [13], and one study demonstrated that physical activity behaviour change was maintained after cessation of the program[14]. We designed a randomised controlled pilot trial called 'Step it Up' [15] that combined a group exercise programme with a theory-based education component for augmenting the effect of exercise on walking outcomes and sustain these changes over time. The current paper reports the feasibility of this programme and preliminary clinical efficacy for improving walking outcomes; secondary outcomes will be provided in a parallel publication. We delivered the same exercise programme to both groups and controlled for contact by comparing a structured SCT education

For peer review only - http://bmjopen.bmj.com/site/about/guidelines.xhtml

programme with an attention control education programme, and investigated whether adding the SCT education component would yield greater improvements in walking mobility and whether the improvements were maintained at follow-up. It was hypothesized that that the participants in the exercise and SCT-based intervention would achieve significantly more improvement in walking outcomes than the control group post-intervention and that this improvement would be maintained at follow up.

METHODS

Study design

This was a multicentre, double blind, randomised controlled trial.

Setting and participants

The participants were recruited through the MS Society of Ireland, and via neurology clinics in three urban locations in the Republic of Ireland. Details of the recruitment process are further detailed in the protocol paper[15]. Inclusion criteria were: (1) physician-confirmed formal diagnosis of MS, (2) aged 18 years or more, (3) Patient Determined Disease Steps (PDDS) scale score of 0–3, (4) a sedentary lifestyle (<30 minutes of moderate to strenuous exercise one day or more per week over the last six months) and (5) willing to give written informed consent. Exclusion criteria included: (1) pregnancy, (2) MS relapse in the previous 12 weeks and (3) changes to MS medication or steroid treatment in the previous 12 weeks. Participants were sent the consent form in advance of the baseline assessment, and written consent was obtained in person by a blinded assessor.

Randomisation and blinding

Participants were randomly allocated into the exercise plus SCT-based intervention or the exercise plus contact control education intervention. Random allocation procedures have been previously

For peer review only - http://bmjopen.bmj.com/site/about/guidelines.xhtml

outlined[15] and were adhered to. JN generated the random allocation sequence, the SH enrolled participants, and SC assigned participants to interventions. The outcome assessor (SH) was blind to allocation throughout the study as were the statisticians (CS, JN). All participants were informed that the study aimed to examine the effect of combining exercise and education, and therefore were blinded regarding group allocation.

Screening questionnaire

Potential participants were screened for eligibility for this study using a questionnaire that included the Patient Determined Disease Steps (PDDS) scale[16], confirmation of formal MS diagnosis and questions regarding PA levels. The PDDS scale contains a single item for measuring self-reported neurological impairment on an ordinal level from zero (Normal) to eight (Bedridden). Scores from the PDDS are linearly and strongly related with physician-administered Expanded Disability Status Scale (EDSS) scores[16] .

Outcome measures

Outcome measures were conducted pre-intervention (week 1), post-intervention (week 12), and at 24- and 36-week follow-up.

Demographic and clinical information

Participants provided details regarding age, gender, level of formal education, time since diagnosis of MS, duration of symptoms of MS, falls history, exercise history, marital status and employment status. Additionally, a researcher formally trained in the use of the Expanded Disability Status Scale (EDSS) (SH) administered the EDSS to all participants at baseline. The EDSS quantifies MS disease progression and is commonly the standard that other outcome measures are compared against [17]. It consists of functional systems subscales and a total score which is an ordinal rating ranging from 0

For peer review only - http://bmjopen.bmj.com/site/about/guidelines.xhtml

For peer review only

(normal neurological status) to 10 (death due to MS). MS diagnosis according to the McDonald or Poser criteria was confirmed from the participant's consultant neurologist.

Primary outcome

The primary outcome was walking mobility. This was measured using the Six Minute Walk Test (6MWT) as the primary endpoint. The 6MWT has demonstrated excellent test-retest reliability and concurrent validity among people with mild to moderate MS[18].

Secondary outcomes

We further used the Timed Up and Go test (TUG) and the Multiple Sclerosis Walking Scale-12 (MSWS-12). The TUG has demonstrated excellent test-retest reliability for people with mild MS [19] and the MSWS-12 has demonstrated excellent internal consistency[20 21], test-retest reliability[22] and concurrent validity in people with MS[23]. For both walking tests participants were asked to "walk as quickly and safely as possible".

Adherence to the intervention was documented throughout the 10-week intervention via exercise logs. The exercise logs captured attendance at the exercise classes and home exercise sessions. Over the 10-week intervention, 44 total sessions were made available to the participants. This included six exercise classes with strengthening and coaching/education components, four coaching phone calls, 14 prescribed home strengthening sessions, and 20 prescribed home walking sessions.

The 5 times sit to stand test (5xSTS)[24], the Modified Canadian Aerobic Fitness Test (mCAFT)[25] and the Godin Health Index of the Godin Leisure-Time Exercise Questionnaire (GLTEQ)[26] were used to measure lower extremity muscle strength, aerobic capacity and PA behaviour, respectively. These measures and associated psychometric properties have been described in the trial protocol[15].

For peer review only - http://bmjopen.bmj.com/site/about/guidelines.xhtml

Interventions

The content of the interventions delivered in both arms of this RCT has been outlined in detail in the protocol paper[15]. The exercise intervention was common to both groups and was delivered by physiotherapists. The aim of the exercise component was to progressively increase the intensity of both aerobic and strengthening activities to enable the participants to reach the published exercise guidelines for people with mild-to-moderate MS[8], and has been previously described[15]. Over the 10-week programme participants attended the group exercise class on six occasions, supplemented with a telephone coaching call in the weeks without classes (intervention weeks 4, 6, 7 and 9). After each of the group exercise classes the control group received an education session about topics unrelated to PA behaviour, e.g. diet, vitamin D, sleep, temperature and hydration, and immunisations and vaccinations. The exercise plus SCT-based intervention group received the same exercise intervention as the control group (as described in the previous section). This group also received a similar duration of education based on the principles of SCT for health behaviour change, namely: self-efficacy, outcome expectations, goal-setting, barriers and benefits and has been previously described[15].

Treatment fidelity

All of the physiotherapists who delivered the intervention or control group sessions were provided with a one-day training course on the delivery of the intervention for their group, directly related to the manual of operating procedures[15]. Continued support from the research centre was available if additional training was needed. The fidelity of the physiotherapists' sessions was monitored by randomly allocated video and audio recording of at least one of the intervention sessions.

For peer review only - http://bmjopen.bmj.com/site/about/guidelines.xhtml

Statistical analysis

Sample size

Consistent with data from a large international study[27], it was assumed that the effect of the intervention would yield an average improvement in 6MWT distance of 36m with an estimated standard deviation of 48.2m. In order to have 80% power (at the 5% significance level) to detect such a difference in mean improvement in 6MWT over the study period between groups, a sample of size 62 randomised equally to two arms (i.e. 31 per arm) was needed.

Suitable numerical statistics and graphical summaries were used to describe characteristics of the sample at baseline and to assess the validity of any distributional assumptions needed for the formal analysis. All tests of significance were two-sided and conducted at an alpha = 0.05 level of statistical significance. An exploratory paired t-test between baseline and each of the week 12, 24 and 36 follow-ups are conducted, provides a summary of the effects of the estimated treatment and control from the raw data. These “unadjusted” results do not account for the patient covariates and repeated measurements.

The statistical modelling compared differences in the response variables (6MWT, TUG and MSWS scores) between the two intervention arms at each of the three post-intervention follow-ups while correcting for the baseline measurements for each participant. A linear mixed model for a continuous response over time due to the two interventions, whilst adjusting for participant-specific covariates and factors; namely 6MWT at baseline, age, gender, time since diagnosis and MS type (i.e. benign, primary progressive and relapsing-remitting). Treatment and time (and their interaction) were specified as fixed effects, centre (three levels) and subject (nested in centre) as random effects in order to account for homogeneity within centre and within subject correlation over time. Initially a model containing the main effects of the treatment, time and a treatment-by-time interaction was

For peer review only - http://bmjopen.bmj.com/site/about/guidelines.xhtml

specified in order to test whether there is evidence that the treatment effects varies over time. If the interaction was deemed unnecessary (using a likelihood ratio test) the model was refitted excluding the interaction term, so the treatment effect was then constant over time. All analyses were carried out according to the intention-to-treat principle using all available measurements and then to a per-protocol cohort, defined as having at least two follow up measurements over time. All models were fitted in R 3.2.0 using the lme4 and lmerTest packages. Model diagnostics involved suitable plots of the residuals.

For peer review only

RESULTS

Participant sample

One hundred and seventy-four people with MS contacted the trial centre and were screened for inclusion over the phone between September 2013 and May 2014. Eighty-two people were excluded as per the selection criteria (Figure 1) and recruitment ceased when 92 people were randomised to either of the trial arms. Between time of randomisation and initiation of the intervention, 27 eligible participants either became in-eligible or were unable to participate. One participant was not treated as randomised (two acquaintances had been randomised to the other group and she wanted to exercise with them). Sixty-five participants commenced the intervention (SCT group n=33, CON group n=32). In the SCT group, four participants discontinued the intervention and 12 were lost to follow-up at 36-weeks. In the CON group, three participants discontinued the intervention and 10 were lost-to-follow up at 36 weeks. Following the 10-week intervention overall attrition was 17% and at the 36-week follow-up assessment attrition was 34%. Reasons for discontinuing the intervention and loss to follow-up are outlined in Figure 1. Baseline characteristics for both groups are shown in Table 1.

For peer review only - http://bmjopen.bmj.com/site/about/guidelines.xhtml

Table 1 Clinical baseline characteristics in exercise plus SCT group (SCT) and exercise plus education control group (CON)

	SCT (n=33)	CON (n=32)
**MS type**		
Benign	3	1
Primary progressive	1	0
Relapsing-remitting	27	27
Secondary progressive	0	1
Unknown	2	2
**EDSS (median, IQR)**	3.3 (0.7)	3.3 (0.7)
**Years since diagnosis**	6.7 (5.7)	7.0 (6.1)
**Centre (n)**		
Cork	10	9
Galway	8	10
Limerick	15	13
**Age**	43.3 (9.9)	41.9 (9.3)
**Gender (n)**		
Male	4	6
Female	29	26

EDDS: Expanded Disability Disease Scale; IQR: interquartile range; Data given as mean (SD) unless otherwise indicated

Treatment fidelity

An independent person to the intervention (PO'S) used the manual of operating procedures to check if the required content of the programme was delivered as intended. In both trial arms, 100% of the content of the supervised sessions were implemented as described in the intervention manual.

Feasibility - Exercise Logs

The development of hip pain by one participant in the CON group was the only adverse event reported by participants in both trial arms during the completion of the 10-week intervention. The SCT and the CON group groups completed an average of 33.2 of 44 available sessions (75.5%) and 32.0 sessions (72.6%), respectively. The proportion of sessions completed is presented in Figure 2, wherein the lowest number of sessions was in week 7 when participants were exercising independently without a class for a second consecutive week. Among the 53 participants who provided detailed exercise logs, 17 (68%) of the SCT group and 14 (50%) of the CON group were

For peer review only - http://bmjopen.bmj.com/site/about/guidelines.xhtml

exercising at the minimum recommended by the exercise guidelines by the end of the 10-week intervention. The reasons for not meeting the guideline included: walking less than 30 minutes twice per week (SCT n=3, CON n=1), walking only once per week (SCT n=2 CON n=5) and doing only one set of each resistance exercise per week (SCT n=2, CON n=4).

In order to further evaluate the effect of this strength and aerobic intervention we investigated the change in strength, fitness and physical activity. Table 2 presents the raw data and unadjusted comparisons. For both groups there were significant improvements in PA and strength from weeks 1 to 12. There was a tendency for aerobic fitness scores to increase, but this change was not statistically significant.

Table 2 Raw data and unadjusted comparisons of change in secondary outcomes from week 1 to week 12 in exercise plus SCT group (SCT) and exercise plus education control group (CON)

		**Week 1 Mean (SD)**	**Week 12 Mean (SD)**	**Mean change from week 1 to week 12 (95% CI) *p*-value**
**Godin Health Index**	SCT	3.03 (6.19)	12.48 (11.15)	9.85 (5.46, 14.23) $p<0.01$
	CON	1.88 (4.88)	16.07 (21.12)	12.92 (4.96, 20.89) $p<0.01$
**Five Times Sit to Stand**	SCT	11.48 (2.7)	9.78 (2.18)	-1.51 (-2.42, -0.60) $p<0.01$
	CON	10.8 (2.6)	9.43 (1.93)	-1.55 (-2.30, -0.79) $p<0.01$
**Aerobic Fitness Score**	SCT	295.72 (54.61)	309.12 (53.78)	8.58 (-6.86, 23.98) $p=0.26$
	CON	313.56 (59.02)	331.29 (51.57)	10.54 (-6.29, 27.37) $p=0.21$

CI: confidence interval

For peer review only - http://bmjopen.bmj.com/site/about/guidelines.xhtml

Walking mobility

The mean (SD) scores for the 6MWT, TUG and MSWS-12 at weeks 1, 12, 24 and 36 for participants in the exercise plus SCT and exercise plus education control groups are presented in Table 3. Figure 3 shows the results of the estimated treatment effects on 6MWT, TUG and MSWS-12, as per intention-to-treat and per-protocol analyses, respectively. The unadjusted, unstandardized mean changes from baseline, and 95% confidence intervals and paired t-test results for both groups are presented in Table 4. Both groups demonstrated an improvement in the primary outcome, 6MWT and secondary outcome MSWS from weeks one to 12, 24 and 36. For TUG the result are a little more mixed, with evidence of an improvement in both groups from weeks one to 12 which diminishes in the control group by week 36 but a persistent significant difference is observed in the education with SCT group from baseline to weeks 24 and 36.

Table 3 Mean (SD) walking mobility outcomes at weeks 1, 12, 24 and 36 in exercise plus SCT group (SCT) and exercise plus education control group (CON)

	**Week 1 mean (SD)**		**Week 12 mean (SD)**		**Week 24 mean (SD)**		**Week 36 mean (SD)**	
Intention-to-treat analysis								
**Outcome variable**	**SCT**	**CON**	**SCT**	**CON**	**SCT**	**CON**	**SCT**	**CON**
**6MWT**	445.2 (68.8)	482.0 (72.0)	527.4 (91.1)	547.1 (96.0)	492.8 (73.5)	504.9 (76.9)	515.8 (91.0)	528.0 (93.2)
**TUG**	7.06 (1.61)	6.51 (1.36)	6.27 (1.45)	5.81 (1.08)	6.23 (1.26)	6.00 (0.98)	5.93 (1.33)	5.96 (1.20)
**MSWS-12**	38.0 (28.0)	33.3 (24.8)	29.6 (22.2)	30.8 (21.3)	31.9 (22.1)	26.3 (21.5)	32.6 (23.4)	27.9 (21.9)
Per-protocol analysis								
	**SCT**	**CON**	**SCT**	**CON**	**SCT**	**CON**	**SCT**	**CON**
**6MWT**	434.6 (65.2)	474.4 (69.6)	524.2 (96.7)	535.2 (88.0)	496.2 (73.7)	504.9 (76.9)	515.8 (91.0)	528.0 (93.2)
**TUG**	7.08 (1.73)	6.65 (1.36)	6.43 (1.46)	5.87 (1.13)	6.30 (1.25)	6.00 (0.98)	5.93 (1.33)	5.96 (1.20)
**MSWS-12**	38.2 (26.7)	31.9 (22.6)	29.7 (22.6)	32.6 (21.0)	31.9 (22.1)	26.3 (21.5)	32.6 (23.4)	27.0 (21.8)

6MWT: Six Minute Walk Test; TUG: Timed Up and Go; MSWS-12: Multiple Sclerosis Walking Scale-12

For peer review only - http://bmjopen.bmj.com/site/about/guidelines.xhtml

Table 4 Unadjusted comparisons of change in walking measures from week 1 to weeks 12, 24 and 36 in exercise plus SCT group (SCT) and exercise plus education control group (CON)

	**Mean change week 1 to week 12 (95% CI) *p*-value**		**Mean change week 1 to week 24 (95% CI) *p*-value**		**Mean change week 1 to week 36 (95% CI) *p*-value**	
	**SCT**	**CON**	**SCT**	**CON**	**SCT**	**CON**
**6MWT**	83.02 (58.74, 107.29) *p*<0.01	56.92 (28.43, 85.41) *p*<0.01	55.97 (32.12, 79.84) *p*<0.01	34.2 (13.43, 54.97) *p*<0.01	82.18 (50.90, 113.45) *p*<0.01	46.87 (18.57, 75.17) *p*<0.01
**TUG**	-0.70 (-1.20, -0.19) *p*<0.01	-0.54 (-0.91, -0.17) *p*<0.01	-0.79 (-1.19, -0.38) *p*<0.01	-0.74 (-1.13, -0.35) *p*<0.01	-1.23 (-1.68, -0.78) *p*<0.01	-0.57 (-0.98, -0.16) *p*<0.01
**MSWS-12**	-8.03 (-14.43, -1.63) *p*=0.02	-0.86 (-7.99, 6.27) *p*=0.81	-6.43 (-12.10, -0.77) *p*=0.03	-2.88 (-11.41, 5.64) *p*=0.49	-8.62 (-15.90, -1.34) *p*=0.02	-5.60 (-13.84, 2.64) *p*=0.17

6MWT: Six Minute Walk Test; TUG: Timed Up and Go; MSWS-12: Multiple Sclerosis Walking Scale-12

The linear mixed models results in Table 5 shows that using an intention-to-treat analysis there was no evidence of a significant treatment effect in favour of the exercise plus SCT compared to the exercise only group for regarding 6MWT, TUG or MSWS scores. Figure 3 confirms the obvious significant effects of the exercise programme found above in the unadjusted paired t-test results, which is shown by the blue and red lines being well above the black “no effect” line when the sample uncertainty conveyed by the corresponding confidence intervals are taken into account. But Figure 3 also confirms lack of evidence for an additional effect of the SCT over the usual exercise programme, which is shown by the widely overlapping confidence intervals between the treatment and control groups.

For peer review only

Table 5 Estimated treatment effects at weeks 12, 24 and 36 in primary outcome

	Estimate of difference between SCT and Control	Standard error	95% CI	*p*-value
Intention-to-treat analysis				
**6MWT**				
Week 12	22.70	19.00	(-15.14, 60.50)	*0.23*
Week 24	11.80	20.40	(-28.77, 52.36)	*0.56*
Week 36	27.42	20.35	(-13.06, 67.90)	*0.18*
**TUG**				
Week 12	0.069	0.236	(-0.402, 0.541)	*0.77*
Week 24	-0.132	0.250	(-0.630, 0.365)	*0.60*
Week 36	-0.457	0.252	(-0.960, 0.045)	*0.08*
**MSWS-12**				
Week 12	-4.91	4.47	(-13.82, 4.00)	*0.28*
Week 24	-0.59	4.69	(-9.91, 8.73)	*0.90*
Week 36	0.38	4.57	(-8.71, 9.47)	*0.93*
Per-protocol analysis				
**6MWT**				
Week 12	39.00	18.44	(2.26, 75.73)	*0.04*
Week 24	27.44	19.23	(-10.82, 65.70)	*0.16*
Week 36	40.03	18.97	(2.27, 77.79)	*0.04*
**TUG**				
Week 12	0.204	0.255	(-0.306, 0.713)	*0.43*
Week 24	-0.020	0.261	(-0.542, 0.502)	*0.94*
Week 36	-0.367	0.262	(-0.890, 0.156)	*0.17*
**MSWS-12**				
Week 12	-7.63	4.65	(-16.89, 1.63)	*0.11*
Week 24	-2.50	4.78	(-12.01, 7.02)	*0.60*
Week 36	-1.57	4.69	(-10.93, 7.78)	*0.74*

6MWT: Six Minute Walk Test; TUG: Timed Up and Go; MSWS-12: Multiple Sclerosis Walking Scale-12

Per-protocol analysis was completed with participants who attended at least two of the three follow-up assessments. Table 3 presents the mean (SD) scores for the 6MWT, TUG and MSWS-12 at weeks 1, 12, 24 and 36 for participants in the SCT and control groups using per-protocol analysis. For 6MWT, the SCT group had a marginally more positive outcome, with statistically significant treatment effects evident at weeks 12 and 36 (Table 5). Using per protocol analysis there was no evidence of a treatment effect in favour of the SCT group as compared to the CON group regarding the TUG or MSWS-12 scores.

For peer review only - http://bmjopen.bmj.com/site/about/guidelines.xhtml

DISCUSSION

This pilot RCT investigated the feasibility and preliminary efficacy of the Step it Up programme, a 10-week aerobic and strengthening programme that aimed to enable physically inactive people with MS to exercise according to the recent MS exercise guidelines [8]. We investigated whether embedding an evidence-based exercise programme within a structured SCT-based education programme resulted in improved and more sustained walking outcomes compared to an exercise plus attention control education intervention. To our knowledge, this is the first study to examine the effect of enabling inactive people to meet the minimum recommended dose of the MS exercise guidelines *and* examine the effects on walking mobility as a primary end-point.

The intervention protocol was feasible and results demonstrated significant improvements in walking mobility following the intervention in both groups. The effect for the SCT group was greater at 12- and 36-week follow-up for the primary outcome, 6MWT, using a per-protocol analysis. Recruitment was successful and over nine months at three centres we recruited more than our target of 62 participants (92 eligible participants). The largest point of attrition was while participants waited for enough people to run their group in that region. In the future, recruiting from the largest city in Ireland for a definitive RCT will enable greater numbers to be recruited more quickly and should minimise this attrition at this point in the trial. Retention across the intervention period was good and the attrition rate (17%) was similar to other exercise interventions in people with depression[28] and slightly higher than the average of 15% in a review of exercise trials in MS[29]. While the level of participant attrition in the current programme is greatly improved from our previous community based exercise randomised controlled trial[9 10], measures such as recruiting a dedicated study coordinator to provide more frequent interactions with participants in the definitive trial will be explored to further enhance retention at follow-up. The addition of booster intervention sessions after the completion of the 10-week intervention will also be explored in the future definitive trial.

For peer review only - http://bmjopen.bmj.com/site/about/guidelines.xhtml

The intervention was delivered by physiotherapists who attended a 1-day training session and treatment fidelity findings suggest that this approach was successful as the interventions were delivered as intended; further training and support may increase the success of the intervention in future. Participants completed on average 73 to 75% of possible sessions suggesting that the protocol is feasible for participants with minimal impairment due to MS. We collected data from exercise logs for demonstrating adherence with the exercise programs. The exercise logs were returned by 82% of participants and used to ascertain whether participants were meeting the MS exercise guideline at the end of the intervention period. It is interesting to note that a greater proportion of participants in the SCT group (68% versus 50%) progressed to meeting the guidelines. Measures to further enhance completion and return of logs (such as offering them in alternative electronic formats) in the future definitive trial are needed.

We further confirmed adherence to this aerobic and strengthening intervention by investigating its effects on strength, fitness and PA. Both 5xSTS and Godin Health index increased significantly and the AFS showed a tendency to improve providing evidence that the exercise intervention met its intended outcomes. Collectively, we believe that the exercise log data combined with fitness and PA outcomes support the successful manipulation of exercise behaviour with in both trial arms. Based on the data on recruitment, retention, feasibility and preliminary efficacy of this group exercise and SCT education intervention we propose to progress to a definitive intervention. To do this, a sample of 49 (for a difference between groups of 39m, assuming a standard deviation of the change score at 36 weeks of 67.85, 80% power, 0.05 significance level) in each group would be needed and we therefore plan to recruit across these three centres again and to add a 4th centre in the largest city in Ireland to minimise attrition.

Importantly, both groups improved significantly in the primary outcome, 6MWT, following the intervention. This improvement in 6MWT is consistent with a recent systematic review of exercise studies that found a significant improvement in walking endurance[7]. We note that the mean

For peer review only - http://bmjopen.bmj.com/site/about/guidelines.xhtml

For peer review only

improvement in the SCT group of 80m and of 60m in the control group far exceeded the value for the clinically important change of 26.1m proposed by (Baert et al 2014)[27]. Both groups improved more than that reported by Carter et al (2012)[30] in their exercise plus education group, and the magnitude of improvement is more consistent with the improvements noted in a recent community-based intervention among people with moderate-severity MS[31]. We further note that the current physically inactive sample of people with MS with an average age of 42 had 6MWT of 445m at baseline that was less than that of a reference sample aged 70-80 years who walked an average of 514m[32]. This confirms the significant walking impairments for inactive people with mild disability with MS and importantly demonstrates positive improvements due to the Step it Up exercise intervention. Interestingly the SCT group but not the CON group improved in their self-reported walking impairment (MSWS-12) and the magnitude of the change in 6MWT distance may have influenced that finding. Both groups however improved in walking speed and maintained that improvement at 36 week follow-up.

Of note, through the per-protocol analysis including participants who participated in at least two follow-up assessments, we demonstrated that adding a structured SCT education programme enhanced the effect on 6MWT distance following the 10-week intervention. Additionally, both the improvement from baseline and the difference in between-group effects were maintained at 36-week follow-up. The SCT education programme had six education sessions that targeted outcome expectancies, self-efficacy, goal setting, and perceived barriers and benefits of exercise. This education program was originally designed based on a RCT of a SCT-based exercise intervention delivered in older adults [33] and later modified and tested for MS [34]. The components are further consistent with a recent systematic review and meta-analysis of modifiable psychosocial constructs associated with PA in MS that confirmed self-efficacy, goal setting and outcome expectancies as significantly correlated with PA in MS[35]. One novel feature of the current trial is that the SCT education modules were delivered by physiotherapists with minimal training in delivery of

For peer review only - http://bmjopen.bmj.com/site/about/guidelines.xhtml

behavioural interventions. These findings also support that delivering this SCT education intervention by physiotherapists in a group setting is both feasible and preliminary findings suggest that it has superior outcomes to an attention control education intervention.

Strengths and Limitations

One of the strengths of this pilot RCT relates to the production of new knowledge around the sustainability of exercise interventions for people with MS. Building on the existing evidence base, we designed and delivered a SCT-based pragmatic physiotherapist-led community exercise. Results demonstrated the feasibility of the protocol among physically-inactive people with mild MS and trends towards clinical efficacy for walking outcomes. Further strengths relate to the use of measures of treatment fidelity, target variables of the intervention (strength, fitness and PA) and both self-report and objective measures of walking. Additionally, in the context of an evidence base wherein PA interventions are often not theoretically-based, a key strength of this RCT is the use of the SCT framework to design a behaviour-change intervention; building on the extensive work of the US partner in this trial.

One limitation is the attrition of participants between point of eligibility and allocation to the intervention. The large waiting times resulted in the loss of 29% of eligible participants at this point in the trial. Recruitment from larger urban areas with greater numbers of both MS clinics and people with MS is planned for the future definitive trial so that the numbers required to run group classes are met more quickly. A further positive is that we used pedometers and exercise logs to record the intensity and duration of the intervention; however another limitation is that detailed exercise diaries were not returned for all participants. However, a return rate of 82% is acceptable and measures to improve this in the definitive trial will be considered.

Conclusion

For peer review only - http://bmjopen.bmj.com/site/about/guidelines.xhtml

This pilot RCT aimed to investigate the feasibility and preliminary efficacy of enabling physically inactive people with MS to meet the MS exercise guidelines [8] through a group exercise and education, physiotherapist-led intervention. We further sought to investigate whether the theory-based SCT component was superior to an attention control education intervention. We found that recruitment was successful, though measures to improve retention in a future definitive trial are needed. Attrition over the intervention and follow-up periods were improved compared to our previous exercise trial [9]. The programme resulted in significant improvements in walking endurance and speed for both groups. Using a per-protocol analysis there was a significant effect in favour of the exercise plus SCT groups compared to the exercise plus control education group at weeks 12 and 36. This supports the preliminary sustained efficacy of the intervention and we propose progressing to a definitive intervention.

For peer

Contributors

SH was a post-doctoral researcher on the trial, contributed to the design of the study, collected data, drafted the paper and approved the final version. MU was a post-doctoral researcher on the trial, commented on drafts of the paper and approved the final version. RM co-initiated the project and contributed to the design of the trial, drafted the paper and approved the final version. SG contributed to the design, delivery and evaluation of the trial, commented on drafts of the paper and approved the final version. AL contributed to the recruitment strategy employed, commented on drafts of the paper and approved the final version. JN and CS were the statisticians on the trial, cleaned and analysed the data, commented on drafts of the paper and approved the final version. SC was the principal investigator for the study, co-initiated the project, contributed to the design of the trial, drafted the paper and approved the final version.

Funding This work is supported by the Irish Health Research Board Health Research Award, grant number: HRA_PHR/2013-264.

For peer review only - http://bmjopen.bmj.com/site/about/guidelines.xhtml

Acknowledgements The authors would like to thank MS Ireland for their assistance with recruitment and running this trial. We would also like to thank Paraic O'Suilleabhain who assisted with data for this paper.

Competing interests All authors have completed the ICMJE uniform disclosure form at www.icmje.org/coi_disclosure.pdf and declare: no support from any organisation for the submitted work; no financial relationships with any organisations that might have an interest in the submitted work in the previous three years; no other relationships or activities that could appear to have influenced the submitted work.

Ethics approval was given by the Faculty of Education and Health Science Research Ethics Committee, University of Limerick (2014_02_20_EHS), in addition to the Research Ethics Committees at the University College Hospital Galway, University Hospital Limerick and Cork University Hospital.

Trial registration NCT02301442

Data sharing statement All data requests pertaining to the Step it Up trial should be made directly to susan.coote@ul.ie

Figure Legends

Figure 1 CONSORT Flow Diagram

Figure 2 Proportion of participants completing sessions (Exercise Diary data).

Figure 3 – Estimated effects on primary and secondary measures using intention to treat and per protocol analysis

For peer review only - http://bmjopen.bmj.com/site/about/guidelines.xhtml

REFERENCES

1. Motl RW. Ambulation and Multiple Sclerosis. *Physical Medicine and Rehabilitation Clinics of North America* 2013;24(2):325-36. doi: http://dx.doi.org/10.1016/j.pmr.2012.11.004
2. Heesen C, Bohm J, Reich C, et al. Patient perception of bodily functions in multiple sclerosis: gait and visual function are the most valuable. *Mult Scler* 2008;14(7):988-91.
3. Goldman MD, Motl RW, Scagnelli J, et al. Clinically meaningful performance benchmarks in MS: timed 25-foot walk and the real world. *Neurology* 2013;81(21):1856-63.
4. Learmonth YC, Ensari I, Motl RW. Cognitive motor interference in multiple sclerosis: Insights from a systematic, quantitative review. *Arch Phys Med Rehabil* 2016 doi: 10.1016/j.apmr.2016.07.018 [published Online First: 2016/08/21]
5. Latimer-Cheung AE, Pilutti LA, Hicks AL, et al. Effects of exercise training on fitness, mobility, fatigue, and health-related quality of life among adults with multiple sclerosis: A systematic review to inform guideline development. *Archives Of Physical Medicine And Rehabilitation* 2013;94(9):1800-28. doi: 10.1016/j.apmr.2013.04.020
6. Learmonth YC, Ensari I, Motl RW. Physiotherapy and walking outcomes in adults with multiple sclerosis: systematic review and meta-analysis. *Physical Therapy Reviews* 2016:1-13. doi: 10.1080/10833196.2016.1263415
7. Pearson M, Dieberg G, Smart N. Exercise as a therapy for improvement of walking ability in adults with multiple sclerosis: a meta-analysis. *Arch Phys Med Rehabil* 2015;96(7):1339-48.
8. Latimer-Cheung AE, Martin Ginis KA, Hicks AL, et al. Development of evidence-informed physical activity guidelines for adults with multiple sclerosis. *Arch Phys Med Rehabil* 2013;94(9):1829-36.
9. Garrett M, Hogan N, Larkin A, et al. Exercise in the community for people with minimal gait impairment due to MS: an assessor-blind randomized controlled trial. *Mult Scler* 2013;19(6):782-9.
10. Garrett M, Hogan N, Larkin A, et al. Exercise in the community for people with multiple sclerosis--a follow-up of people with minimal gait impairment. *Mult Scler* 2013;19(6):790-8.
11. Michie S, van Stralen MM, West R. The behaviour change wheel: a new method for characterising and designing behaviour change interventions. *Implementation Science* 2011;6(1):42.
12. Dlugonski D, Wojcicki TR, McAuley E, et al. Social cognitive correlates of physical activity in inactive adults with multiple sclerosis. *Int J Rehabil Res* 2011;34(2):115-20.
13. Motl RW, Dlugonski D, Wojcicki TR, et al. Internet intervention for increasing physical activity in persons with multiple sclerosis. *Mult Scler* 2011;17(1):116-28.
14. Dlugonski D, Motl RW, Mohr DC, et al. Internet-delivered behavioral intervention to increase physical activity in persons with multiple sclerosis: Sustainability and secondary outcomes. *Psychology, Health & Medicine* 2012;17(6):636-51.
15. Coote S, Gallagher S, Msetfi R, et al. A randomised controlled trial of an exercise plus behaviour change intervention in people with multiple sclerosis: the step it up study protocol. *BMC Neurol* 2014;14(241):014-0241.
16. Hadjimichael O, Kerns RD, Rizzo MA, et al. Persistent pain and uncomfortable sensations in persons with multiple sclerosis. *Pain* 2007;127(1-2):35-41.
17. Goldman MD, Motl RW, Rudick RA. Possible clinical outcome measures for clinical trials in patients with multiple sclerosis. *Ther Adv Neurol Disord* 2010;3(4):229-39. doi: 10.1177/1756285610374117 [published Online First: 2010/12/24]
18. Fry DK, Pfalzer LA. Reliability of four functional tests and rating of perceived exertion in persons with multiple sclerosis. *PHYSIOTHERAPY CANADA* 2006;58(3):212-20.
19. Nilsagard Y, Lundholm C, Gunnarsson L-G, et al. Clinical relevance using timed walk tests and'timed up and go'testing in persons with multiple sclerosis. *Physiotherapy Research International* 2007;12(2):105-14.

For peer review only - http://bmjopen.bmj.com/site/about/guidelines.xhtml

20. Hobart J, Riazi A, Lamping D, et al. Measuring the impact of MS on walking ability The 12-Item MS Walking Scale (MSWS-12). *Neurology* 2003;60(1):31-36.
21. McGuigan C, Hutchinson M. The multiple sclerosis impact scale (MSIS-29) is a reliable and sensitive measure. *Journal of neurology, neurosurgery, and psychiatry* 2004;75(2):266-69.
22. Motl RW, McAuley E, Mullen S. Longitudinal measurement invariance of the multiple sclerosis walking scale-12. *Journal Of The Neurological Sciences* 2011;305(1):75-79.
23. Cavanaugh JT, Gappmaier VO, Dibble LE, et al. Ambulatory activity in individuals with multiple sclerosis. *Journal of Neurologic Physical Therapy* 2011;35(1):26-33.
24. Csuka M, McCarty DJ. Simple method for measurement of lower extremity muscle strength. *Am J Med* 1985;78(1):77-81.
25. Kuspinar A, Andersen RE, Teng SY, et al. Predicting exercise capacity through submaximal fitness tests in persons with multiple sclerosis. *Archives Of Physical Medicine And Rehabilitation* 2010;91(9):1410-17.
26. Godin G, Shephard RJ. A simple method to assess exercise behavior in the community. *Can J Appl Sport Sci* 1985;10(3):141-6.
27. Baert I, Freeman J, Smedal T, et al. Responsiveness and clinically meaningful improvement, according to disability level, of five walking measures after rehabilitation in multiple sclerosis a European multicenter study. *Neurorehabilitation and neural repair* 2014:1545968314521010.
28. Stubbs B, Vancampfort D, Rosenbaum S, et al. Dropout from exercise randomized controlled trials among people with depression: A meta-analysis and meta regression. *Journal of affective disorders* 2016;190:457-66.
29. Pilutti LA, Platta ME, Motl RW, et al. The safety of exercise training in multiple sclerosis: A systematic review. *Journal Of The Neurological Sciences* 2014
30. Carter A, Daley A, Humphreys L, et al. Pragmatic intervention for increasing self-directed exercise behaviour and improving important health outcomes in people with multiple sclerosis: a randomised controlled trial. (1477-0970 (Electronic))
31. Learmonth YC, Paul L Fau - Miller L, Miller L Fau - Mattison P, et al. The effects of a 12-week leisure centre-based, group exercise intervention for people moderately affected with multiple sclerosis: a randomized controlled pilot study. (1477-0873 (Electronic))
32. Casanova C, Celli B, Barria P, et al. The 6-min walk distance in healthy subjects: reference standards from seven countries. *European Respiratory Journal* 2011;37(1):150-56.
33. McAuley E, Courneya Ks Fau - Rudolph DL, Rudolph Dl Fau - Lox CL, et al. Enhancing exercise adherence in middle-aged males and females. (0091-7435 (Print))
34. McAuley E, Motl Rw Fau - Morris KS, Morris Ks Fau - Hu L, et al. Enhancing physical activity adherence and well-being in multiple sclerosis: a randomised controlled trial. (1352-4585 (Print))
35. Casey B, Coote S, Shirazipour C, et al. Modifiable Psychosocial Constructs Associated with Physical Activity Participation in People with Multiple Sclerosis: A Systematic Review and Meta-Analysis. *Archives of Physical Medicine and Rehabilitation* 2017;in press

For peer review only

For peer review only - http://bmjopen.bmj.com/site/about/guidelines.xhtml

Figure 1 ***CONSORT Flow Diagram,***

DNA: did not attend

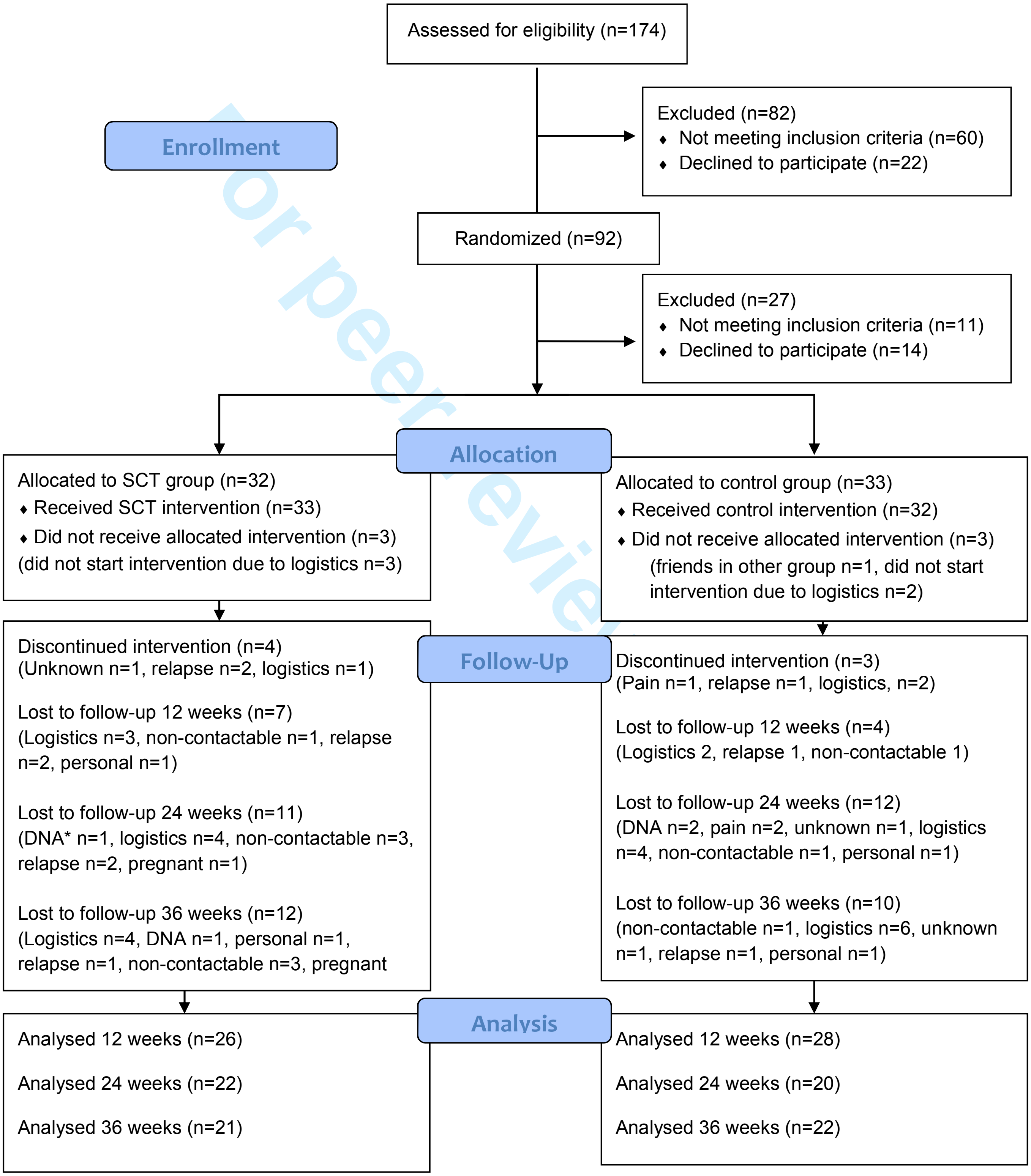

For peer review only - http://bmjopen.bmj.com/site/about/guidelines.xhtml

Figure 2 Proportion of participants completing sessions (Exercise Diary data).
SCT = exercise plus social cognitive theory education group, CON = exercise plus contact control education

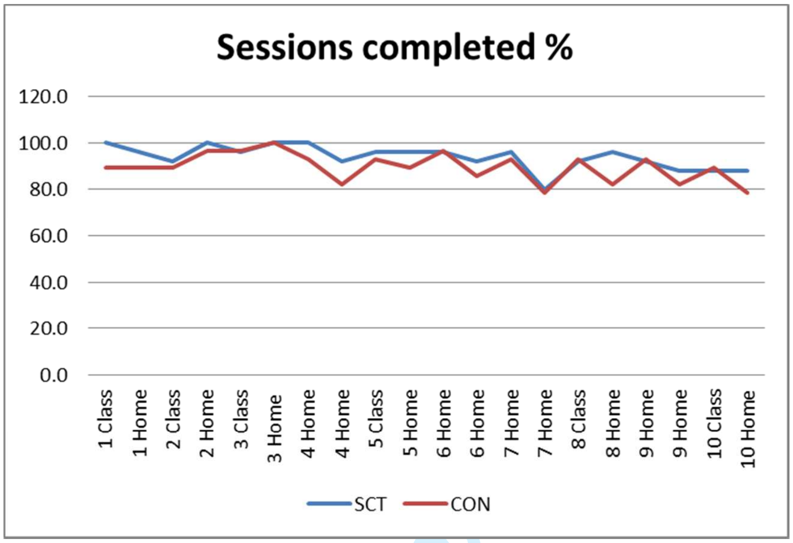

For peer review only - http://bmjopen.bmj.com/site/about/guidelines.xhtml

Figure 3 – Estimated effects on primary and secondary measures using intention to treat and per protocol analysis

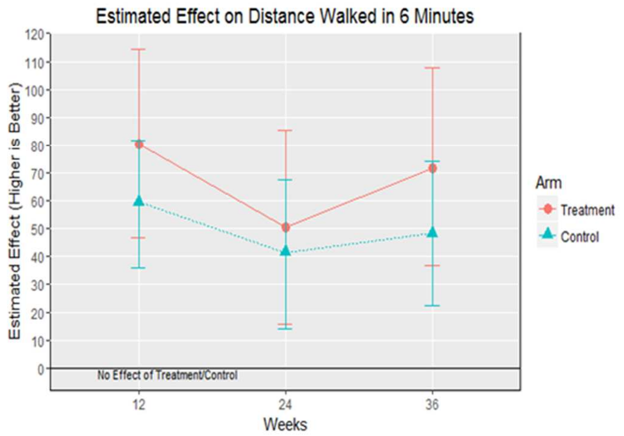

a Six Minute Walk Test estimated effect (Intention-to-treat analysis)

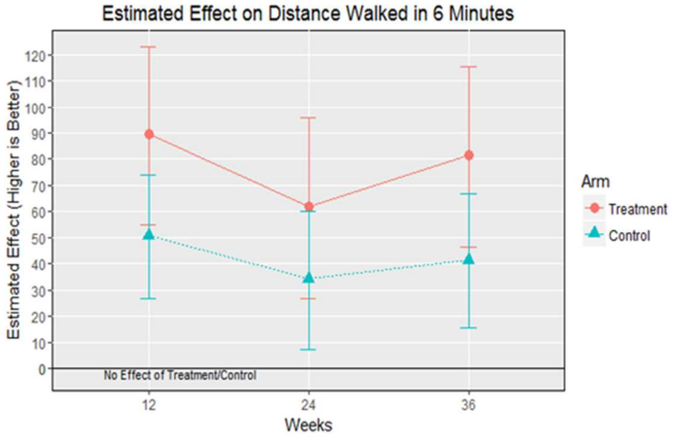

b Six Minute Walk Test estimated effect (Per protocol analysis)

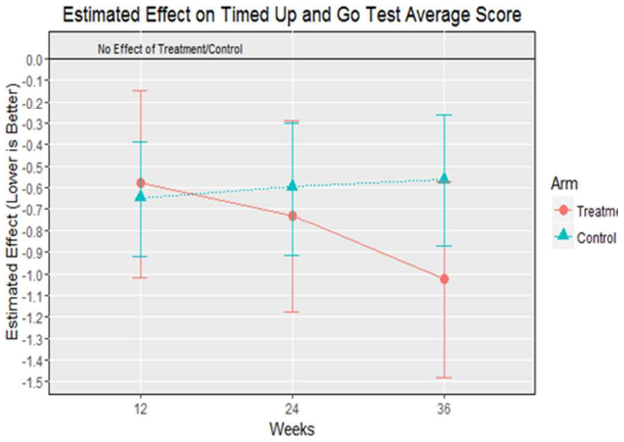

c Timed Up and Go Test estimated effect (Intention-to-treat analysis)

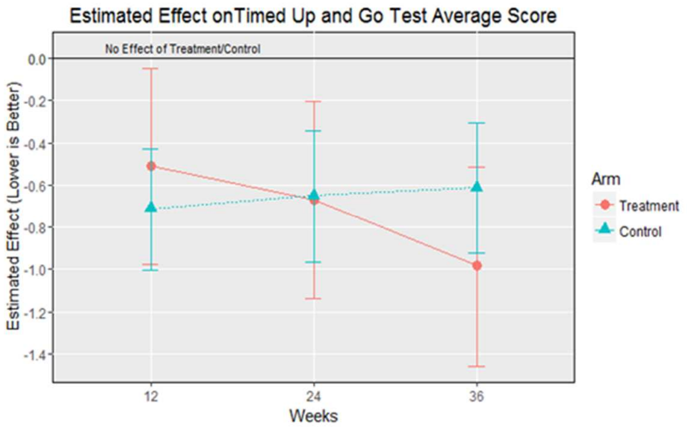

d Timed Up and Go Test estimated effect (Per protocol analysis)

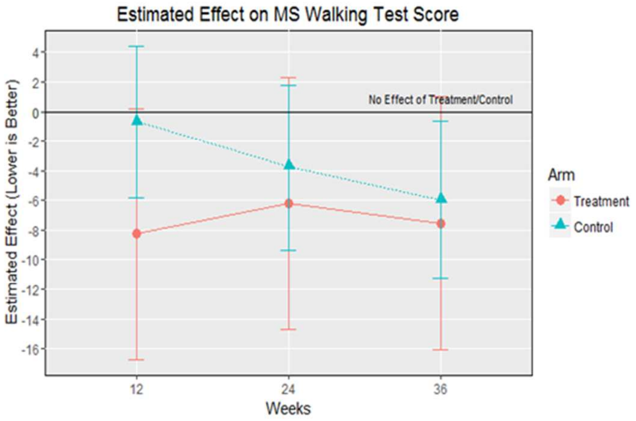

e MS Walking Scale-12 estimated effect (Intention-to-treat analysis)

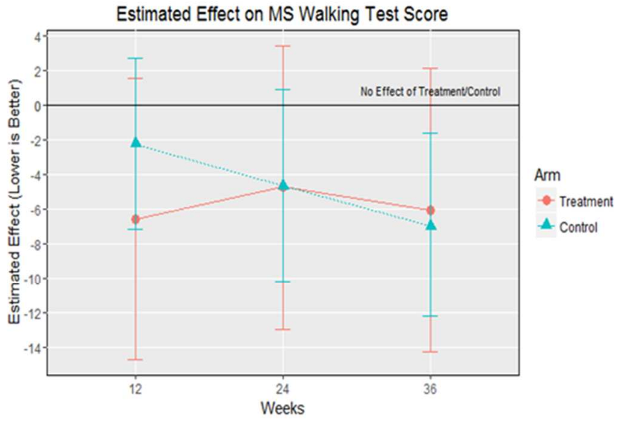

f MS Walking Scale-12 estimated effect (Per protocol analysis)

CONSORT 2010 checklist of information to include when reporting a randomised trial*

Section/Topic	Item No	Checklist item	Reported on page No
**Title and abstract**			
	1a	Identification as a randomised trial in the title	1
	1b	Structured summary of trial design, methods, results, and conclusions (for specific guidance see CONSORT for abstracts)	3
**Introduction**			
Background and objectives	2a	Scientific background and explanation of rationale	5-6
	2b	Specific objectives or hypotheses	6
**Methods**			
Trial design	3a	Description of trial design (such as parallel, factorial) including allocation ratio	6
	3b	Important changes to methods after trial commencement (such as eligibility criteria), with reasons	n/a
Participants	4a	Eligibility criteria for participants	6
	4b	Settings and locations where the data were collected	6
Interventions	5	The interventions for each group with sufficient details to allow replication, including how and when they were actually administered	9
Outcomes	6a	Completely defined pre-specified primary and secondary outcome measures, including how and when they were assessed	8
	6b	Any changes to trial outcomes after the trial commenced, with reasons	n/a
Sample size	7a	How sample size was determined	10
	7b	When applicable, explanation of any interim analyses and stopping guidelines	n/a
Randomisation:			
Sequence generation	8a	Method used to generate the random allocation sequence	6-7
	8b	Type of randomisation; details of any restriction (such as blocking and block size)	6-7
Allocation concealment mechanism	9	Mechanism used to implement the random allocation sequence (such as sequentially numbered containers), describing any steps taken to conceal the sequence until interventions were assigned	6-7
Implementation	10	Who generated the random allocation sequence, who enrolled participants, and who assigned participants to interventions	6-7
Blinding	11a	If done, who was blinded after assignment to interventions (for example, participants, care providers, those	7

For peer review only - http://bmjopen.bmj.com/site/about/guidelines.xhtml

		assessing outcomes) and how	
	11b	If relevant, description of the similarity of interventions	n/a
Statistical methods	12a	Statistical methods used to compare groups for primary and secondary outcomes	10
	12b	Methods for additional analyses, such as subgroup analyses and adjusted analyses	10
**Results**			
Participant flow (a diagram is strongly recommended)	13a	For each group, the numbers of participants who were randomly assigned, received intended treatment, and were analysed for the primary outcome	11
	13b	For each group, losses and exclusions after randomisation, together with reasons	Figure 1
Recruitment	14a	Dates defining the periods of recruitment and follow-up	11
	14b	Why the trial ended or was stopped	11
Baseline data	15	A table showing baseline demographic and clinical characteristics for each group	12
Numbers analysed	16	For each group, number of participants (denominator) included in each analysis and whether the analysis was by original assigned groups	11
Outcomes and estimation	17a	For each primary and secondary outcome, results for each group, and the estimated effect size and its precision (such as 95% confidence interval)	14-16
	17b	For binary outcomes, presentation of both absolute and relative effect sizes is recommended	n/a
Ancillary analyses	18	Results of any other analyses performed, including subgroup analyses and adjusted analyses, distinguishing pre-specified from exploratory	14-16
Harms	19	All important harms or unintended effects in each group (for specific guidance see CONSORT for harms)	12
**Discussion**			
Limitations	20	Trial limitations, addressing sources of potential bias, imprecision, and, if relevant, multiplicity of analyses	20
Generalisability	21	Generalisability (external validity, applicability) of the trial findings	21
Interpretation	22	Interpretation consistent with results, balancing benefits and harms, and considering other relevant evidence	17-21
**Other information**			
Registration	23	Registration number and name of trial registry	21
Protocol	24	Where the full trial protocol can be accessed, if available	5
Funding	25	Sources of funding and other support (such as supply of drugs), role of funders	21

*We strongly recommend reading this statement in conjunction with the CONSORT 2010 Explanation and Elaboration for important clarifications on all the items. If relevant, we also recommend reading CONSORT extensions for cluster randomised trials, non-inferiority and equivalence trials, non-pharmacological treatments, herbal interventions, and pragmatic trials. Additional extensions are forthcoming: for those and for up to date references relevant to this checklist, see www.consort-statement.org.

For peer review only - http://bmjopen.bmj.com/site/about/guidelines.xhtml

For peer review only

BMJ Open

A randomised controlled pilot trial of an exercise plus behaviour change intervention in people with multiple sclerosis: the Step it Up study

Journal:	*BMJ Open*
Manuscript ID	bmjopen-2017-016336.R1
Article Type:	Research
Date Submitted by the Author:	02-May-2017
Complete List of Authors:	Hayes, Sara; University of Limerick Faculty of Education and Health Sciences<br>Uszynski, Marcin; University of Limerick, Clinical Therapies<br>Motl, Robert; University of Alabama at Birmingham<br>Gallagher, Stephen; University of Limerick, Department of Psychology<br>Larkin, Aidan; Multiple Sclerosis Society of Ireland<br>Newell, John; NUI Galway<br>Scarrott, Carl; University of Canterbury, Mathematics and Statistics<br>Coote, Susan ; University of Limerick, Clinical Therapies; University of Limerick
**Primary Subject Heading**:	Neurology
Secondary Subject Heading:	Rehabilitation medicine
Keywords:	exercise, walking mobility, behaviour change, Multiple sclerosis < NEUROLOGY

SCHOLARONE™
Manuscripts

TITLE PAGE

A randomised controlled pilot trial of an exercise plus behaviour change intervention in people with multiple sclerosis: the Step it Up study

Sara Hayes[1]

Email: sara.hayes@ul.ie

Marcin Kacper Uszynski[1,3]

Email: marcin.uszynski@ul.ie

Robert W. Motl[5]

Email: robmotl@uab.edu

Stephen Gallagher[2]

Email: stephen.gallagher@ul.ie

Aidan Larkin[3]

Email: aidanL@MS-Society.ie

John Newell[4]

Email: john.newell@nuigalway.ie

Carl Scarrott[6]

Email: carl.scarrott@canterbury.ac.nz

*Susan Coote[1]

Email: susan.coote@ul.ie

[1] Department of Clinical Therapies, Faculty of Education and Health Sciences, Health Research Institute, University of Limerick, Limerick, Ireland

[2] Centre for Social Issues Research, Department of Psychology, Faculty of Education and Health Sciences, University of Limerick, Limerick, Ireland

[3] Multiple Sclerosis Society of Ireland, Dublin, Ireland

[4] HRB Clinical Research Facility and School of Mathematics, Statistics and Applied Mathematics, National University of Ireland, Galway, Ireland

For peer review only

For peer review only - http://bmjopen.bmj.com/site/about/guidelines.xhtml

[5] Department of Physical Therapy, School of Health Professions, University of Alabama at Birmingham

[6] HRB Clinical Research Facility, National University of Ireland, Galway, Ireland and School of Mathematics and Statistics, University of Canterbury, New Zealand

*Author for correspondence

For peer review only

For peer review only - http://bmjopen.bmj.com/site/about/guidelines.xhtml

ABSTRACT

Objective: to investigate feasibility of multiple sclerosis (MS) exercise guidelines for inactive people with MS (PwMS) and to examine preliminary efficacy for walking. To investigate effect of augmenting that intervention with education based on Social Cognitive Theory (SCT)

Design: pilot multicentre, double blind, randomised, parallel, controlled trial

Setting: community-delivered programme

Participants: Sixty-five physically inactive PwMS walked independently, scored 0–3 on the Patient Determined Disease Steps Scale, had no MS relapse or change in MS medication in 12 weeks

Interventions: 10-week exercise plus SCT education (SCT) compared with exercise plus attention control education (CON)

Outcome measures: Six Minute Walk Test (6MWT), Timed Up and Go (TUG) test and Multiple Sclerosis Walking Scale-12 (MSWS-12).

Results: 174 expressed interest, 92 were eligible and 65 enrolled (SCT,n=32, CON,n=33). The intervention was feasible and delivered as intended. 68% of SCT group and 50% of control group met the exercise guidelines after intervention.

Using linear mixed effects models, intention to treat basis, there was insufficient evidence for difference between the groups over the trial (6MWT p=0.30, TUG p=0.4, MSWS-12 p=0.8). Using secondary analysis of a cohort with data for ≥3 assessments (SCT n=21, CON n=20), there was significant treatment effect favouring the intervention group (p=0.04) with mean effect for 6MWT 39.0m(95%CI 2.26, 75.73) at 12 weeks and 40.0m(95%CI 2.3, 77.8) at 36 weeks. Both groups improved significantly in 6MWT following 10-week intervention (SCT mean Δ=83.02, sd=60.1, p=<0.01, CON mean Δ=56.92, sd=73.5 p=<0.01), TUG (SCT Δ=-0.70, sd=1.25, p=<0.01, CON Δ=-0.54, sd=0.95, p=<0.01), and MSWS-12 (SCT Δ=-8.03, sd=16.18, p=0.02, CON Δ=-0.86, sd=18.74, p=0.81).

Conclusions: A 10-week exercise programme based on the MS exercise guidelines for improving walking in previously inactive people with MS was feasible. There is marginal evidence of a treatment effect in favour of the exercise plus SCT intervention at 12 and 36 weeks.

For peer review only - http://bmjopen.bmj.com/site/about/guidelines.xhtml

Trial registration NCT02301442.

Funding: Health Research Board, Ireland

Keywords Exercise, walking mobility, Social Cognitive Theory, behaviour change, multiple sclerosis

STRENGTHS AND LIMITATIONS

- New evidence demonstrating the feasibility and preliminary efficacy of delivering a pragmatic, combined, community-based exercise and Social Cognitive Theory education intervention for physically inactive people with MS based on the MS Exercise Guideline
- The use of measures of fidelity, assessments of the target variables of the intervention (strength, fitness and physical activity) and both self-report and objective measures of walking mobility
- Treatment fidelity was considered and evaluated, yet a limitation relates to the use of a 1-day training course for physiotherapists, in particular relating to the novel use of education techniques throughout the exercise programme.
- Attrition of participants between determining eligibility and starting the intervention; the long wait times meant that 29% of eligible participants were lost at this phase.

For peer review only - http://bmjopen.bmj.com/site/about/guidelines.xhtml

INTRODUCTION

Walking limitations are the hallmark of multiple sclerosis (MS)[1] and people with MS report that walking limitations are a significant concern[2]. Indeed, walking limitations have been associated with change in occupation due to MS and occupational disability [3] and influence a range of other outcomes such as cognition and depression[4]. Exercise training remains the cornerstone therapeutic intervention for the management of walking limitations in MS. Many studies report positive effects from a range of exercise interventions as summarised in recent reviews [5] and meta-analyses[6 7] that confirm combined aerobic and resistance exercises can improve both walking speed and walking endurance.

The recent exercise guidelines recommend aerobic exercise twice a week and resistance exercise twice a week as the minimum target for improving walking outcomes among people with mild-to-moderate MS [8]. To that end, we demonstrated using a pragmatic, randomised controlled trial design, that 10 weeks of combined aerobic and resistance training delivered in groups in the community yielded positive improvements in 6MWT [9]. Of concern, however, was that these improvements were not maintained at three-month follow-up[10].

The maintenance of long-term exercise behaviour change is not a problem that is unique to MS, and researchers have highlighted the need for inclusion of behavioural approaches based on theory for long-term behaviour change [11]. Social cognitive theory (SCT) has been most commonly investigated in MS and its domains of exercise self-efficacy and goal setting are consistently associated with physical activity (PA) behaviour [12]. We have reported improvements in PA, and secondary outcomes including walking, from an SCT-based online intervention in MS [13], and one study demonstrated that physical activity behaviour change was maintained three months after cessation of the program[14]. This education program was originally designed based on a RCT of a SCT-based exercise intervention delivered in older adults [15] and later modified and tested for MS [16].

We designed a randomised controlled pilot trial called 'Step it Up' [17] that combined a group exercise programme with a theory-based education component for augmenting the effect of exercise on

For peer review only - http://bmjopen.bmj.com/site/about/guidelines.xhtml

walking outcomes and sustaining these changes over time. The aim of this study was to investigate the feasibility of delivering the combined interventions by physiotherapists and to establish preliminary clinical efficacy for improving walking outcomes; secondary outcomes will be provided in a parallel publication. We delivered the same exercise programme to both groups and controlled for contact by comparing a structured SCT education programme with an attention control education programme, and investigated whether adding the SCT education component would yield greater improvements in walking mobility and whether the improvements were maintained at follow-up. It was hypothesized that that the participants in the exercise and SCT-based intervention would achieve significantly more improvement in walking outcomes than the control group post-intervention and that this improvement would be maintained at follow up. The results of this trial will inform the design, particularly power analysis, of a definitive trial that provides Class 1 evidence (AAN).

METHODS

Study design

This was a multicentre, two-arm, parallel (1:1), double blind, randomised controlled trial.

Setting and participants

The participants were recruited through the MS Society of Ireland, and via neurology clinics in three urban locations in the Republic of Ireland. Details of the recruitment process are further detailed in the protocol paper[17]. Inclusion criteria were: (1) physician-confirmed formal diagnosis of MS, (2) aged 18 years or more, (3) Patient Determined Disease Steps (PDDS) scale score of 0–3, (4) a sedentary lifestyle (<30 minutes of moderate to strenuous exercise one day or more per week over the last six months) and (5) willing to give written informed consent. Exclusion criteria included: (1) pregnancy, (2) MS relapse in the previous 12 weeks and (3) changes to MS medication or steroid

For peer review only - http://bmjopen.bmj.com/site/about/guidelines.xhtml

treatment in the previous 12 weeks. Participants were sent the consent form in advance of the baseline assessment, and written consent was obtained in person.

Randomisation and blinding

Participants were randomly allocated into the exercise plus SCT-based intervention or the exercise plus contact control education intervention. Random allocation procedures have been previously outlined[17] and were adhered to. JN generated the random allocation sequence, the SH enrolled participants, and SC assigned participants to interventions. The outcome assessor (SH) was blind to allocation throughout the study as was the statistician CS during the analysis. All participants were informed that the study aimed to examine the effect of combining exercise and education, and therefore were blinded regarding group allocation.

Screening questionnaire

Potential participants were screened for eligibility for this study using a questionnaire that included the Patient Determined Disease Steps (PDDS) scale[18], confirmation of formal MS diagnosis and questions regarding PA levels. The PDDS scale contains a single item for measuring self-reported neurological impairment on an ordinal level from zero (Normal) to eight (Bedridden). Scores from the PDDS are linearly and strongly related with physician-administered Expanded Disability Status Scale (EDSS) scores[18] .

Outcome measures

Outcome measures were conducted pre-intervention (week 1), post-intervention (week 12), and at 24- and 36-week follow-up.

For peer review only - http://bmjopen.bmj.com/site/about/guidelines.xhtml

Demographic and clinical information

Participants provided details regarding age, gender, level of formal education, time since diagnosis of MS, duration of symptoms of MS, falls history, exercise history, marital status and employment status. Additionally, a researcher formally trained in the use of the Expanded Disability Status Scale (EDSS) (SH) administered the EDSS to all participants at baseline. The EDSS quantifies MS disease progression and is commonly the standard that other outcome measures are compared against [19]. It consists of functional systems subscales and a total score which is an ordinal rating ranging from 0 (normal neurological status) to 10 (death due to MS). MS diagnosis according to the McDonald or Poser criteria was confirmed from the participant's consultant neurologist.

Primary outcomes

The primary outcome was walking mobility at week 36. This was measured using the Six Minute Walk Test (6MWT) as the primary endpoint. The participants were instructed to walk as quickly and as safely as possible for six minutes on a ten meter track. The 6MWT has demonstrated excellent test-retest reliability and concurrent validity among people with mild to moderate MS[20].

We further used the Timed Up and Go test (TUG) and the Multiple Sclerosis Walking Scale-12 (MSWS-12). The TUG has demonstrated excellent test-retest reliability for people with mild MS [21] and the MSWS-12 has demonstrated excellent internal consistency[22 23], test-retest reliability[24] and concurrent validity in people with MS[25].

Adherence

Adherence to the intervention was documented throughout the 10-week intervention via exercise logs. The exercise logs captured attendance at the exercise classes and home exercise sessions. Over the 10-week intervention, 44 total sessions were made available to the participants. This included six exercise classes with strengthening and coaching/education components, four coaching phone calls, 14 prescribed home strengthening sessions, and 20 prescribed home walking sessions.

For peer review only - http://bmjopen.bmj.com/site/about/guidelines.xhtml

For peer review only

We further evaluated adherence to the exercise component by evaluating the effect on strength, fitness and physical activity. The 5 times sit to stand test (5xSTS)[26] (time to complete 5 sit to stand repetitions in seconds) measured lower extremity muscle strength. The Modified Canadian Aerobic Fitness Test (mCAFT)[27] measured fitness and was calculated using following equation; 10 x [17.2 + (1.29 x O2 cost of last stage) – (0.09 x body mass in kg) – (0.18 x Age)]. The Health Index of the Godin Leisure-Time Exercise Questionnaire (GLTEQ)[28] measured PA behaviour. These measures and associated psychometric properties have been described in the trial protocol[17].

Interventions

The content of the interventions delivered in both arms of this RCT has been outlined in detail in the protocol paper[17]. The exercise intervention was common to both groups and was delivered by physiotherapists. The aim of the exercise component was to progressively increase the intensity of both aerobic and strengthening activities to enable the participants to reach the published exercise guidelines for people with mild-to-moderate MS[8], and has been previously described[17]. Over the 10-week programme participants attended the group exercise class at community venues on six occasions, supplemented with a telephone coaching call in the weeks without classes (intervention weeks 4, 6, 7 and 9). After each of the group exercise classes the control group received an education session about topics unrelated to PA behaviour, e.g. diet, vitamin D, sleep, temperature and hydration, and immunisations and vaccinations. The exercise plus SCT-based intervention group received the same exercise intervention as the control group (as described in the previous section). This group also received a similar duration of education based on the principles of SCT for health behaviour change, namely: self-efficacy, outcome expectations, goal-setting, barriers and benefits and has been previously described[17]. The SCT intervention was designed to enable continued exercise behaviour and after the 10-week intervention the participants in both groups received structured phone calls from the intervention physiotherapists at weeks 16, 20 and 36. These telephone calls consisted of direct questions about the frequency, intensity, type and duration of

For peer review only - http://bmjopen.bmj.com/site/about/guidelines.xhtml

exercise participants had completed and whether they had experienced any adverse events or relapses. Additionally the SCT group were coached using the principles of that educational component.

Treatment fidelity

All of the physiotherapists who delivered the intervention or control group sessions were provided with a one-day training course on the delivery of the intervention for their group, directly related to the manual of operating procedures[17]. The intervention was delivered at three sites over the course of the study by 8 physiotherapists broadly representative of those working in primary care. Continued support from the research centre was available if additional training was needed. The fidelity of the physiotherapists' sessions, including both exercise and SCT components, was monitored by randomly allocated video and audio recording of at least one of the intervention sessions. An independent assessor compared the content of the intervention manuals with the video or audio recordings.

Statistical analysis

Sample size

Consistent with data from a large international study[29], it was hypothesised that the effect of the intervention would yield an average improvement in 6MWT distance of 36m with an estimated standard deviation of 48.2m. In order to have 80% power (at the 5% significance level) to detect such a difference in mean improvement in 6MWT over the study period between groups, a sample of size 62 randomised equally to two arms (i.e. 31 per arm) was utilized to inform the target sample size for this pilot study. The intention was to recruit 72 participants to account for drop out and to run the group interventions once sufficient people in that region were eligible. Recruitment in regions was not uniform and participants became ineligible while waiting for others to be recruited.

For peer review only - http://bmjopen.bmj.com/site/about/guidelines.xhtml

Recruitment was better than intended and continued to 92 eligible participants resulting in 65 participants starting the intervention.

Suitable numerical statistics and graphical summaries were used to describe characteristics of the sample at baseline and to assess the validity of any distributional assumptions needed for the formal analysis. All tests of significance were two-sided and conducted at an alpha = 0.05 level of statistical significance. An exploratory paired t-test between baseline and each of the week 12, 24 and 36 follow-ups are conducted, provides a summary of the effects of the estimated treatment and control from the raw data. These “unadjusted” results do not account for the patient covariates and repeated measurements.

The statistical modelling compared differences in the response variables (6MWT, TUG and MSWS scores) between the two intervention arms at each of the three post-intervention follow-ups while correcting for the baseline measurements for each participant. A linear mixed model for a continuous response over time due to the two interventions, whilst adjusting for participant-specific covariates and factors; namely 6MWT at baseline, age, gender, time since diagnosis and MS type (i.e. benign, primary progressive and relapsing-remitting). Treatment and time (and their interaction) were specified as fixed effects, centre (three levels) and subject (nested in centre) as random effects in order to account for homogeneity within centre and within subject correlation over time. Initially a model containing the main effects of the treatment, time and a treatment-by-time interaction was specified in order to test whether there is evidence that the treatment effects varies over time. If the interaction was deemed unnecessary (using a likelihood ratio test) the model was refitted excluding the interaction term, so the treatment effect was then constant over time. Two separate analyses were carried out. Firstly, following an intention-to-treat principle in which all 65 patients who remained eligible to participate were considered. In the secondary analysis, a smaller cohort of 52 patients are analysed, who were identified to have closely adhered to the program by having

For peer review only - http://bmjopen.bmj.com/site/about/guidelines.xhtml

attended at least two of the three follow-ups. All models were fitted in R 3.2.0 using the lme4 and lmerTest packages. Model diagnostics involved suitable plots of the residuals.

RESULTS

Participant sample

One hundred and seventy-four people with MS contacted the trial centre and were screened for inclusion over the phone between September 2013 and May 2014. Eighty-two people were excluded as per the selection criteria (Figure 1) and recruitment ceased when 92 people were randomised to either of the trial arms. Between time of randomisation and initiation of the intervention, 27 eligible participants either became in-eligible or were unable to participate. One participant was not treated as randomised (two acquaintances had been randomised to the other group and they wanted to exercise with them). Sixty-five participants commenced the intervention (SCT group n=33, CON group n=32). In the SCT group, four participants discontinued the intervention and 12 were lost to follow-up at 36-weeks. In the CON group, three participants discontinued the intervention and 10 were lost-to-follow up at 36 weeks. Following the 10-week intervention overall attrition was 17% and at the 36-week follow-up assessment attrition was 34%. Reasons for discontinuing the intervention and loss to follow-up are outlined in Figure 1. Baseline characteristics for both groups are shown in Table 1.

For peer review only - http://bmjopen.bmj.com/site/about/guidelines.xhtml

Table 1 Clinical baseline characteristics in exercise plus SCT group (SCT) and exercise plus education control group (CON)

	SCT (n=33)	CON (n=32)
**MS type**		
Benign	3	1
Primary progressive	1	0
Relapsing-remitting	27	27
Secondary progressive	0	1
Unknown	2	2
**EDSS (median, IQR)**	3.3 (0.7)	3.3 (0.7)
**Years since diagnosis**	6.7 (5.7)	7.0 (6.1)
**Centre (n)**		
Cork	10	9
Galway	8	10
Limerick	15	13
**Age**	43.3 (9.9)	41.9 (9.3)
**Gender (n)**		
Male	4	6
Female	29	26

EDDS: Expanded Disability Disease Scale; IQR: interquartile range; Data given as mean (SD) unless otherwise indicated

Treatment fidelity

An independent person to the intervention (PO'S) used the manual of operating procedures to check if the required content of the programme (both exercise and SCT/attention control education components) was delivered as intended. In both trial arms, 100% of the content of the supervised sessions were implemented as described in the intervention manual.

Feasibility - Exercise Logs

The development of hip pain by one participant in the CON group was the only related adverse event reported by participants in both trial arms during the completion of the 10-week intervention. The SCT and the CON group groups completed an average of 33.2 of 44 available sessions (75.5%) and 32.0 sessions (72.6%), respectively. The proportion of sessions completed is presented in Figure 2, wherein the lowest number of sessions was in week 7 when participants were exercising independently without a class for a second consecutive week. Among the 53 participants who

For peer review only - http://bmjopen.bmj.com/site/about/guidelines.xhtml

provided detailed exercise logs, 17 (68%) of the SCT group and 14 (50%) of the CON group were exercising at the minimum recommended by the exercise guidelines by the end of the 10-week intervention. The reasons for not meeting the guideline included: walking less than 30 minutes twice per week (SCT n=3, CON n=1), walking only once per week (SCT n=2 CON n=5) and doing only one set of each resistance exercise per week (SCT n=2, CON n=4).

In order to further evaluate the adherence to the intervention we investigated the change in strength, fitness and physical activity in order to evaluate whether the intervention changed these intended parameters. Table 2 presents the raw data and unadjusted comparisons. For both groups there were significant improvements in PA and strength from weeks 1 to 12. There was a tendency for aerobic fitness scores to increase, but this change was not statistically significant.

Table 2 Raw data and unadjusted comparisons of change in secondary outcomes from week 1 to week 12 in exercise plus SCT group (SCT) and exercise plus education control group (CON)

		**Week 1 Mean (SD)**	**Week 12 Mean (SD)**	**Mean change from week 1 to week 12 (95% CI)** ***p*-value**
**Godin Health Index**	SCT	3.03 (6.19)	12.48 (11.15)	9.85 (5.46, 14.23) $p<0.01$
	CON	1.88 (4.88)	16.07 (21.12)	12.92 (4.96, 20.89) $p<0.01$
**Five Times Sit to Stand**	SCT	11.48 (2.7)	9.78 (2.18)	-1.51 (-2.42, -0.60) $p<0.01$
	CON	10.8 (2.6)	9.43 (1.93)	-1.55 (-2.30, -0.79) $p<0.01$
**Aerobic Fitness Score**	SCT	295.72 (54.61)	309.12 (53.78)	8.58 (-6.86, 23.98) $p=0.26$
	CON	313.56 (59.02)	331.29 (51.57)	10.54 (-6.29, 27.37) $p=0.21$

CI: confidence interval

For peer review only - http://bmjopen.bmj.com/site/about/guidelines.xhtml

Walking mobility

The mean (SD) scores for the 6MWT, TUG and MSWS-12 at weeks 1, 12, 24 and 36 for participants in the exercise plus SCT and exercise plus education control groups are presented in Table 3. Figure 3 shows the results of the estimated treatment effects on 6MWT, TUG and MSWS-12, as per intention-to-treat and secondary analyses, respectively. The unadjusted, unstandardized mean changes from baseline, and 95% confidence intervals and paired t-test results for both groups are presented in Table 4. Both groups demonstrated an improvement in the primary outcome, 6MWT and secondary outcome MSWS from weeks one to 12 and at 24 and 36 week follow up. For TUG the result are a little more mixed, with evidence of an improvement in both groups from weeks one to 12 which diminishes in the control group by week 36 but a persistent significant difference is observed in the education with SCT group from baseline to weeks 24 and 36.

Table 3 Mean (SD) walking mobility outcomes at weeks 1, 12, 24 and 36 in exercise plus SCT group (SCT) and exercise plus education control group (CON)

	Week 1 mean (SD)		Week 12 mean (SD)		Week 24 mean (SD)		Week 36 mean (SD)	
Intention-to-treat analysis								
**Outcome variable**	**SCT**	**CON**	**SCT**	**CON**	**SCT**	**CON**	**SCT**	**CON**
**6MWT**	445.2 (68.8)	482.0 (72.0)	527.4 (91.1)	547.1 (96.0)	492.8 (73.5)	504.9 (76.9)	515.8 (91.0)	528.0 (93.2)
**TUG**	7.06 (1.61)	6.51 (1.36)	6.27 (1.45)	5.81 (1.08)	6.23 (1.26)	6.00 (0.98)	5.93 (1.33)	5.96 (1.20)
**MSWS-12**	38.0 (28.0)	33.3 (24.8)	29.6 (22.2)	30.8 (21.3)	31.9 (22.1)	26.3 (21.5)	32.6 (23.4)	27.9 (21.9)
Secondary analysis								
	**SCT**	**CON**	**SCT**	**CON**	**SCT**	**CON**	**SCT**	**CON**
**6MWT**	434.6 (65.2)	474.4 (69.6)	524.2 (96.7)	535.2 (88.0)	496.2 (73.7)	504.9 (76.9)	515.8 (91.0)	528.0 (93.2)
**TUG**	7.08 (1.73)	6.65 (1.36)	6.43 (1.46)	5.87 (1.13)	6.30 (1.25)	6.00 (0.98)	5.93 (1.33)	5.96 (1.20)
**MSWS-12**	38.2 (26.7)	31.9 (22.6)	29.7 (22.6)	32.6 (21.0)	31.9 (22.1)	26.3 (21.5)	32.6 (23.4)	27.0 (21.8)

6MWT: Six Minute Walk Test; TUG: Timed Up and Go; MSWS-12: Multiple Sclerosis Walking Scale-12

For peer review only - http://bmjopen.bmj.com/site/about/guidelines.xhtml

Table 4 Unadjusted comparisons of change in walking measures from week 1 to weeks 12, 24 and 36 in exercise plus SCT group (SCT) and exercise plus education control group (CON)

	**Mean change week 1 to week 12 (95% CI) *p*-value**		**Mean change week 1 to week 24 (95% CI) *p*-value**		**Mean change week 1 to week 36 (95% CI) *p*-value**	
	**SCT**	**CON**	**SCT**	**CON**	**SCT**	**CON**
**6MWT**	83.02 (58.74, 107.29) *p*<0.01	56.92 (28.43, 85.41) *p*<0.01	55.97 (32.12, 79.84) *p*<0.01	34.2 (13.43, 54.97) *p*<0.01	82.18 (50.90, 113.45) *p*<0.01	46.87 (18.57, 75.17) *p*<0.01
**TUG**	-0.70 (-1.20, -0.19) *p*<0.01	-0.54 (-0.91, -0.17) *p*<0.01	-0.79 (-1.19, -0.38) *p*<0.01	-0.74 (-1.13, -0.35) *p*<0.01	-1.23 (-1.68, -0.78) *p*<0.01	-0.57 (-0.98, -0.16) *p*<0.01
**MSWS-12**	-8.03 (-14.43, -1.63) *p*=0.02	-0.86 (-7.99, 6.27) *p*=0.81	-6.43 (-12.10, -0.77) *p*=0.03	-2.88 (-11.41, 5.64) *p*=0.49	-8.62 (-15.90, -1.34) *p*=0.02	-5.60 (-13.84, 2.64) *p*=0.17

6MWT: Six Minute Walk Test; TUG: Timed Up and Go; MSWS-12: Multiple Sclerosis Walking Scale-12

The linear mixed models results in Table 5 shows that using an intention-to-treat analysis there was no evidence of a significant treatment effect in favour of the exercise plus SCT compared to the exercise only group for regarding 6MWT, TUG or MSWS scores. Figure 3 confirms the obvious significant effects of the exercise programme found above in the unadjusted paired t-test results, which is shown by the blue and red lines being well above the black "no effect" line when the sample uncertainty conveyed by the corresponding confidence intervals are taken into account. But Figure 3 also confirms lack of evidence for an additional effect of the SCT over the usual exercise programme, which is shown by the widely overlapping confidence intervals between the treatment and control groups.

For peer review only - http://bmjopen.bmj.com/site/about/guidelines.xhtml

Table 5 Estimated treatment effects at weeks 12, 24 and 36 in primary outcome

	Estimate of difference between SCT and Control	Standard error	95% CI	*p*-value
Intention-to-treat analysis				
**6MWT**				
Week 12	22.70	19.00	(-15.14, 60.50)	*0.23*
Week 24	11.80	20.40	(-28.77, 52.36)	*0.56*
Week 36	27.42	20.35	(-13.06, 67.90)	*0.18*
**TUG**				
Week 12	0.069	0.236	(-0.402, 0.541)	*0.77*
Week 24	-0.132	0.250	(-0.630, 0.365)	*0.60*
Week 36	-0.457	0.252	(-0.960, 0.045)	*0.08*
**MSWS-12**				
Week 12	-4.91	4.47	(-13.82, 4.00)	*0.28*
Week 24	-0.59	4.69	(-9.91, 8.73)	*0.90*
Week 36	0.38	4.57	(-8.71, 9.47)	*0.93*
Secondary analysis				
**6MWT**				
Week 12	39.00	18.44	(2.26, 75.73)	*0.04*
Week 24	27.44	19.23	(-10.82, 65.70)	*0.16*
Week 36	40.03	18.97	(2.27, 77.79)	*0.04*
**TUG**				
Week 12	0.204	0.255	(-0.306, 0.713)	*0.43*
Week 24	-0.020	0.261	(-0.542, 0.502)	*0.94*
Week 36	-0.367	0.262	(-0.890, 0.156)	*0.17*
**MSWS-12**				
Week 12	-7.63	4.65	(-16.89, 1.63)	*0.11*
Week 24	-2.50	4.78	(-12.01, 7.02)	*0.60*
Week 36	-1.57	4.69	(-10.93, 7.78)	*0.74*

6MWT: Six Minute Walk Test; TUG: Timed Up and Go; MSWS-12: Multiple Sclerosis Walking Scale-12

A secondary analysis was completed with participants who attended at least two of the three follow-up assessments (SCT n=25, CON n=27). Table 3 presents the mean (SD) scores for the 6MWT, TUG and MSWS-12 at weeks 1, 12, 24 and 36 for participants in the SCT and control groups using secondary analysis. For 6MWT, the SCT group had a marginally more positive outcome, with statistically significant treatment effects evident at weeks 12 and 36 (Table 5). Using this secondary analysis there was no evidence of a treatment effect in favour of the SCT group as compared to the CON group regarding the TUG or MSWS-12 scores.

For peer review only - http://bmjopen.bmj.com/site/about/guidelines.xhtml

DISCUSSION

This pilot RCT investigated the feasibility and preliminary efficacy of the Step it Up programme, a 10-week aerobic and strengthening programme that aimed to enable physically inactive people with MS to exercise according to the recent MS exercise guidelines [8]. We investigated whether embedding an evidence-based exercise programme within a structured SCT-based education programme resulted in improved and more sustained walking outcomes compared to an exercise plus attention control education intervention. To our knowledge, this is the first study to examine the effect of enabling inactive people to meet the minimum recommended dose of the MS exercise guidelines and examine the effects on walking mobility as a primary end-point.

The intervention protocol was feasible and results demonstrated significant improvements in walking mobility following the intervention in both groups. The effect for the SCT group was greater at 12- and 36-week follow-up for the primary outcome, 6MWT, using the secondary analysis which included only patients who adhered to the program (as defined by having attended at least two of the three follow-ups). Recruitment was successful and over nine months at three centres we recruited more than our target of 62 participants (92 eligible participants). The largest point of attrition was while participants waited for enough people to run the group in that region. In the future, recruiting from the largest city in Ireland for a definitive RCT will enable greater numbers to be recruited more quickly and should minimise this attrition at this point in the trial. Retention across the intervention period was good and the attrition rate (17%) was similar to other exercise interventions in people with depression[30] and slightly higher than the average of 15% in a review of exercise trials in MS[31]. While the level of participant attrition in the current programme is greatly improved from our previous community based exercise RCT[9 10], measures such as recruiting a dedicated study coordinator to provide more frequent interactions with participants in the definitive trial will be explored to further enhance retention at follow-up. The addition of booster intervention sessions after the completion of the 10-week intervention will also be explored in the future definitive trial.

For peer review only - http://bmjopen.bmj.com/site/about/guidelines.xhtml

The intervention was delivered by physiotherapists who attended a 1-day training session and treatment fidelity findings suggest that this approach was successful as the interventions were delivered as intended; further training and support may increase the success of the intervention in future. Participants completed on average 73 to 75% of possible sessions suggesting that the protocol is feasible for participants with minimal impairment due to MS. We collected data from exercise logs for demonstrating adherence with the exercise programs. The exercise logs were returned by 82% of participants and used to ascertain whether participants were meeting the MS exercise guideline at the end of the intervention period. It is interesting to note that a greater proportion of participants in the SCT group (68% versus 50%) progressed to meeting the guidelines. Measures to further enhance completion and return of logs (such as offering them in alternative electronic formats) in the future definitive trial are needed.

We further confirmed adherence to this aerobic and strengthening intervention by investigating its effects on strength, fitness and PA. Both 5xSTS and Godin Health index increased significantly and the AFS showed a tendency to improve providing evidence that the exercise intervention met its intended outcomes. Collectively, we believe that the exercise log data combined with fitness and PA outcomes support the successful manipulation of exercise behaviour with in both trial arms. Based on the data on recruitment, retention, feasibility and preliminary efficacy of this group exercise and SCT education intervention we propose to progress to a definitive intervention. To do this, a sample of 49 (for a difference between groups of 39m, assuming a standard deviation of the change score at 36 weeks of 67.85, 80% power, 0.05 significance level) in each group would be needed and we therefore plan to recruit across these three centres again and to add a 4th centre in the largest city in Ireland to minimise attrition.

Importantly, both groups improved significantly in the primary outcome, 6MWT, following the intervention. This improvement in 6MWT is consistent with a recent systematic review of exercise studies that found a significant improvement in walking endurance[7]. We note that the mean

For peer review only - http://bmjopen.bmj.com/site/about/guidelines.xhtml

improvement in the SCT group of 80m and of 60m in the control group far exceeded the value for the clinically important change of 26.1m proposed by Baert et al[29]. Both groups improved more than that reported by Carter et al (2012)[32] in their exercise plus education group, and the magnitude of improvement is more consistent with the improvements noted in a recent community-based intervention among people with moderate-severity MS[33]. We further note that the current physically inactive sample of people with MS with an average age of 42 had 6MWT of 445m at baseline that was less than that of a reference sample aged 70-80 years who walked an average of 514m[34]. This confirms the significant walking impairments for inactive people with mild disability with MS and importantly demonstrates positive improvements due to the Step it Up exercise intervention. Interestingly the SCT group but not the CON group improved in their self-reported walking impairment (MSWS-12) and the magnitude of the change in 6MWT distance may have influenced that finding. Both groups however improved in walking speed and maintained that improvement at 36 week follow-up.

Of note, through the secondary analysis including participants who participated in at least two follow-up assessments, we demonstrated that adding a structured SCT education programme enhanced the effect on 6MWT distance following the 10-week intervention. This is important as it provides information on the preliminary effectiveness of the intervention and confirms the need to augment the retention strategies in the definitive trial. We propose greater training for the interventionists, and greater use of telephone coaching in weeks without classes and between intervention and follow up sessions. Importantly, both the improvement from baseline and the difference in between-group effects were maintained at 36-week follow-up providing new information on the ability to sustain effects after the intervention ceased. Interestingly the effect was reduced at 24 weeks and participants reported that realising they had deteriorated at that assessment served as a prompt to resume their exercise after that assessment. The SCT education programme had six education sessions that targeted outcome expectancies, self-efficacy, goal

For peer review only - http://bmjopen.bmj.com/site/about/guidelines.xhtml

setting, and perceived barriers and benefits of exercise. The components are further consistent with a recent systematic review and meta-analysis of modifiable psychosocial constructs associated with PA in MS that confirmed self-efficacy, goal setting and outcome expectancies as significantly correlated with PA in MS[35]. One novel feature of the current trial is that the SCT education modules were delivered by physiotherapists with minimal training in delivery of behavioural interventions. These findings also support that delivering this SCT education intervention by physiotherapists in a group setting is both feasible and preliminary findings suggest that it may have superior outcomes to an attention control education intervention.

Strengths and Limitations

One of the strengths of this pilot RCT relates to the production of new knowledge around the sustainability of exercise interventions for people with MS. Building on the existing evidence base, we designed and delivered a SCT-based pragmatic physiotherapist-led community exercise. Results demonstrated the feasibility of the protocol among physically-inactive people with mild MS and trends towards clinical efficacy for walking outcomes. The model of care outlined in this pilot study presents as a highly-scalable intervention package for physiotherapists and other healthcare professionals working in primary care services or with 3rd sector organisations (charities). Further study in the form of a definitive trial, including cost and clinical outcomes has the potential to have real policy implications for the provision of rehabilitation to people with MS. Further strengths relate to the use of measures of treatment fidelity, target variables of the intervention (strength, fitness and PA) and both self-report (MSWS) and objective (TUG and 6MWT) measures of walking. Additionally, in the context of an evidence base wherein PA interventions are often not theoretically-based, a key strength of this RCT is the use of the SCT framework to design a behaviour-change intervention; building on the extensive work of the US partner in this trial.

One limitation is the attrition of participants between point of eligibility and allocation to the intervention. The large waiting times resulted in the loss of 29% of eligible participants at this point

For peer review only - http://bmjopen.bmj.com/site/about/guidelines.xhtml

in the trial. Recruitment from larger urban areas with greater numbers of both MS clinics and people with MS is planned for the future definitive trial so that the numbers required to run group classes are met more quickly. A further positive is that we used pedometers and exercise logs to record the intensity and duration of the intervention; however another limitation is that detailed exercise diaries were not returned for all participants. However, a return rate of 82% is acceptable and measures to improve this in the definitive trial will be considered.

Conclusion

This pilot RCT aimed to investigate the feasibility and preliminary efficacy of enabling physically inactive people with MS to meet the MS exercise guidelines [8] through a group exercise and education, physiotherapist-led intervention. We further sought to investigate whether the theory-based SCT component was superior to an attention control education intervention. We found that recruitment was successful, though measures to improve retention in a future definitive trial are needed. Attrition over the intervention and follow-up periods were improved compared to our previous exercise trial [9]. The programme resulted in significant improvements in walking endurance and speed for both groups. While there was no difference in effect between groups at 36 weeks, a secondary analysis of those with data for three of four assessment points demonstrated there was a significant effect in favour of the exercise plus SCT groups compared to the exercise plus control education group at weeks 12 and 36. This supports the preliminary sustained efficacy of the intervention and we propose progressing to a definitive intervention.

Contributors

SH was a post-doctoral researcher on the trial, contributed to the design of the study, collected data, drafted the paper and approved the final version. MU was a post-doctoral researcher on the trial, commented on drafts of the paper and approved the final version. RM co-initiated the project and contributed to the design of the trial, drafted the paper and approved the final version. SG contributed to the design, delivery and evaluation of the trial, commented on drafts of the paper

For peer review only - http://bmjopen.bmj.com/site/about/guidelines.xhtml

For peer review only

and approved the final version. AL contributed to the recruitment strategy employed, commented on drafts of the paper and approved the final version. JN and CS were the statisticians on the trial, cleaned and analysed the data, commented on drafts of the paper and approved the final version. SC was the principal investigator for the study, co-initiated the project, contributed to the design of the trial, drafted the paper and approved the final version.

Funding This work is supported by the Irish Health Research Board Health Research Award, grant number: HRA_PHR/2013-264.

Acknowledgements The authors would like to thank MS Ireland for their assistance with recruitment and running this trial. We would also like to thank Paraic O'Suilleabhain who assisted with data for this paper.

Competing interests All authors have completed the ICMJE uniform disclosure form at www.icmje.org/coi_disclosure.pdf and declare: no support from any organisation for the submitted work; no financial relationships with any organisations that might have an interest in the submitted work in the previous three years; no other relationships or activities that could appear to have influenced the submitted work.

Ethics approval was given by the Faculty of Education and Health Science Research Ethics Committee, University of Limerick (2014_02_20_EHS), in addition to the Research Ethics Committees at the University College Hospital Galway, University Hospital Limerick and Cork University Hospital.

Trial registration NCT02301442

Data sharing statement All data requests pertaining to the Step it Up trial should be made directly to susan.coote@ul.ie

Figure Legends

Figure 1 CONSORT Flow Diagram

Figure 2 Proportion of participants completing sessions (Exercise Diary data).

Figure 3 – Estimated effects using intention to treat and secondary analysis

Appendix 1 – TIDieR checklist

Appendix 2 – Exercise Logs

Appendix 3 - Exercise drawings

REFERENCES

1. Motl RW. Ambulation and Multiple Sclerosis. *Physical Medicine and Rehabilitation Clinics of North America* 2013;24(2):325-36. doi: http://dx.doi.org/10.1016/j.pmr.2012.11.004
2. Heesen C, Bohm J, Reich C, et al. Patient perception of bodily functions in multiple sclerosis: gait and visual function are the most valuable. *Mult Scler* 2008;14(7):988-91.
3. Goldman MD, Motl RW, Scagnelli J, et al. Clinically meaningful performance benchmarks in MS: timed 25-foot walk and the real world. *Neurology* 2013;81(21):1856-63.
4. Learmonth YC, Ensari I, Motl RW. Cognitive motor interference in multiple sclerosis: Insights from a systematic, quantitative review. *Arch Phys Med Rehabil* 2016 doi: 10.1016/j.apmr.2016.07.018 [published Online First: 2016/08/21]
5. Latimer-Cheung AE, Pilutti LA, Hicks AL, et al. Effects of exercise training on fitness, mobility, fatigue, and health-related quality of life among adults with multiple sclerosis: A systematic review to inform guideline development. *Archives Of Physical Medicine And Rehabilitation* 2013;94(9):1800-28. doi: 10.1016/j.apmr.2013.04.020
6. Learmonth YC, Ensari I, Motl RW. Physiotherapy and walking outcomes in adults with multiple sclerosis: systematic review and meta-analysis. *Physical Therapy Reviews* 2016:1-13. doi: 10.1080/10833196.2016.1263415
7. Pearson M, Dieberg G, Smart N. Exercise as a therapy for improvement of walking ability in adults with multiple sclerosis: a meta-analysis. *Arch Phys Med Rehabil* 2015;96(7):1339-48.
8. Latimer-Cheung AE, Martin Ginis KA, Hicks AL, et al. Development of evidence-informed physical activity guidelines for adults with multiple sclerosis. *Arch Phys Med Rehabil* 2013;94(9):1829-36.
9. Garrett M, Hogan N, Larkin A, et al. Exercise in the community for people with minimal gait impairment due to MS: an assessor-blind randomized controlled trial. *Mult Scler* 2013;19(6):782-9.
10. Garrett M, Hogan N, Larkin A, et al. Exercise in the community for people with multiple sclerosis--a follow-up of people with minimal gait impairment. *Mult Scler* 2013;19(6):790-8.
11. Michie S, van Stralen MM, West R. The behaviour change wheel: a new method for characterising and designing behaviour change interventions. *Implementation Science* 2011;6(1):42.
12. Dlugonski D, Wojcicki TR, McAuley E, et al. Social cognitive correlates of physical activity in inactive adults with multiple sclerosis. *Int J Rehabil Res* 2011;34(2):115-20.
13. Motl RW, Dlugonski D, Wojcicki TR, et al. Internet intervention for increasing physical activity in persons with multiple sclerosis. *Mult Scler* 2011;17(1):116-28.
14. Dlugonski D, Motl RW, Mohr DC, et al. Internet-delivered behavioral intervention to increase physical activity in persons with multiple sclerosis: Sustainability and secondary outcomes. *Psychology, Health & Medicine* 2012;17(6):636-51.
15. McAuley E, Courneya Ks Fau - Rudolph DL, Rudolph Dl Fau - Lox CL, et al. Enhancing exercise adherence in middle-aged males and females. (0091-7435 (Print))
16. McAuley E, Motl Rw Fau - Morris KS, Morris Ks Fau - Hu L, et al. Enhancing physical activity adherence and well-being in multiple sclerosis: a randomised controlled trial. (1352-4585 (Print))
17. Coote S, Gallagher S, Msetfi R, et al. A randomised controlled trial of an exercise plus behaviour change intervention in people with multiple sclerosis: the step it up study protocol. *BMC Neurol* 2014;14(241):014-0241.
18. Hadjimichael O, Kerns RD, Rizzo MA, et al. Persistent pain and uncomfortable sensations in persons with multiple sclerosis. *Pain* 2007;127(1-2):35-41.

For peer review only - http://bmjopen.bmj.com/site/about/guidelines.xhtml

19. Goldman MD, Motl RW, Rudick RA. Possible clinical outcome measures for clinical trials in patients with multiple sclerosis. *Ther Adv Neurol Disord* 2010;3(4):229-39. doi: 10.1177/1756285610374117 [published Online First: 2010/12/24]
20. Fry DK, Pfalzer LA. Reliability of four functional tests and rating of perceived exertion in persons with multiple sclerosis. *PHYSIOTHERAPY CANADA* 2006;58(3):212-20.
21. Nilsagard Y, Lundholm C, Gunnarsson L-G, et al. Clinical relevance using timed walk tests and'timed up and go'testing in persons with multiple sclerosis. *Physiotherapy Research International* 2007;12(2):105-14.
22. Hobart J, Riazi A, Lamping D, et al. Measuring the impact of MS on walking ability The 12-Item MS Walking Scale (MSWS-12). *Neurology* 2003;60(1):31-36.
23. McGuigan C, Hutchinson M. The multiple sclerosis impact scale (MSIS-29) is a reliable and sensitive measure. *Journal of neurology, neurosurgery, and psychiatry* 2004;75(2):266-69.
24. Motl RW, McAuley E, Mullen S. Longitudinal measurement invariance of the multiple sclerosis walking scale-12. *Journal Of The Neurological Sciences* 2011;305(1):75-79.
25. Cavanaugh JT, Gappmaier VO, Dibble LE, et al. Ambulatory activity in individuals with multiple sclerosis. *Journal of Neurologic Physical Therapy* 2011;35(1):26-33.
26. Csuka M, McCarty DJ. Simple method for measurement of lower extremity muscle strength. *Am J Med* 1985;78(1):77-81.
27. Kuspinar A, Andersen RE, Teng SY, et al. Predicting exercise capacity through submaximal fitness tests in persons with multiple sclerosis. *Archives Of Physical Medicine And Rehabilitation* 2010;91(9):1410-17.
28. Godin G, Shephard RJ. A simple method to assess exercise behavior in the community. *Can J Appl Sport Sci* 1985;10(3):141-6.
29. Baert I, Freeman J, Smedal T, et al. Responsiveness and clinically meaningful improvement, according to disability level, of five walking measures after rehabilitation in multiple sclerosis a European multicenter study. *Neurorehabilitation and neural repair* 2014:1545968314521010.
30. Stubbs B, Vancampfort D, Rosenbaum S, et al. Dropout from exercise randomized controlled trials among people with depression: A meta-analysis and meta regression. *Journal of affective disorders* 2016;190:457-66.
31. Pilutti LA, Platta ME, Motl RW, et al. The safety of exercise training in multiple sclerosis: A systematic review. *Journal Of The Neurological Sciences* 2014
32. Carter A, Daley A, Humphreys L, et al. Pragmatic intervention for increasing self-directed exercise behaviour and improving important health outcomes in people with multiple sclerosis: a randomised controlled trial. (1477-0970 (Electronic))
33. Learmonth YC, Paul L Fau - Miller L, Miller L Fau - Mattison P, et al. The effects of a 12-week leisure centre-based, group exercise intervention for people moderately affected with multiple sclerosis: a randomized controlled pilot study. (1477-0873 (Electronic))
34. Casanova C, Celli B, Barria P, et al. The 6-min walk distance in healthy subjects: reference standards from seven countries. *European Respiratory Journal* 2011;37(1):150-56.
35. Casey B, Coote S, Shirazipour C, et al. Modifiable Psychosocial Constructs Associated with Physical Activity Participation in People with Multiple Sclerosis: A Systematic Review and Meta-Analysis. *Archives of Physical Medicine and Rehabilitation* 2017;in press

For peer review only - http://bmjopen.bmj.com/site/about/guidelines.xhtml

Figure 1 *CONSORT Flow Diagram,*

DNA: did not attend

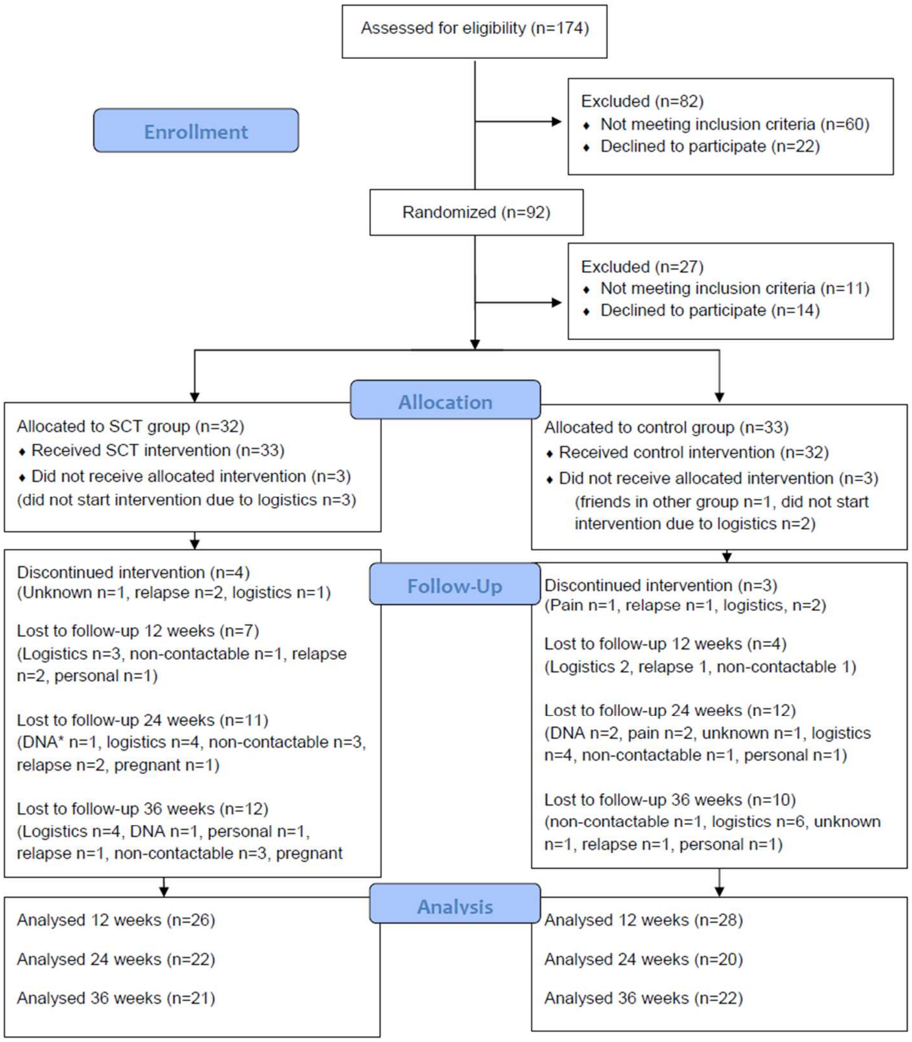

Figure 1 CONSORT Flow Diagram

331x426mm (72 x 72 DPI)

Figure 2 Proportion of participants completing sessions (Exercise Diary data).

SCT = exercise plus social cognitive theory education group, CON = exercise plus contact control education

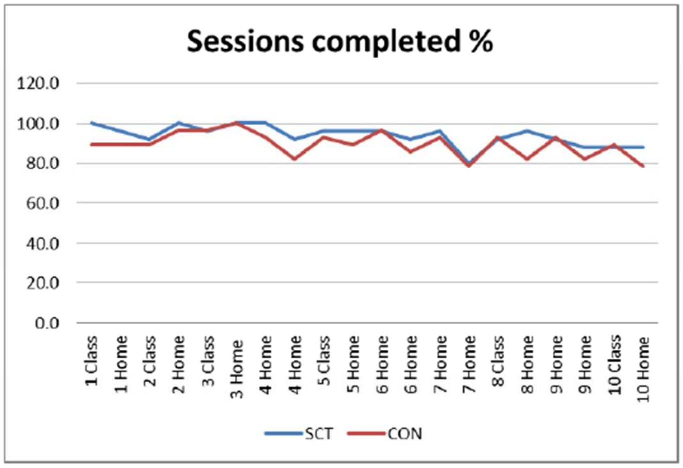

Figure 2 Proportion of participants completing sessions (Exercise Diary data).

277x171mm (72 x 72 DPI)

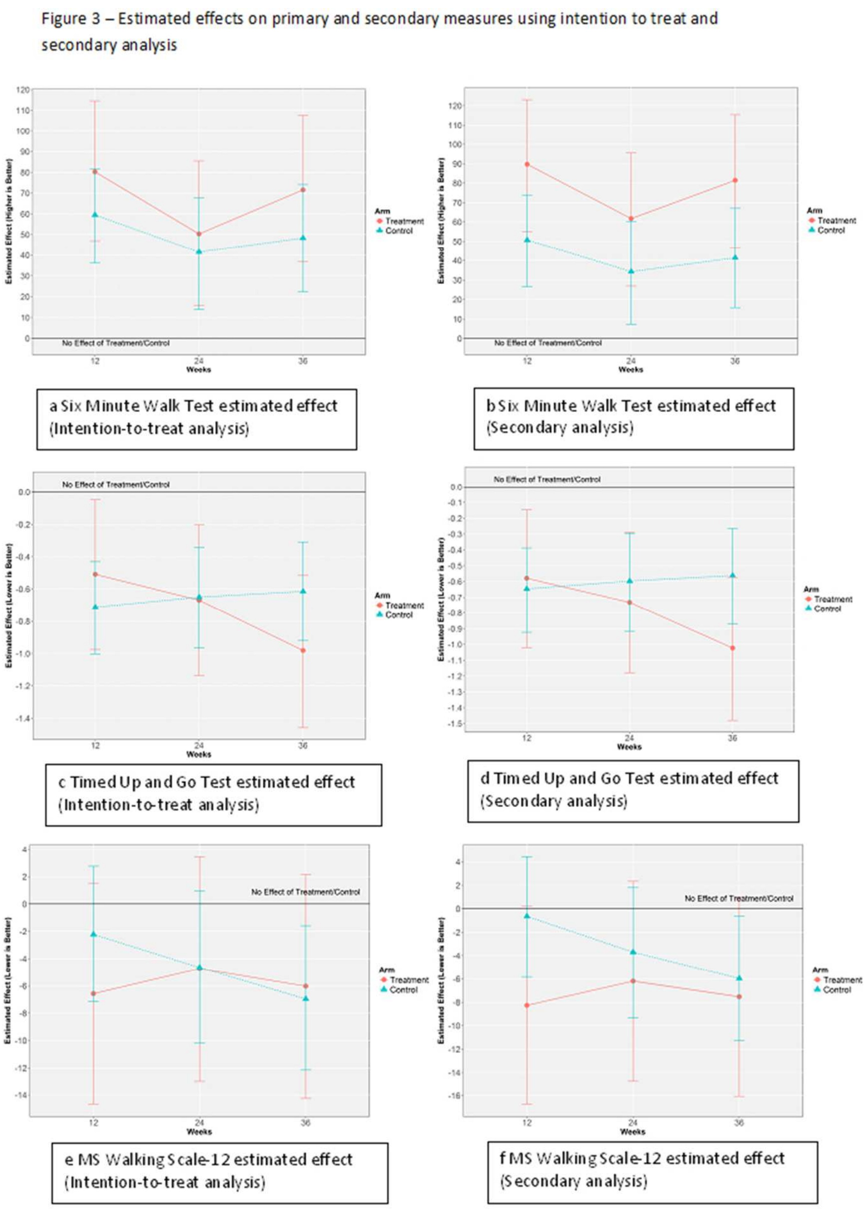

Figure 3 – Estimated effects using intention to treat and secondary analysis

244x337mm (72 x 72 DPI)

The TIDieR (Template for Intervention Description and Replication) Checklist*:

Information to include when describing an intervention and the location of the information

Item number	Item	Where located **	
		Primary paper (page or appendix number)	Other † (details)
	**BRIEF NAME**		
**1.**	Provide the name or a phrase that describes the intervention.	1	Title; Exercise plus behaviour change intervention
	**WHY**		
**2.**	Describe any rationale, theory, or goal of the elements essential to the intervention.	5,6	We designed a randomised controlled pilot trial called 'Step it Up' that combined a group exercise programme with a theory-based education component for augmenting the effect of exercise on walking outcomes and sustaining these changes over time. We compared SCT based education to attention control education on topics unrelated to exercise. SCT was used to develop the content of the educational element as it has been widely investigated and associated with PA behaviour in people with MS
	**WHAT**		
**3.**	Materials: Describe any physical or informational materials used in the intervention, including those provided to participants or used in intervention delivery or in training of intervention providers. Provide information on where the	9<br>Protocol paper page 3, 4<br>https://bmcneu	The exercise log book and exercise pictures are available as an online appendix

TIDieR checklist

For peer review only - http://bmjopen.bmj.com/site/about/guidelines.xhtml

	materials can be accessed (e.g. online appendix, URL).	rol.biomedcentral.com/articles/10.1186/s12883-014-0241-9	
**4.**	Procedures: Describe each of the procedures, activities, and/or processes used in the intervention, including any enabling or support activities.	9<br>Protocol paper page 3	Over the 10-week programme participants attended the group exercise class on six occasions, supplemented with a telephone coaching call in the weeks without classes (intervention weeks 4, 6, 7 and 9). After each of the group exercise classes both groups received an education session and the content is described in the protocol paper
	**WHO PROVIDED**		
**5.**	For each category of intervention provider (e.g. psychologist, nursing assistant), describe their expertise, background and any specific training given.	10	The physiotherapists who delivered the intervention or control group sessions were provided with a one-day training course on the delivery of the intervention for their group.
	**HOW**		
**6.**	Describe the modes of delivery (e.g. face-to-face or by some other mechanism, such as internet or telephone) of the intervention and whether it was provided individually or in a group.	9	There were 44 sessions over 10 weeks; 6 group strengthening classes followed by education, 14 home strengthening classes, 20 home walking sessions and 4 telephone calls
	**WHERE**		
**7.**	Describe the type(s) of location(s) where the intervention occurred, including any necessary infrastructure or relevant features.	9	Recruitment and interventions took place in Cork Galway and Limerick Ireland. All classes happened at community venues, the other sessions were home based.
	**WHEN and HOW MUCH**		
**8.**	Describe the number of times the intervention was delivered	See 6 above	The target walking exercise intensity

For peer review only - http://bmjopen.bmj.com/site/about/guidelines.xhtml

	and over what period of time including the number of sessions, their schedule, and their duration, intensity or dose.	Protocol paper page 3 describes intensity of waling and strengthening sessions.	for both groups in the current study was at a rate of 100 steps per minute. Participants started with 10 minutes of walking twice weekly at a rate of 100 steps/minute and increased incrementally in 5 minute intervals over 5 weeks wherein they aimed to reach the guideline of30 minutes twice weekly<br>The intensity and duration of the strengthening component of the intervention was progressed by increasing the number of repetitions and sets and changing the resistance of the elastic resistance band used for each strengthening exercise. Participants started with one set of 10–15 repetitions and gradually increased the number of sets, repetitions and level of resistance until they meet the target of two sets of each exercise twice weekly with sufficient resistance that they are failing on the 12th repetition.
	**TAILORING**		
**9.**	If the intervention was planned to be personalised, titrated or adapted, then describe what, why, when, and how.	Protocol paper page 3	Intensity was personalised based on each participants ability/performance of resistance and aerobic exercise Progression through the programme was based on individual performance in the previous session.
	**MODIFICATIONS**		
**10.**‡	If the intervention was modified during the course of the study, describe the changes (what, why, when, and how).	13, 14	Not all participants met the guideline target by week 6. The proportion of participants in each group reaching the guideline and reasons for not reaching guideline are described in the results

TIDieR checklist

For peer review only

	**HOW WELL**		
**11.**	Planned: If intervention adherence or fidelity was assessed, describe how and by whom, and if any strategies were used to maintain or improve fidelity, describe them.	12, Protocol paper page 4	Exercise logs, video or/audio recording of sessions and independent evaluation of those recorded sessions were utilised to evaluate fidelity
**12.‡**	Actual: If intervention adherence or fidelity was assessed, describe the extent to which the intervention was delivered as planned.	13,14	Adherence to the programme evaluated using the exercise logs<br>Fidelity was assessed by an independent person using the video/audio recordings

** **Authors** - use N/A if an item is not applicable for the intervention being described. **Reviewers** – use '?' if information about the element is not reported/not sufficiently reported.

† If the information is not provided in the primary paper, give details of where this information is available. This may include locations such as a published protocol or other published papers (provide citation details) or a website (provide the URL).

‡ If completing the TIDieR checklist for a protocol, these items are not relevant to the protocol and cannot be described until the study is complete.

* We strongly recommend using this checklist in conjunction with the TIDieR guide (see *BMJ* 2014;348:g1687) which contains an explanation and elaboration for each item.

* The focus of TIDieR is on reporting details of the intervention elements (and where relevant, comparison elements) of a study. Other elements and methodological features of studies are covered by other reporting statements and checklists and have not been duplicated as part of the TIDieR checklist. When a **randomised trial** is being reported, the TIDieR checklist should be used in conjunction with the CONSORT statement (see www.consort-statement.org) as an extension of **Item 5 of the CONSORT 2010 Statement.** When a **clinical trial protocol** is being reported, the TIDieR checklist should be used in conjunction with the SPIRIT statement as an extension of **Item 11 of the SPIRIT 2013 Statement** (see www.spirit-statement.org). For alternate study designs, TIDieR can be used in conjunction with the appropriate checklist for that study design (see www.equator-network.org).

For peer review only - http://bmjopen.bmj.com/site/about/guidelines.xhtml

For peer review only

Your Step it Up exercise Log book

Please use this log book to record **BOTH** the strengthening exercises and the walking exercise that you do throughout this programme. This log book is to be used **both during your exercise classes** with your physiotherapist and **while you are doing your exercises at home**. The reason we ask you to complete this log is so you can keep a reliable record of progress that you are making with the Step it Up programme. It is also important for the research team at UL to track your progress with the programme.

Each week you should fill the relevant table. As you can see, in the last 2 columns of each of the tables- we have asked you to rate your "BORG score" and your "enjoyment score"- at the end of this document you will see instructions on how to complete these ratings.

If you have any questions regarding filling this log book out please do not hesitate to contact Dr. Susan Coote (susan.coote@ul.ie) or the physiotherapist who is delivering your programme. When the 10-week exercise class is finished, please give your log book to the physiotherapist who completes your follow-up assessment (Dr. Sara Hayes). You will be given a date for this assessment closer to the time.

© University of Limerick/University of Illinois, Urbana-Champaign 2014. This information is being provided solely for the purpose of delivering this intervention as part of the HRB funded Step it Up Trial and should not to be reproduced without permission or used for any other purpose.

For peer review only - http://bmjopen.bmj.com/site/about/guidelines.xhtml

Week 1

Date	Strengthening exercises										BORG score (6-20)	Enjoyment score (1-7)
Class	Knee bend	Squat	Shoulder bend-forward	Shoulder bend-sideways	Elbow bend	Lunge	Hip bend-backward	Hip bend-sideways	Heel raises-standing	Ankle bend-sitting		
	Reps	Reps	Reps	Reps	Reps	Reps	Reps	Reps	Reps	Reps		
	Sets	Sets	Sets	Sets	Sets	Sets	Sets	Sets	Sets	Sets		
	Colour	Colour	Colour	Colour	Colour	Colour	Colour	Colour	Colour	Colour		
Home	Knee bend	Squat	Shoulder bend-forward	Shoulder bends-sideways	Elbow bend	Lunge	Hip bend-backward	Hip bends-sideways	Heel raises-standing	Ankle bend-sitting		
	Reps	Reps	Reps	Reps	Reps	Reps	Reps	Reps	Reps	Reps		
	Sets	Sets	Sets	Sets	Sets	Sets	Sets	Sets	Sets	Sets		
	Colour	Colour	Colour	Colour	Colour	Colour	Colour	Colour	Colour	Colour		
	Walking exercise											
Home	Time spent walking (minutes):				Number of steps:							
Home	Time spent walking (minutes):				Number of steps:							

For peer review only

Week 2

Date	Strengthening exercises										BORG score (6-20)	Enjoyment score (1-7)
**Class**	**Knee bend**	**Squat**	**Shoulder bend-forward**	**Shoulder bends-sideways**	**Elbow bend**	**Lunge**	**Hip bend-backward**	**Hip bend-sideways**	**Heel raises-standing**	**Ankle bend-sitting**		
	Reps	Reps	Reps	Reps	Reps	Reps	Reps	Reps	Reps	Reps		
	Sets	Sets	Sets	Sets	Sets	Sets	Sets	Sets	Sets	Sets		
	Colour	Colour	Colour	Colour	Colour	Colour	Colour	Colour	Colour	Colour		
**Home**	**Knee bend**	**Squat**	**Shoulder bend-forward**	**Shoulder bends-sideways**	**Elbow bend**	**Lunge**	**Hip bend-backward**	**Hip bends-sideways**	**Heel raises-standing**	**Ankle bend-sitting**		
	Reps	Reps	Reps	Reps	Reps	Reps	Reps	Reps	Reps	Reps		
	Sets	Sets	Sets	Sets	Sets	Sets	Sets	Sets	Sets	Sets		
	Colour	Colour	Colour	Colour	Colour	Colour	Colour	Colour	Colour	Colour		
	**Walking exercise**											
**Home**	**Time spent walking (minutes):**				**Number of steps:**							
**Home**	**Time spent walking (minutes):**				**Number of steps:**							

For peer review only

Week 3

Date	Strengthening exercises										BORG score (6-20)	Enjoyment score (1-7)
**Class**	**Knee bend**	**Squat**	**Shoulder bend-forward**	**Shoulder bends-sideways**	**Elbow bend**	**Lunge**	**Hip bend-backward**	**Hip bend-sideways**	**Heel raises-standing**	**Ankle bend-sitting**		
	Reps	Reps	Reps	Reps	Reps	Reps	Reps	Reps	Reps	Reps		
	Sets	Sets	Sets	Sets	Sets	Sets	Sets	Sets	Sets	Sets		
	Colour	Colour	Colour	Colour	Colour	Colour	Colour	Colour	Colour	Colour		
**Home**	**Knee bend**	**Squat**	**Shoulder bend-forward**	**Shoulder bends-sideways**	**Elbow bend**	**Lunge**	**Hip bend-backward**	**Hip bend-sideways**	**Heel raises-standing**	**Ankle bend-sitting**		
	Reps	Reps	Reps	Reps	Reps	Reps	Reps	Reps	Reps	Reps		
	Sets	Sets	Sets	Sets	Sets	Sets	Sets	Sets	Sets	Sets		
	Colour	Colour	Colour	Colour	Colour	Colour	Colour	Colour	Colour	Colour		
	**Walking exercise**											
**Home**	**Time spent walking (minutes):**				**Number of steps:**							
**Home**	**Time spent walking (minutes):**				**Number of steps:**							

For peer review only

Week 4

Date	Strengthening exercises										BORG score (6-20)	Enjoyment score (1-7)
Home	**Knee bend**	**Squat**	**Shoulder bend-forward**	**Shoulder bends-sideways**	**Elbow bend**	**Lunge**	**Hip bend-backward**	**Hip bends-sideways**	**Heel raises-standing**	**Ankle bend-sitting**		
	Reps	Reps	Reps	Reps	Reps	Reps	Reps	Reps	Reps	Reps		
	Sets	Sets	Sets	Sets	Sets	Sets	Sets	Sets	Sets	Sets		
	Colour	Colour	Colour	Colour	Colour	Colour	Colour	Colour	Colour	Colour		
Home	**Knee bend**	**Squat**	**Shoulder bend-forward**	**Shoulder bends-sideways**	**Elbow bend**	**Lunge**	**Hip bend-backward**	**Hip bends-sideways**	**Heel raises-standing**	**Ankle bend-sitting**		
	Reps	Reps	Reps	Reps	Reps	Reps	Reps	Reps	Reps	Reps		
	Sets	Sets	Sets	Sets	Sets	Sets	Sets	Sets	Sets	Sets		
	Colour	Colour	Colour	Colour	Colour	Colour	Colour	Colour	Colour	Colour		
	**Walking exercise**											
Home	**Time spent walking (minutes):**				**Number of steps:**							
Home	**Time spent walking (minutes):**				**Number of steps:**							

For peer review only

Week 5

Date	Strengthening exercises										BORG score (6-20)	Enjoyment score (1-7)
Class	Knee bend	Squat	Shoulder bend-forward	Shoulder bends-sideways	Elbow bend	Lunge	Hip bend-backward	Hip bends-sideways	Heel raises-standing	Ankle bend-sitting		
	Reps	Reps	Reps	Reps	Reps	Reps	Reps	Reps	Reps	Reps		
	Sets	Sets	Sets	Sets	Sets	Sets	Sets	Sets	Sets	Sets		
	Colour	Colour	Colour	Colour	Colour	Colour	Colour	Colour	Colour	Colour		
Home	Knee bend	Squat	Shoulder bend-forward	Shoulder bends-sideways	Elbow bend	Lunge	Hip bend-backward	Hip bend-sideways	Heel raises-standing	Ankle bend-sitting		
	Reps	Reps	Reps	Reps	Reps	Reps	Reps	Reps	Reps	Reps		
	Sets	Sets	Sets	Sets	Sets	Sets	Sets	Sets	Sets	Sets		
	Colour	Colour	Colour	Colour	Colour	Colour	Colour	Colour	Colour	Colour		
	Walking exercise											
Home	Time spent walking (minutes):				Number of steps:							
Home	Time spent walking (minutes):				Number of steps:							

For peer review only

Week 6

Date	Strengthening exercises										BORG score (6-20)	Enjoyment score (1-7)
Home	**Knee bend**	**Squat**	**Shoulder bend-forward**	**Shoulder bends-sideways**	**Elbow bend**	**Lunge**	**Hip bend-backward**	**Hip bend-sideways**	**Heel raises-standing**	**Ankle bend-sitting**		
	Reps	Reps	Reps	Reps	Reps	Reps	Reps	Reps	Reps	Reps		
	Sets	Sets	Sets	Sets	Sets	Sets	Sets	Sets	Sets	Sets		
	Colour	Colour	Colour	Colour	Colour	Colour	Colour	Colour	Colour	Colour		
Home	**Knee bend**	**Squat**	**Shoulder bend-forward**	**Shoulder bends-sideways**	**Elbow bend**	**Lunge**	**Hip bend-backward**	**Hip bend-sideways**	**Heel raises-standing**	**Ankle bend-sitting**		
	Reps	Reps	Reps	Reps	Reps	Reps	Reps	Reps	Reps	Reps		
	Sets	Sets	Sets	Sets	Sets	Sets	Sets	Sets	Sets	Sets		
	Colour	Colour	Colour	Colour	Colour	Colour	Colour	Colour	Colour	Colour		
	**Walking exercise**											
**Home**	**Time spent walking (minutes):**				**Number of steps:**							
**Home**	**Time spent walking (minutes):**				**Number of steps:**							

For peer review only

Week 7

Date	Strengthening exercises										BORG score (6-20)	Enjoyment score (1-7)
Home	Knee bend	Squat	Shoulder bend-forward	Shoulder bends-sideways	Elbow bend	Lunge	Hip bend-backward	Hip bend-sideways	Heel raises-standing	Ankle bend-sitting		
	Reps	Reps	Reps	Reps	Reps	Reps	Reps	Reps	Reps	Reps		
	Sets	Sets	Sets	Sets	Sets	Sets	Sets	Sets	Sets	Sets		
	Colour	Colour	Colour	Colour	Colour	Colour	Colour	Colour	Colour	Colour		
Home	Knee bend	Squat	Shoulder bend-forward	Shoulder bends-sideways	Elbow bend	Lunge	Hip bend-backward	Hip bend-sideways	Heel raises-standing	Ankle bend-sitting		
	Reps	Reps	Reps	Reps	Reps	Reps	Reps	Reps	Reps	Reps		
	Sets	Sets	Sets	Sets	Sets	Sets	Sets	Sets	Sets	Sets		
	Colour	Colour	Colour	Colour	Colour	Colour	Colour	Colour	Colour	Colour		
	**Walking exercise**											
Home	Time spent walking (minutes):				Number of steps:							
Home	Time spent walking (minutes):				Number of steps:							

For peer review only

Week 8

Date	Strengthening exercises										BORG score (6-20)	Enjoyment score (1-7)
Class	Knee bend	Squat	Shoulder bend-forward	Shoulder bends-sideways	Elbow bend	Lunge	Hip bend-backward	Hip bend-sideways	Heel raises-standing	Ankle bend-sitting		
	Reps	Reps	Reps	Reps	Reps	Reps	Reps	Reps	Reps	Reps		
	Sets	Sets	Sets	Sets	Sets	Sets	Sets	Sets	Sets	Sets		
	Colour	Colour	Colour	Colour	Colour	Colour	Colour	Colour	Colour	Colour		
Home	Knee bend	Squat	Shoulder bend-forward	Shoulder bends-sideways	Elbow bend	Lunge	Hip bend-backward	Hip bend-sideways	Heel raises-standing	Ankle bend-sitting		
	Reps	Reps	Reps	Reps	Reps	Reps	Reps	Reps	Reps	Reps		
	Sets	Sets	Sets	Sets	Sets	Sets	Sets	Sets	Sets	Sets		
	Colour	Colour	Colour	Colour	Colour	Colour	Colour	Colour	Colour	Colour		
	Walking exercise											
Home	Time spent walking (minutes):				Number of steps:							
Home	Time spent walking (minutes):				Number of steps:							

Week 9

Date	Strengthening exercises										BORG score (6-20)	Enjoyment score (1-7)
**Home**	**Knee bend**	**Squat**	**Shoulder bend-forward**	**Shoulder bends-sideways**	**Elbow bend**	**Lunge**	**Hip bend-backward**	**Hip bend-sideways**	**Heel raises-standing**	**Ankle bend-sitting**		
	Reps	Reps	Reps	Reps	Reps	Reps	Reps	Reps	Reps	Reps		
	Sets	Sets	Sets	Sets	Sets	Sets	Sets	Sets	Sets	Sets		
	Colour	Colour	Colour	Colour	Colour	Colour	Colour	Colour	Colour	Colour		
**Home**	**Knee bend**	**Squat**	**Shoulder bend-forward**	**Shoulder bends-sideways**	**Elbow bend**	**Lunge**	**Hip bend-backward**	**Hip bend-sideways**	**Heel raises-standing**	**Ankle bend-sitting**		
	Reps	Reps	Reps	Reps	Reps	Reps	Reps	Reps	Reps	Reps		
	Sets	Sets	Sets	Sets	Sets	Sets	Sets	Sets	Sets	Sets		
	Colour	Colour	Colour	Colour	Colour	Colour	Colour	Colour	Colour	Colour		
	**Walking exercise**											
**Home**	**Time spent walking (minutes):**				**Number of steps:**							
**Home**	**Time spent walking (minutes):**				**Number of steps:**							

For peer review only

Week 10

Date	Strengthening exercises										BORG score (6-20)	Enjoyment score (1-7)
**Class**	**Knee bend**	**Squat**	**Shoulder bend-forward**	**Shoulder bends-sideways**	**Elbow bend**	**Lunge**	**Hip bend-backward**	**Hip bend-sideways**	**Heel raises-standing**	**Ankle bend-sitting**		
	Reps	Reps	Reps	Reps	Reps	Reps	Reps	Reps	Reps	Reps		
	Sets	Sets	Sets	Sets	Sets	Sets	Sets	Sets	Sets	Sets		
	Colour	Colour	Colour	Colour	Colour	Colour	Colour	Colour	Colour	Colour		
**Home**	**Knee bend**	**Squat**	**Shoulder bend-forward**	**Shoulder bends-sideways**	**Elbow bend**	**Lunge**	**Hip bend-backward**	**Hip bend-sideways**	**Heel raises-standing**	**Ankle bend-sitting**		
	Reps	Reps	Reps	Reps	Reps	Reps	Reps	Reps	Reps	Reps		
	Sets	Sets	Sets	Sets	Sets	Sets	Sets	Sets	Sets	Sets		
	Colour	Colour	Colour	Colour	Colour	Colour	Colour	Colour	Colour	Colour		
	**Walking exercise**											
**Home**	**Time spent walking (minutes):**				**Number of steps:**							
**Home**	**Time spent walking (minutes):**				**Number of steps:**							

For peer review only

***BORG scoring instructions**

While doing physical activity, we want you to rate your perception of exertion. This feeling should reflect how heavy and strenuous the exercise feels to you, Combining all sensations and feelings of physical stress, effort, and fatigue. Do not concern yourself with any one factor such as leg pain or shortness of breath, but try to focus on your total feeling of exertion.

Look at the rating scale below while you are engaging in an activity; it ranges from 6 to 20, where 6 means "no exertion at all" and 20 means "maximal exertion." Choose the number from below that best describes your level of exertion. This will give you a good idea of the intensity level of your activity, and you can use this information to speed up or slow down your movements to reach your desired range.

Try to appraise your feeling of exertion as honestly as possible, without thinking about what the actual physical load is. Your own feeling of effort and exertion is important, not how it compares to other people's. Look at the scales and the expressions and then give a number.

---

6 No exertion at all

7 Extremely light

8

9 Very light - (easy walking slowly at a comfortable pace)

10

11 Light

12

13 Somewhat hard (It is quite an effort; you feel tired but can continue)

14

15 Hard (heavy)

16

17 Very hard (very strenuous, and you are very fatigued)

18

19 Extremely hard (You cannot continue for long at this pace)

20 Maximal exertion

© University of Limerick/University of Illinois, Urbana-Champaign 2014. This information is being provided solely for the purpose of delivering this intervention as part of the HRB funded Step it Trial and should not to be reproduced without permission or used for any other purpose.

For peer review only

** Your Enjoyment Scale Score

Enjoyment Scale<br>"How much did you enjoy your exercise session today?"						
1	2	3	4	5	6	7
not at all			somewhat			very much

For peer review only

© University of Limerick/University of Illinois, Urbana-Champaign 2014. This information is being provided solely for the purpose of delivering this intervention as part of the HRB funded Step it Trial and should not to be reproduced without permission or used for any other purpose.

For peer review only

For peer review only - http://bmjopen.bmj.com/site/about/guidelines.xhtml

Knee bend

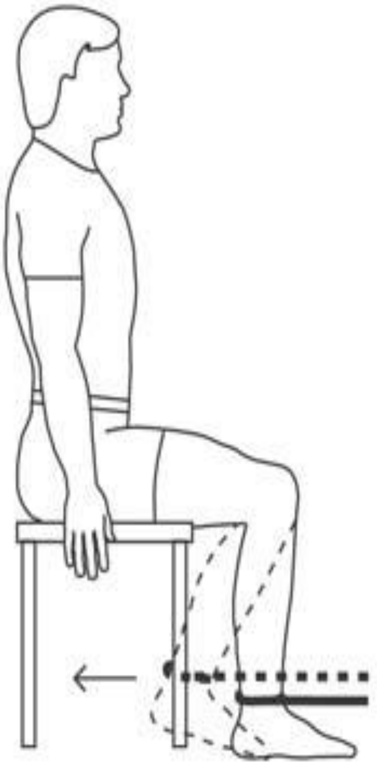

Squat

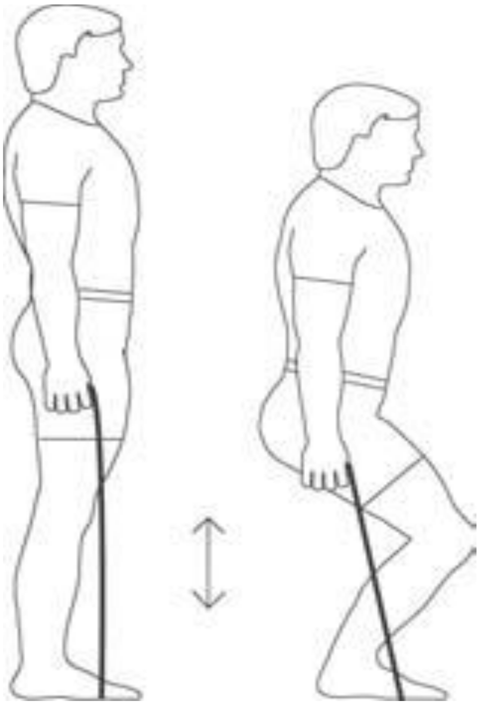

For peer review only

Shoulder bend- forward

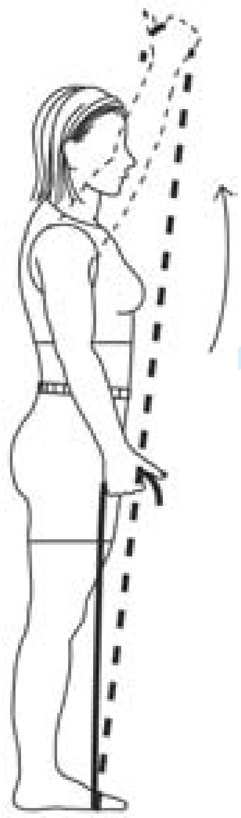

Shoulder bend-sideways

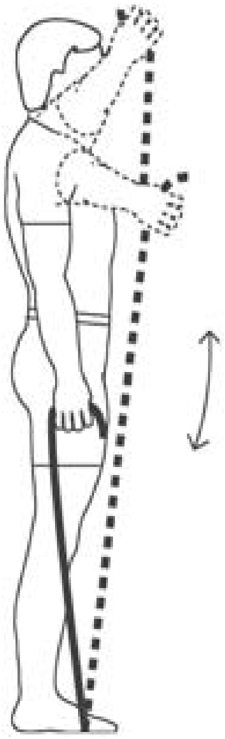

For peer review only

Elbow bend

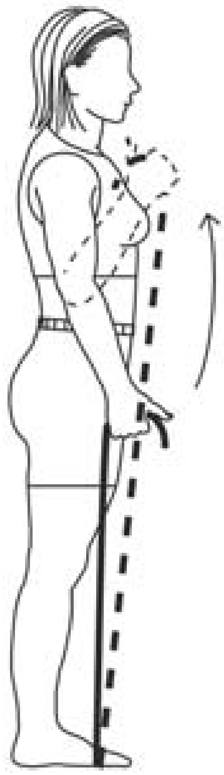

Lunge

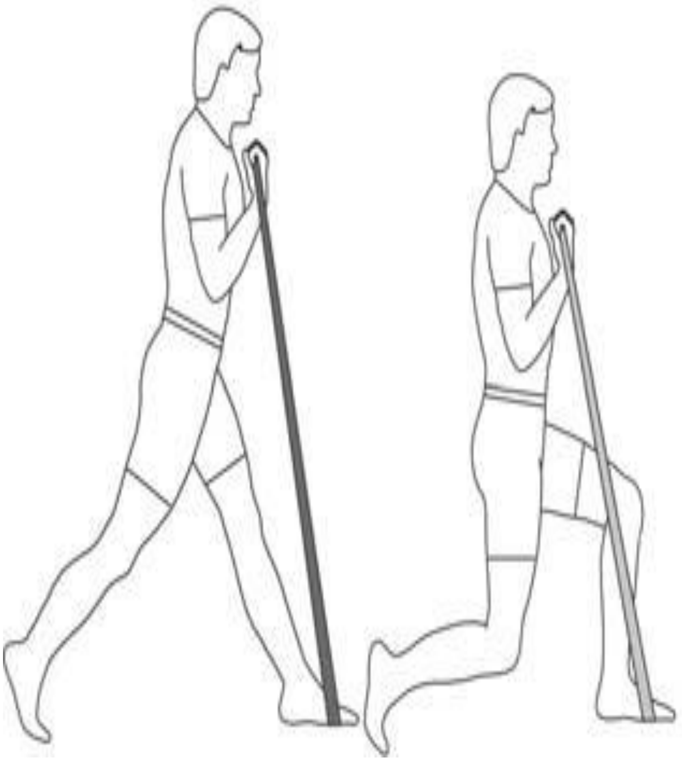

For peer review only

Hip bend- backward

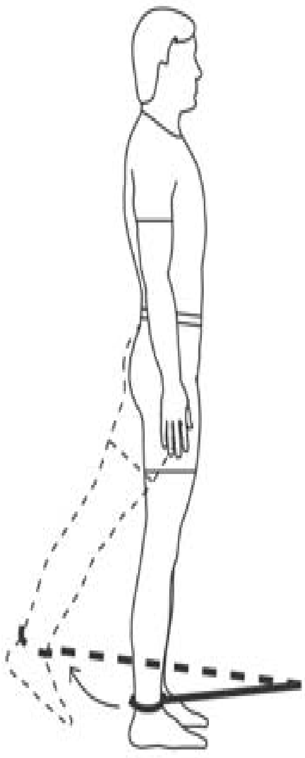

Hip bend- sideways

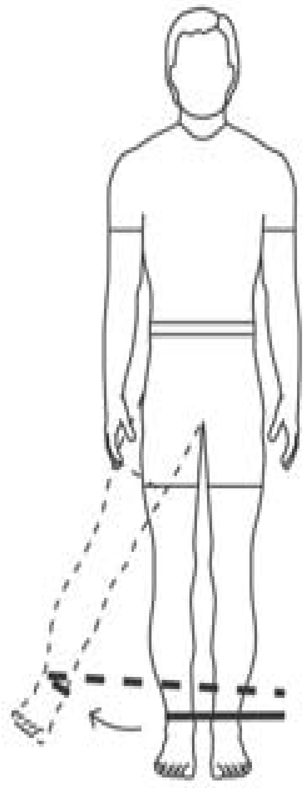

Heel raises- standing

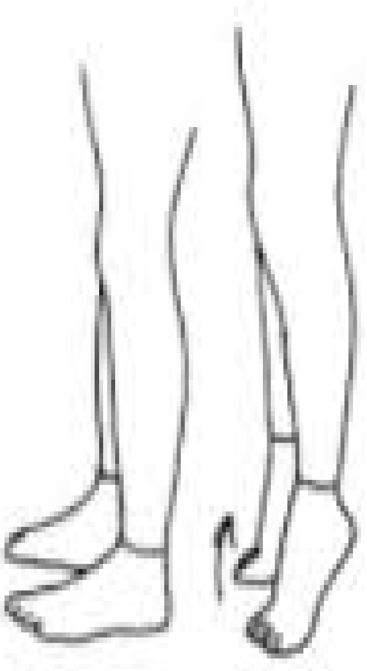

Ankle bend- sitting

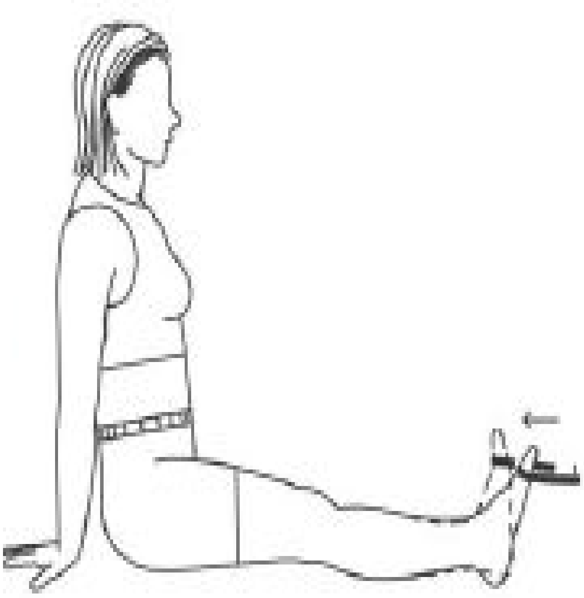

For peer review only

CONSORT checklist of information to include when reporting a pilot trial*

Section/topic and item No	Standard checklist item	Extension for pilot trials	Page No where item is reported
**Title and abstract**			
1a	Identification as a randomised trial in the title	Identification as a pilot or feasibility randomised trial in the title	1
1b	Structured summary of trial design, methods, results, and conclusions (for specific guidance see CONSORT for abstracts)	Structured summary of pilot trial design, methods, results, and conclusions (for specific guidance see CONSORT abstract extension for pilot trials)	3
**Introduction**			
Background and objectives:			
2a	Scientific background and explanation of rationale	Scientific background and explanation of rationale for future definitive trial, and reasons for randomised pilot trial	5
2b	Specific objectives or hypotheses	Specific objectives or research questions for pilot trial	5-6
**Methods**			
Trial design:			
3a	Description of trial design (such as parallel, factorial) including allocation ratio	Description of pilot trial design (such as parallel, factorial) including allocation ratio	6
3b	Important changes to methods after trial commencement (such as eligibility criteria), with reasons	Important changes to methods after pilot trial commencement (such as eligibility criteria), with reasons	10
Participants:			
4a	Eligibility criteria for participants		
4b	Settings and locations where the data were collected		
4c		How participants were identified and consented	6
Interventions:			
5	The interventions for each group with sufficient details to allow replication, including how and when they were actually administered		
Outcomes:			
6a	Completely defined prespecified primary and secondary outcome measures, including how and when they were assessed	Completely defined prespecified assessments or measurements to address each pilot trial objective specified in 2b, including how and when they were assessed	7
6b	Any changes to trial outcomes after the trial commenced, with reasons	Any changes to pilot trial assessments or measurements after the pilot trial commenced, with reasons	N/A
6c		If applicable, prespecified criteria used to judge whether, or how, to proceed with future definitive trial	N/A

Sample size:			
7a	How sample size was determined	Rationale for numbers in the pilot trial	10
7b	When applicable, explanation of any interim analyses and stopping guidelines		
Randomisation:			
Sequence generation:			
8a	Method used to generate the random allocation sequence		
8b	Type of randomisation; details of any restriction (such as blocking and block size)	Type of randomisation(s); details of any restriction (such as blocking and block size)	7
Allocation concealment mechanism:			
9	Mechanism used to implement the random allocation sequence (such as sequentially numbered containers), describing any steps taken to conceal the sequence until interventions were assigned		
Implementation:			
10	Who generated the random allocation sequence, enrolled participants, and assigned participants to interventions		
Blinding:			
11a	If done, who was blinded after assignment to interventions (eg, participants, care providers, those assessing outcomes) and how		
11b	If relevant, description of the similarity of interventions		
Analytical methods:			
12a	Statistical methods used to compare groups for primary and secondary outcomes	Methods used to address each pilot trial objective whether qualitative or quantitative	10, 11
12b	Methods for additional analyses, such as subgroup analyses and adjusted analyses	Not applicable	
**Results**			
Participant flow (a diagram is strongly recommended):			
13a	For each group, the numbers of participants who were randomly assigned, received intended treatment, and were analysed for the primary outcome	For each group, the numbers of participants who were approached and/or assessed for eligibility, randomly assigned, received intended treatment, and were assessed for each objective	Fig 1, p11
13b	For each group, losses and exclusions after randomisation, together with reasons		
Recruitment:			

14a	Dates defining the periods of recruitment and follow-up		
14b	Why the trial ended or was stopped	Why the pilot trial ended or was stopped	10
Baseline data:			
15	A table showing baseline demographic and clinical characteristics for each group		
Numbers analysed:			
16	For each group, number of participants (denominator) included in each analysis and whether the analysis was by original assigned groups	For each objective, number of participants (denominator) included in each analysis. If relevant, these numbers should be by randomised group	Fig 1, 17
Outcomes and estimation:			
17a	For each primary and secondary outcome, results for each group, and the estimated effect size and its precision (such as 95% confidence interval)	For each objective, results including expressions of uncertainty (such as 95% confidence interval) for any estimates. If relevant, these results should be by randomised group	Table 3, 4
17b	For binary outcomes, presentation of both absolute and relative effect sizes is recommended	Not applicable	
Ancillary analyses:			
18	Results of any other analyses performed, including subgroup analyses and adjusted analyses, distinguishing prespecified from exploratory	Results of any other analyses performed that could be used to inform the future definitive trial	17
Harms:			
19	All important harms or unintended effects in each group (for specific guidance see CONSORT for harms)		
19a		If relevant, other important unintended consequences	13
**Discussion**			
Limitations:			
20	Trial limitations, addressing sources of potential bias, imprecision, and, if relevant, multiplicity of analyses	Pilot trial limitations, addressing sources of potential bias and remaining uncertainty about feasibility	19-22
Generalisability:			
21	Generalisability (external validity, applicability) of the trial findings	Generalisability (applicability) of pilot trial methods and findings to future definitive trial and other studies	19-22
Interpretation:			
22	Interpretation consistent with results, balancing benefits and harms, and considering other relevant evidence	Interpretation consistent with pilot trial objectives and findings, balancing potential benefits and harms, and considering other relevant evidence	19-22
22a		Implications for progression from pilot to future definitive trial, including any proposed amendments	
**Other information**			

Registration:			
23	Registration number and name of trial registry	Registration number for pilot trial and name of trial registry	4, 23
Protocol:			
24	Where the full trial protocol can be accessed, if available	Where the pilot trial protocol can be accessed, if available	Ref 17
Funding:			
25	Sources of funding and other support (such as supply of drugs), role of funders		
26		Ethical approval or approval by research review committee, confirmed with reference number	23

*Here a pilot trial means any randomised study conducted in preparation for a future definitive RCT, where the main objective of the pilot trial is to assess feasibility.

BMJ Open

A randomised controlled pilot trial of an exercise plus behaviour change intervention in people with multiple sclerosis: the Step it Up study

Journal:	*BMJ Open*
Manuscript ID	bmjopen-2017-016336.R2
Article Type:	Research
Date Submitted by the Author:	16-Aug-2017
Complete List of Authors:	Hayes, Sara; University of Limerick Faculty of Education and Health Sciences<br>Uszynski, Marcin; University of Limerick, Clinical Therapies<br>Motl, Robert; University of Alabama at Birmingham<br>Gallagher, Stephen; University of Limerick, Department of Psychology<br>Larkin, Aidan; Multiple Sclerosis Society of Ireland<br>Newell, John; NUI Galway<br>Scarrott, Carl; University of Canterbury, Mathematics and Statistics<br>Coote, Susan ; University of Limerick, Clinical Therapies; University of Limerick
**Primary Subject Heading**:	Neurology
Secondary Subject Heading:	Rehabilitation medicine
Keywords:	exercise, walking mobility, behaviour change, Multiple sclerosis < NEUROLOGY

SCHOLARONE™ Manuscripts

For peer review only

TITLE PAGE

A randomised controlled pilot trial of an exercise plus behaviour change intervention in people with multiple sclerosis: the Step it Up study

Sara Hayes[1]

Email: sara.hayes@ul.ie

Marcin Kacper Uszynski[1,3]

Email: marcin.uszynski@ul.ie

Robert W. Motl[5]

Email: robmotl@uab.edu

Stephen Gallagher[2]

Email: stephen.gallagher@ul.ie

Aidan Larkin[3]

Email: aidanL@MS-Society.ie

John Newell[4]

Email: john.newell@nuigalway.ie

Carl Scarrott[6]

Email: carl.scarrott@canterbury.ac.nz

*Susan Coote[1]

Email: susan.coote@ul.ie

[1] Department of Clinical Therapies, Faculty of Education and Health Sciences, Health Research Institute, University of Limerick, Limerick, Ireland

[2] Centre for Social Issues Research, Department of Psychology, Faculty of Education and Health Sciences, University of Limerick, Limerick, Ireland

[3] Multiple Sclerosis Society of Ireland, Dublin, Ireland

[4] HRB Clinical Research Facility and School of Mathematics, Statistics and Applied Mathematics, National University of Ireland, Galway, Ireland

For peer review only

For peer review only - http://bmjopen.bmj.com/site/about/guidelines.xhtml

[5] Department of Physical Therapy, School of Health Professions, University of Alabama at Birmingham

[6] HRB Clinical Research Facility, National University of Ireland, Galway, Ireland and School of Mathematics and Statistics, University of Canterbury, New Zealand

*Author for correspondence

For peer review only

ABSTRACT

Objective: to investigate feasibility of multiple sclerosis (MS) exercise guidelines for inactive people with MS (PwMS) and to examine preliminary efficacy for walking. To investigate effect of augmenting that intervention with education based on Social Cognitive Theory (SCT)

Design: pilot multicentre, double blind, randomised, parallel, controlled trial

Setting: community-delivered programme

Participants: Sixty-five physically inactive PwMS walked independently, scored 0–3 on the Patient Determined Disease Steps Scale, had no MS relapse or change in MS medication in 12 weeks

Interventions: 10-week exercise plus SCT education (SCT) compared with exercise plus attention control education (CON)

Outcome measures: Six Minute Walk Test (6MWT), Timed Up and Go (TUG) test and Multiple Sclerosis Walking Scale-12 (MSWS-12).

Results: 174 expressed interest, 92 were eligible and 65 enrolled (SCT,n=32, CON,n=33). The intervention was feasible and delivered as intended. 68% of SCT group and 50% of control group met the exercise guidelines after intervention.

Using linear mixed effects models, intention to treat basis, there was insufficient evidence for difference between the groups over the trial (6MWT p=0.30, TUG p=0.4, MSWS-12 p=0.8). Using secondary analysis of a cohort with data for ≥3 assessments (SCT n=21, CON n=20), there was significant treatment effect favouring the intervention group (p=0.04) with mean effect for 6MWT 39.0m(95%CI 2.26, 75.73) at 12 weeks and 40.0m(95%CI 2.3, 77.8) at 36 weeks. Both groups improved significantly in 6MWT following 10-week intervention (SCT mean Δ=83.02, sd=60.1, p=<0.01, CON mean Δ=56.92, sd=73.5 p=<0.01), TUG (SCT Δ=-0.70, sd=1.25, p=<0.01, CON Δ=-0.54, sd=0.95, p=<0.01), and MSWS-12 (SCT Δ=-8.03, sd=16.18, p=0.02, CON Δ=-0.86, sd=18.74, p=0.81).

Conclusions: A 10-week exercise programme based on the MS exercise guidelines for improving walking in previously inactive people with MS was feasible. There is marginal evidence of a treatment effect in favour of the exercise plus SCT intervention at 12 and 36 weeks.

For peer review only - http://bmjopen.bmj.com/site/about/guidelines.xhtml

Trial registration NCT02301442.

Funding: Health Research Board, Ireland

Keywords Exercise, walking mobility, Social Cognitive Theory, behaviour change, multiple sclerosis

STRENGTHS AND LIMITATIONS

- New evidence demonstrating the feasibility and preliminary efficacy of delivering a pragmatic, combined, community-based exercise and Social Cognitive Theory education intervention for physically inactive people with MS based on the MS Exercise Guideline
- The use of measures of fidelity, assessments of the target variables of the intervention (strength, fitness and physical activity) and both self-report and objective measures of walking mobility
- Treatment fidelity was considered and evaluated, yet a limitation relates to the use of a 1-day training course for physiotherapists, in particular relating to the novel use of education techniques throughout the exercise programme.
- Attrition of participants between determining eligibility and starting the intervention; the long wait times meant that 29% of eligible participants were lost at this phase.

For peer review only - http://bmjopen.bmj.com/site/about/guidelines.xhtml

INTRODUCTION

Walking limitations are the hallmark of multiple sclerosis (MS)[1] and people with MS report that walking limitations are a significant concern[2]. Indeed, walking limitations have been associated with change in occupation due to MS and occupational disability [3] and influence a range of other outcomes such as cognition and depression[4]. Exercise training remains the cornerstone therapeutic intervention for the management of walking limitations in MS. Many studies report positive effects from a range of exercise interventions as summarised in recent reviews [5] and meta-analyses[6 7] that confirm combined aerobic and resistance exercises can improve both walking speed and walking endurance.

The recent exercise guidelines recommend aerobic exercise twice a week and resistance exercise twice a week as the minimum target for improving walking outcomes among people with mild-to-moderate MS [8]. To that end, we demonstrated using a pragmatic, randomised controlled trial design, that 10 weeks of combined aerobic and resistance training delivered in groups in the community yielded positive improvements in 6MWT [9]. Of concern, however, was that these improvements were not maintained at three-month follow-up[10].

The maintenance of long-term exercise behaviour change is not a problem that is unique to MS, and researchers have highlighted the need for inclusion of behavioural approaches based on theory for long-term behaviour change [11]. Social cognitive theory (SCT) has been most commonly investigated in MS and its domains of exercise self-efficacy and goal setting are consistently associated with physical activity (PA) behaviour [12]. We have reported improvements in PA, and secondary outcomes including walking, from an SCT-based online intervention in MS [13], and one study demonstrated that physical activity behaviour change was maintained three months after cessation of the program[14]. This education program was originally designed based on a RCT of a SCT-based exercise intervention delivered in older adults [15] and later modified and tested for MS [16].

We designed a randomised controlled pilot trial called 'Step it Up' [17] that combined a group exercise programme with a theory-based education component for augmenting the effect of exercise on

For peer review only - http://bmjopen.bmj.com/site/about/guidelines.xhtml

walking outcomes and sustaining these changes over time. The aim of this study was to investigate the feasibility of delivering the combined interventions by physiotherapists and to establish preliminary clinical efficacy for improving walking outcomes; secondary outcomes will be provided in a parallel publication. We delivered the same exercise programme to both groups and controlled for contact by comparing a structured SCT education programme with an attention control education programme, and investigated whether adding the SCT education component would yield greater improvements in walking mobility and whether the improvements were maintained at follow-up. It was hypothesized that that the participants in the exercise and SCT-based intervention would achieve significantly more improvement in walking outcomes than the control group post-intervention and that this improvement would be maintained at follow up. The results of this trial will inform the design, particularly power analysis, of a definitive trial that provides Class 1 evidence (AAN).

METHODS

Study design

This was a multicentre, two-arm, parallel (1:1), double blind, randomised controlled trial.

Setting and participants

The participants were recruited through the MS Society of Ireland, and via neurology clinics in three urban locations in the Republic of Ireland. Details of the recruitment process are further detailed in the protocol paper[17]. Inclusion criteria were: (1) physician-confirmed formal diagnosis of MS, (2) aged 18 years or more, (3) Patient Determined Disease Steps (PDDS) scale score of 0–3, (4) a sedentary lifestyle (<30 minutes of moderate to strenuous exercise one day or more per week over the last six months) and (5) willing to give written informed consent. Exclusion criteria included: (1) pregnancy, (2) MS relapse in the previous 12 weeks and (3) changes to MS medication or steroid

For peer review only - http://bmjopen.bmj.com/site/about/guidelines.xhtml

treatment in the previous 12 weeks. Participants were sent the consent form in advance of the baseline assessment, and written consent was obtained in person.

Randomisation and blinding

Participants were randomly allocated into the exercise plus SCT-based intervention or the exercise plus contact control education intervention. Random allocation procedures have been previously outlined[17] and were adhered to. JN generated the random allocation sequence, the SH enrolled participants, and SC assigned participants to interventions. The outcome assessor (SH) was blind to allocation throughout the study as was the statistician CS during the analysis. All participants were informed that the study aimed to examine the effect of combining exercise and education, and therefore were blinded regarding group allocation.

Screening questionnaire

Potential participants were screened for eligibility for this study using a questionnaire that included the Patient Determined Disease Steps (PDDS) scale[18], confirmation of formal MS diagnosis and questions regarding PA levels. The PDDS scale contains a single item for measuring self-reported neurological impairment on an ordinal level from zero (Normal) to eight (Bedridden). Scores from the PDDS are linearly and strongly related with physician-administered Expanded Disability Status Scale (EDSS) scores[18] .

Outcome measures

Outcome measures were conducted pre-intervention (week 1), post-intervention (week 12), and at 24- and 36-week follow-up.

For peer review only - http://bmjopen.bmj.com/site/about/guidelines.xhtml

Demographic and clinical information

Participants provided details regarding age, gender, level of formal education, time since diagnosis of MS, duration of symptoms of MS, falls history, exercise history, marital status and employment status. Additionally, a researcher formally trained in the use of the Expanded Disability Status Scale (EDSS) (SH) administered the EDSS to all participants at baseline. The EDSS quantifies MS disease progression and is commonly the standard that other outcome measures are compared against [19]. It consists of functional systems subscales and a total score which is an ordinal rating ranging from 0 (normal neurological status) to 10 (death due to MS). MS diagnosis according to the McDonald or Poser criteria was confirmed from the participant's consultant neurologist.

Primary outcomes

The primary outcome was walking mobility at week 36. This was measured using the Six Minute Walk Test (6MWT) as the primary endpoint. The participants were instructed to walk as quickly and as safely as possible for six minutes on a ten meter track. The 6MWT has demonstrated excellent test-retest reliability and concurrent validity among people with mild to moderate MS[20].

We further used the Timed Up and Go test (TUG) and the Multiple Sclerosis Walking Scale-12 (MSWS-12). The TUG has demonstrated excellent test-retest reliability for people with mild MS [21] and the MSWS-12 has demonstrated excellent internal consistency[22 23], test-retest reliability[24] and concurrent validity in people with MS[25].

Adherence

Adherence to the intervention was documented throughout the 10-week intervention via exercise logs. The exercise logs captured attendance at the exercise classes and home exercise sessions. Over the 10-week intervention, 44 total sessions were made available to the participants. This included six exercise classes with strengthening and coaching/education components, four coaching phone calls, 14 prescribed home strengthening sessions, and 20 prescribed home walking sessions.

For peer review only - http://bmjopen.bmj.com/site/about/guidelines.xhtml

We further evaluated adherence to the exercise component by evaluating the effect on strength, fitness and physical activity. The 5 times sit to stand test (5xSTS)[26] (time to complete 5 sit to stand repetitions in seconds) measured lower extremity muscle strength. The Modified Canadian Aerobic Fitness Test (mCAFT)[27] measured fitness and was calculated using following equation; 10 x [17.2 + (1.29 x O2 cost of last stage) – (0.09 x body mass in kg) – (0.18 x Age)]. The Health Index of the Godin Leisure-Time Exercise Questionnaire (GLTEQ)[28] measured PA behaviour. These measures and associated psychometric properties have been described in the trial protocol[17].

Interventions

The content of the interventions delivered in both arms of this RCT has been outlined in detail in the protocol paper[17]. The exercise intervention was common to both groups and was delivered by physiotherapists. The aim of the exercise component was to progressively increase the intensity of both aerobic and strengthening activities to enable the participants to reach the published exercise guidelines for people with mild-to-moderate MS[8], and has been previously described[17]. Over the 10-week programme participants attended the group exercise class at community venues on six occasions, supplemented with a telephone coaching call in the weeks without classes (intervention weeks 4, 6, 7 and 9). After each of the group exercise classes the control group received an education session about topics unrelated to PA behaviour, e.g. diet, vitamin D, sleep, temperature and hydration, and immunisations and vaccinations. The exercise plus SCT-based intervention group received the same exercise intervention as the control group (as described in the previous section). This group also received a similar duration of education based on the principles of SCT for health behaviour change, namely: self-efficacy, outcome expectations, goal-setting, barriers and benefits and has been previously described[17]. The SCT intervention was designed to enable continued exercise behaviour and after the 10-week intervention the participants in both groups received structured phone calls from the intervention physiotherapists at weeks 16, 20 and 36. These telephone calls consisted of direct questions about the frequency, intensity, type and duration of

For peer review only - http://bmjopen.bmj.com/site/about/guidelines.xhtml

exercise participants had completed and whether they had experienced any adverse events or relapses. Additionally the SCT group were coached using the principles of that educational component.

Treatment fidelity

All of the physiotherapists who delivered the intervention or control group sessions were provided with a one-day training course on the delivery of the intervention for their group, directly related to the manual of operating procedures[17]. The intervention was delivered at three sites over the course of the study by 8 physiotherapists broadly representative of those working in primary care. Continued support from the research centre was available if additional training was needed. The fidelity of the physiotherapists' sessions, including both exercise and SCT components, was monitored by randomly allocated video and audio recording of at least one of the intervention sessions. An independent assessor compared the content of the intervention manuals with the video or audio recordings.

Statistical analysis

Sample size

Consistent with data from a large international study[29], it was hypothesised that the effect of the intervention would yield an average improvement in 6MWT distance of 36m with an estimated standard deviation of 48.2m. In order to have 80% power (at the 5% significance level) to detect such a difference in mean improvement in 6MWT over the study period between groups, a sample of size 62 randomised equally to two arms (i.e. 31 per arm) was utilized to inform the target sample size for this pilot study. The intention was to recruit 72 participants to account for drop out and to run the group interventions once sufficient people in that region were eligible. Recruitment in regions was not uniform and participants became ineligible while waiting for others to be recruited.

For peer review only - http://bmjopen.bmj.com/site/about/guidelines.xhtml

For peer review only

Recruitment was better than intended and continued to 92 eligible participants resulting in 65 participants starting the intervention.

Suitable numerical statistics and graphical summaries were used to describe characteristics of the sample at baseline and to assess the validity of any distributional assumptions needed for the formal analysis. All tests of significance were two-sided and conducted at an alpha = 0.05 level of statistical significance. An exploratory paired t-test between baseline and each of the week 12, 24 and 36 follow-ups are conducted, provides a summary of the effects of the estimated treatment and control from the raw data. These “unadjusted” results do not account for the patient covariates and repeated measurements. We also quantified and compared the magnitude of change in walking measures using Hedges’ g effect sizes and associated 95% confidence intervals (95%CI) using Cohen’s conventions for effect sizes (0.2 small, 0.5 moderate, 0.8 large). For each outcome measure, the mean baseline to post intervention and 3 and 6 month change for the control condition was subtracted from the mean baseline to post intervention and 3 and 6 month change for the intervention condition and divided by the pooled baseline standard deviation[30]. Effect sizes were calculated such that greater improvements in outcomes in the intervention group compared to the control group resulted in positive effect sizes.

The statistical modelling compared differences in the response variables (6MWT, TUG and MSWS scores) between the two intervention arms at each of the three post-intervention follow-ups while correcting for the baseline measurements for each participant. A linear mixed model for a continuous response over time due to the two interventions, whilst adjusting for participant-specific covariates and factors; namely 6MWT at baseline, age, gender, time since diagnosis and MS type (i.e. benign, primary progressive and relapsing-remitting). Treatment and time (and their interaction) were specified as fixed effects, centre (three levels) and subject (nested in centre) as random effects

For peer review only - http://bmjopen.bmj.com/site/about/guidelines.xhtml

in order to account for homogeneity within centre and within subject correlation over time. Initially a model containing the main effects of the treatment, time and a treatment-by-time interaction was specified in order to test whether there is evidence that the treatment effects varies over time. If the interaction was deemed unnecessary (using a likelihood ratio test) the model was refitted excluding the interaction term, so the treatment effect was then constant over time. Two separate analyses were carried out. Firstly, following an intention-to-treat principle in which all 65 patients who remained eligible to participate were considered. In the secondary analysis, a smaller cohort of 52 patients are analysed, who were identified to have closely adhered to the program by having attended at least two of the three follow-ups. All models were fitted in R 3.2.0 using the lme4 and lmerTest packages. Model diagnostics involved suitable plots of the residuals.

RESULTS

Participant sample

One hundred and seventy-four people with MS contacted the trial centre and were screened for inclusion over the phone between September 2013 and May 2014. Eighty-two people were excluded as per the selection criteria (Figure 1) and recruitment ceased when 92 people were randomised to either of the trial arms. Between time of randomisation and initiation of the intervention, 27 eligible participants either became in-eligible or were unable to participate. One participant was not treated as randomised (two acquaintances had been randomised to the other group and they wanted to exercise with them). Sixty-five participants commenced the intervention (SCT group n=33, CON group n=32). In the SCT group, four participants discontinued the intervention and 12 were lost to follow-up at 36-weeks. In the CON group, three participants discontinued the intervention and 10 were lost-to-follow up at 36 weeks. Following the 10-week intervention overall attrition was 17% and at the 36-week follow-up assessment attrition was 34%. Reasons for discontinuing the intervention and loss to follow-up are outlined in Figure 1. Baseline characteristics for both groups are shown in Table 1.

For peer review only - http://bmjopen.bmj.com/site/about/guidelines.xhtml

Table 1 Clinical baseline characteristics in exercise plus SCT group (SCT) and exercise plus education control group (CON)

	SCT (n=33)	CON (n=32)
**MS type**		
Benign	3	1
Primary progressive	1	0
Relapsing-remitting	27	27
Secondary progressive	0	1
Unknown	2	2
**EDSS (median, IQR)**	3.3 (0.7)	3.3 (0.7)
**Years since diagnosis**	6.7 (5.7)	7.0 (6.1)
**Centre (n)**		
Cork	10	9
Galway	8	10
Limerick	15	13
**Age**	43.3 (9.9)	41.9 (9.3)
**Gender (n)**		
Male	4	6
Female	29	26

EDDS: Expanded Disability Disease Scale; IQR: interquartile range; Data given as mean (SD) unless otherwise indicated

Treatment fidelity

An independent person to the intervention (PO'S) used the manual of operating procedures to check if the required content of the programme (both exercise and SCT/attention control education components) was delivered as intended. In both trial arms, 100% of the content of the supervised sessions were implemented as described in the intervention manual.

Feasibility - Exercise Logs

The development of hip pain by one participant in the CON group was the only related adverse event reported by participants in both trial arms during the completion of the 10-week intervention. The SCT and the CON group groups completed an average of 33.2 of 44 available sessions (75.5%) and 32.0 sessions (72.6%), respectively. The proportion of sessions completed is presented in Figure 2, wherein the lowest number of sessions was in week 7 when participants were exercising independently without a class for a second consecutive week. Among the 53 participants who

For peer review only - http://bmjopen.bmj.com/site/about/guidelines.xhtml

provided detailed exercise logs, 17 (68%) of the SCT group and 14 (50%) of the CON group were exercising at the minimum recommended by the exercise guidelines by the end of the 10-week intervention. The reasons for not meeting the guideline included: walking less than 30 minutes twice per week (SCT n=3, CON n=1), walking only once per week (SCT n=2 CON n=5) and doing only one set of each resistance exercise per week (SCT n=2, CON n=4).

In order to further evaluate the adherence to the intervention we investigated the change in strength, fitness and physical activity in order to evaluate whether the intervention changed these intended parameters. Table 2 presents the raw data and unadjusted comparisons. For both groups there were significant improvements in PA and strength from weeks 1 to 12. There was a tendency for aerobic fitness scores to increase, but this change was not statistically significant.

Table 2 Raw data and unadjusted comparisons of change in secondary outcomes from week 1 to week 12 in exercise plus SCT group (SCT) and exercise plus education control group (CON)

		**Week 1 Mean (SD)**	**Week 12 Mean (SD)**	**Mean change from week 1 to week 12 (95% CI) *p*-value**
**Godin Health Index**	SCT	3.03 (6.19)	12.48 (11.15)	9.85 (5.46, 14.23) $p<0.01$
	CON	1.88 (4.88)	16.07 (21.12)	12.92 (4.96, 20.89) $p<0.01$
**Five Times Sit to Stand**	SCT	11.48 (2.7)	9.78 (2.18)	-1.51 (-2.42, -0.60) $p<0.01$
	CON	10.8 (2.6)	9.43 (1.93)	-1.55 (-2.30, -0.79) $p<0.01$
**Aerobic Fitness Score**	SCT	295.72 (54.61)	309.12 (53.78)	8.58 (-6.86, 23.98) $p=0.26$
	CON	313.56 (59.02)	331.29 (51.57)	10.54 (-6.29, 27.37) $p=0.21$

CI: confidence interval

For peer review only - http://bmjopen.bmj.com/site/about/guidelines.xhtml

Walking mobility

The mean (SD) scores for the 6MWT, TUG and MSWS-12 at weeks 1, 12, 24 and 36 for participants in the exercise plus SCT and exercise plus education control groups are presented in Table 3. Figure 3 shows the results of the estimated treatment effects on 6MWT, TUG and MSWS-12, as per intention-to-treat and secondary analyses, respectively. The unadjusted, unstandardized mean changes from baseline, and 95% confidence intervals and paired t-test results for both groups are presented in Table 4 along with Hedges G effect sizes. Both groups demonstrated an improvement in the primary outcome, 6MWT and secondary outcome MSWS from weeks one to 12 and at 24 and 36 week follow up. For TUG the result are a little more mixed, with evidence of an improvement in both groups from weeks one to 12 which diminishes in the control group by week 36 but a persistent significant difference is observed in the education with SCT group from baseline to weeks 24 and 36.

For peer review only

For peer review only - http://bmjopen.bmj.com/site/about/guidelines.xhtml

Table 3 Mean (SD) walking mobility outcomes at weeks 1, 12, 24 and 36 in exercise plus SCT group (SCT) and exercise plus education control group (CON)

	Week 1		Week 12		Week 24		Week 36	
	Intention to Treat Analysis							
Outcome variable	SCT	CON	SCT	CON	SCT	CON	SCT	CON
6MWT	445.2 (68.8)	482.0 (72.0)	527.4 (91.1)	547.1 (96.0)	492.8 (73.5)	504.9 (76.9)	515.8 (91.0)	528.0 (93.2)
TUG	7.06 (1.61)	6.51 (1.36)	6.27 (1.45)	5.81 (1.08)	6.23 (1.26)	6.00 (0.98)	5.93 (1.33)	5.96 (1.20)
MSWS-12	38.0 (28.0)	33.3 (24.8)	29.6 (22.2)	30.8 (21.3)	31.9 (22.1)	26.3 (21.5)	32.6 (23.4)	27.9 (21.9)
	Secondary Analysis							
	SCT	CON	SCT	CON	SCT	CON	SCT	CON
6MWT	434.6 (65.2)	474.4 (69.6)	524.2 (96.7)	535.2 (88.0)	496.2 (73.7)	504.9 (76.9)	515.8 (91.0)	528.0 (93.2)
TUG	7.08 (1.73)	6.65 (1.36)	6.43 (1.46)	5.87 (1.13)	6.30 (1.25)	6.00 (0.98)	5.93 (1.33)	5.96 (1.20)
MSWS-12	38.2 (26.7)	31.9 (22.6)	29.7 (22.6)	32.6 (21.0)	31.9 (22.1)	26.3 (21.5)	32.6 (23.4)	27.0 (21.8)

6MWT: Six Minute Walk Test; TUG: Timed Up and Go; MSWS-12: Multiple Sclerosis Walking Scale-12

For peer review only

Table 4 Unadjusted comparisons of change in walking measures from week 1 to weeks 12, 24 and 36 in exercise plus SCT group (SCT) and exercise plus education control group (CON)

	Mean change week 1 to week 12 (95% CI) *p*-value			Mean change week 1 to week 24 (95% CI) *p*-value			Mean change week 1 to week 36 (95% CI) *p*-value		
	**SCT**	**CON**	***Hedges G (95%CI)***	**SCT**	**CON**	***Hedges G (95%CI)***	**SCT**	**CON**	***Hedges G (95%CI)***
**6MWT**	83.02 (58.74, 107.29) *p*<0.01	56.92 (28.43, 85.41) *p*<0.01	0.37 (-0.12, 0.86)	55.97 (32.12, 79.84) *p*<0.01	34.2 (13.43, 54.97) *p*<0.01	0.31 (-0.18, 0.80)	82.18 (50.90, 113.45) *p*<0.01	46.87 (18.57, 75.17) *p*<0.01	**0.50** (0.01, 0.96)
**TUG**	-0.70 (-1.20, -0.19) *p*<0.01	-0.54 (-0.91, -0.17) *p*<0.01	0.11 (-0.59, 0.38)	-0.79 (-1.19, -0.38) *p*<0.01	-0.74 (-1.13, -0.35) *p*<0.01	0.03 (-0.52, 0.45)	-1.23 (-1.68, -0.78) *p*<0.01	-0.57 (-0.98, -0.16) *p*<0.01	0.44 (-0.05, 0.93)
**MSWS-12**	-8.03 (-14.43, -1.63) *p*=0.02	-0.86 (-7.99, 6.27) *p*=0.81	0.27 (-0.22, 0.76)	-6.43 (-12.10, -0.77) *p*=0.03	-2.88 (-11.41, 5.64) *p*=0.49	0.13 (-0.35, 0.62)	-8.62 (-15.90, -1.34) *p*=0.02	-5.60 (-13.84, 2.64) *p*=0.17	0.11 (-0.37, 0.60)

6MWT: Six Minute Walk Test; TUG: Timed Up and Go; MSWS-12: Multiple Sclerosis Walking Scale-12. Bold text indicates moderate effect size.

The linear mixed models results in Table 5 shows that using an intention-to-treat analysis there was no evidence of a significant treatment effect in favour of the exercise plus SCT compared to the exercise only group for regarding 6MWT, TUG or MSWS scores. Figure 3 confirms the obvious significant effects of the exercise programme found above in the unadjusted paired t-test results, which is shown by the blue and red lines being well above the black “no effect” line when the sample uncertainty conveyed by the corresponding confidence intervals are taken into account. But Figure 3 also confirms lack of evidence for an additional effect of the SCT over the usual exercise programme, which is shown by the widely overlapping confidence intervals between the treatment and control groups.

For peer review only - http://bmjopen.bmj.com/site/about/guidelines.xhtml

Table 5 Estimated treatment effects at weeks 12, 24 and 36 in primary outcome

	Estimate of difference between SCT and Control	Standard error	95% CI	*p*-value
Intention-to-treat analysis				
**6MWT**				
Week 12	22.70	19.00	(-15.14, 60.50)	*0.23*
Week 24	11.80	20.40	(-28.77, 52.36)	*0.56*
Week 36	27.42	20.35	(-13.06, 67.90)	*0.18*
**TUG**				
Week 12	0.069	0.236	(-0.402, 0.541)	*0.77*
Week 24	-0.132	0.250	(-0.630, 0.365)	*0.60*
Week 36	-0.457	0.252	(-0.960, 0.045)	*0.08*
**MSWS-12**				
Week 12	-4.91	4.47	(-13.82, 4.00)	*0.28*
Week 24	-0.59	4.69	(-9.91, 8.73)	*0.90*
Week 36	0.38	4.57	(-8.71, 9.47)	*0.93*
Secondary analysis				
**6MWT**				
Week 12	39.00	18.44	(2.26, 75.73)	*0.04*
Week 24	27.44	19.23	(-10.82, 65.70)	*0.16*
Week 36	40.03	18.97	(2.27, 77.79)	*0.04*
**TUG**				
Week 12	0.204	0.255	(-0.306, 0.713)	*0.43*
Week 24	-0.020	0.261	(-0.542, 0.502)	*0.94*
Week 36	-0.367	0.262	(-0.890, 0.156)	*0.17*
**MSWS-12**				
Week 12	-7.63	4.65	(-16.89, 1.63)	*0.11*
Week 24	-2.50	4.78	(-12.01, 7.02)	*0.60*
Week 36	-1.57	4.69	(-10.93, 7.78)	*0.74*

For peer review only

6MWT: Six Minute Walk Test; TUG: Timed Up and Go; MSWS-12: Multiple Sclerosis Walking Scale-12

A secondary analysis was completed with participants who attended at least two of the three follow-up assessments (SCT n=25, CON n=27). Table 3 presents the mean (SD) scores for the 6MWT, TUG and MSWS-12 at weeks 1, 12, 24 and 36 for participants in the SCT and control groups using secondary analysis. For 6MWT, the SCT group had a marginally more positive outcome, with statistically significant treatment effects evident at weeks 12 and 36 (Table 5). Using this secondary analysis there was no evidence of a treatment effect in favour of the SCT group as compared to the CON group regarding the TUG or MSWS-12 scores.

DISCUSSION

This pilot RCT investigated the feasibility and preliminary efficacy of the Step it Up programme, a 10-week aerobic and strengthening programme that aimed to enable physically inactive people with MS to exercise according to the recent MS exercise guidelines [8]. We investigated whether embedding an evidence-based exercise programme within a structured SCT-based education programme resulted in improved and more sustained walking outcomes compared to an exercise plus attention control education intervention. To our knowledge, this is the first study to examine the effect of enabling inactive people to meet the minimum recommended dose of the MS exercise guidelines and examine the effects on walking mobility as a primary end-point.

The intervention protocol was feasible and results demonstrated significant improvements in walking mobility following the intervention in both groups. There was a moderate effect (Hedges G 0.50) at 36 week follow up in favour of the SCT group for 6MWT. The effect for the SCT group was also greater at 12- and 36-week follow-up for the primary outcome, 6MWT, using the secondary analysis which included only patients who adhered to the program (as defined by having attended at least two of the three follow-ups). Recruitment was successful and over nine months at three centres we recruited more than our target of 62 participants (92 eligible participants). The largest

For peer review only - http://bmjopen.bmj.com/site/about/guidelines.xhtml

point of attrition was while participants waited for enough people to run the group in that region. In the future, recruiting from the largest city in Ireland for a definitive RCT will enable greater numbers to be recruited more quickly and should minimise this attrition at this point in the trial. Retention across the intervention period was good and the attrition rate (17%) was similar to other exercise interventions in people with depression[31] and slightly higher than the average of 15% in a review of exercise trials in MS[32]. While the level of participant attrition in the current programme is greatly improved from our previous community based exercise RCT[9 10], measures such as recruiting a dedicated study coordinator to provide more frequent interactions with participants in the definitive trial will be explored to further enhance retention at follow-up. The addition of booster intervention sessions after the completion of the 10-week intervention will also be explored in the future definitive trial.

The intervention was delivered by physiotherapists who attended a 1-day training session and treatment fidelity findings suggest that this approach was successful as the interventions were delivered as intended; further training and support may increase the success of the intervention in future. Participants completed on average 73 to 75% of possible sessions suggesting that the protocol is feasible for participants with minimal impairment due to MS. We collected data from exercise logs for demonstrating adherence with the exercise programs. The exercise logs were returned by 82% of participants and used to ascertain whether participants were meeting the MS exercise guideline at the end of the intervention period. It is interesting to note that a greater proportion of participants in the SCT group (68% versus 50%) progressed to meeting the guidelines. Measures to further enhance completion and return of logs (such as offering them in alternative electronic formats) in the future definitive trial are needed.

We further confirmed adherence to this aerobic and strengthening intervention by investigating its effects on strength, fitness and PA. Both 5xSTS and Godin Health index increased significantly and the AFS showed a tendency to improve providing evidence that the exercise intervention met its intended outcomes. Collectively, we believe that the exercise log data combined with fitness and PA

For peer review only - http://bmjopen.bmj.com/site/about/guidelines.xhtml

outcomes support the successful manipulation of exercise behaviour with in both trial arms. Based on the data on recruitment, retention, feasibility and preliminary efficacy of this group exercise and SCT education intervention we propose to progress to a definitive intervention. To do this, a sample of 49 (for a difference between groups of 39m, assuming a standard deviation of the change score at 36 weeks of 67.85, 80% power, 0.05 significance level) in each group would be needed and we therefore plan to recruit across these three centres again and to add a 4th centre in the largest city in Ireland to minimise attrition.

Importantly, both groups improved significantly in the primary outcome, 6MWT, following the intervention. This improvement in 6MWT is consistent with a recent systematic review of exercise studies that found a significant improvement in walking endurance[7]. We note that the mean improvement in the SCT group of 80m and of 60m in the control group far exceeded the value for the clinically important change of 26.1m proposed by Baert et al[29]. Both groups improved more than that reported by Carter et al (2012)[33] in their exercise plus education group, and the magnitude of improvement is more consistent with the improvements noted in a recent community-based intervention among people with moderate-severity MS[34]. We further note that the current physically inactive sample of people with MS with an average age of 42 had 6MWT of 445m at baseline that was less than that of a reference sample aged 70-80 years who walked an average of 514m[35]. This confirms the significant walking impairments for inactive people with mild disability with MS and importantly demonstrates positive improvements due to the Step it Up exercise intervention. Interestingly the SCT group but not the CON group improved in their self-reported walking impairment (MSWS-12) and the magnitude of the change in 6MWT distance may have influenced that finding. Both groups however improved in walking speed and maintained that improvement at 36 week follow-up with a small-moderate effect size in favour of the SCT group demonstrated for TUG.

For peer review only - http://bmjopen.bmj.com/site/about/guidelines.xhtml

Of note, through the secondary analysis including participants who participated in at least two follow-up assessments, we demonstrated that adding a structured SCT education programme enhanced the effect on 6MWT distance following the 10-week intervention. This is important as it provides information on the preliminary effectiveness of the intervention and confirms the need to augment the retention strategies in the definitive trial. We propose greater training for the interventionists, and greater use of telephone coaching in weeks without classes and between intervention and follow up sessions. Importantly, both the improvement from baseline and the difference in between-group effects were maintained at 36-week follow-up providing new information on the ability to sustain effects after the intervention ceased. Interestingly the effect was reduced at 24 weeks and participants reported that realising they had deteriorated at that assessment served as a prompt to resume their exercise after that assessment. The SCT education programme had six education sessions that targeted outcome expectancies, self-efficacy, goal setting, and perceived barriers and benefits of exercise. The components are further consistent with a recent systematic review and meta-analysis of modifiable psychosocial constructs associated with PA in MS that confirmed self-efficacy, goal setting and outcome expectancies as significantly correlated with PA in MS[36]. One novel feature of the current trial is that the SCT education modules were delivered by physiotherapists with minimal training in delivery of behavioural interventions. These findings also support that delivering this SCT education intervention by physiotherapists in a group setting is both feasible and preliminary findings suggest that it may have superior outcomes to an attention control education intervention.

Strengths and Limitations

One of the strengths of this pilot RCT relates to the production of new knowledge around the sustainability of exercise interventions for people with MS. Building on the existing evidence base, we designed and delivered a SCT-based pragmatic physiotherapist-led community exercise. Results demonstrated the feasibility of the protocol among physically-inactive people with mild MS and

For peer review only - http://bmjopen.bmj.com/site/about/guidelines.xhtml

trends towards clinical efficacy for walking outcomes. The model of care outlined in this pilot study presents as a highly-scalable intervention package for physiotherapists and other healthcare professionals working in primary care services or with 3rd sector organisations (charities). Further study in the form of a definitive trial, including cost and clinical outcomes has the potential to have real policy implications for the provision of rehabilitation to people with MS. Further strengths relate to the use of measures of treatment fidelity, target variables of the intervention (strength, fitness and PA) and both self-report (MSWS) and objective (TUG and 6MWT) measures of walking. Additionally, in the context of an evidence base wherein PA interventions are often not theoretically-based, a key strength of this RCT is the use of the SCT framework to design a behaviour-change intervention; building on the extensive work of the US partner in this trial.

One limitation is the attrition of participants between point of eligibility and allocation to the intervention. The large waiting times resulted in the loss of 29% of eligible participants at this point in the trial. Recruitment from larger urban areas with greater numbers of both MS clinics and people with MS is planned for the future definitive trial so that the numbers required to run group classes are met more quickly. A further positive is that we used pedometers and exercise logs to record the intensity and duration of the intervention; however another limitation is that detailed exercise diaries were not returned for all participants. However, a return rate of 82% is acceptable and measures to improve this in the definitive trial will be considered.

Conclusion

This pilot RCT aimed to investigate the feasibility and preliminary efficacy of enabling physically inactive people with MS to meet the MS exercise guidelines [8] through a group exercise and education, physiotherapist-led intervention. We further sought to investigate whether the theory-based SCT component was superior to an attention control education intervention. We found that recruitment was successful, though measures to improve retention in a future definitive trial are needed. Attrition over the intervention and follow-up periods were improved compared to our

For peer review only - http://bmjopen.bmj.com/site/about/guidelines.xhtml

previous exercise trial [9]. The programme resulted in significant improvements in walking endurance and speed for both groups. There was a moderate effect (Hedges G 0.50) for 6MWT at 36 weeks which is supported by a secondary analysis of those with data for three of four assessment points which demonstrated there was a significant effect in favour of the exercise plus SCT groups compared to the exercise plus control education group at weeks 12 and 36. This supports the preliminary sustained efficacy of the intervention and we propose progressing to a definitive intervention.

Contributors

SH was a post-doctoral researcher on the trial, contributed to the design of the study, collected data, drafted the paper and approved the final version. MU was a post-doctoral researcher on the trial, commented on drafts of the paper and approved the final version. RM co-initiated the project and contributed to the design of the trial, drafted the paper and approved the final version. SG contributed to the design, delivery and evaluation of the trial, commented on drafts of the paper and approved the final version. AL contributed to the recruitment strategy employed, commented on drafts of the paper and approved the final version. JN and CS were the statisticians on the trial, cleaned and analysed the data, commented on drafts of the paper and approved the final version. SC was the principal investigator for the study, co-initiated the project, contributed to the design of the trial, drafted the paper and approved the final version.

Funding This work is supported by the Irish Health Research Board Health Research Award, grant number: HRA_PHR/2013-264.

Acknowledgements The authors would like to thank MS Ireland for their assistance with recruitment and running this trial. We would also like to thank Paraic O'Suilleabhain who assisted with data for this paper.

Competing interests All authors have completed the ICMJE uniform disclosure form at www.icmje.org/coi_disclosure.pdf and declare: no support from any organisation for the submitted work; no financial relationships with any organisations that might have an interest in the

For peer review only - http://bmjopen.bmj.com/site/about/guidelines.xhtml

submitted work in the previous three years; no other relationships or activities that could appear to have influenced the submitted work.

Ethics approval was given by the Faculty of Education and Health Science Research Ethics Committee, University of Limerick (2014_02_20_EHS), in addition to the Research Ethics Committees at the University College Hospital Galway, University Hospital Limerick and Cork University Hospital.

Trial registration NCT02301442

Data sharing statement All data requests pertaining to the Step it Up trial should be made directly to susan.coote@ul.ie

Figure Legends

Figure 1 CONSORT Flow Diagram

Figure 2 Proportion of participants completing sessions (Exercise Diary data).

Figure 3 – Estimated effects using intention to treat and secondary analysis

Appendix 1 – TIDieR checklist

Appendix 2 – Exercise Logs

Appendix 3 - Exercise drawings

REFERENCES

1. Motl RW. Ambulation and Multiple Sclerosis. *Physical Medicine and Rehabilitation Clinics of North America* 2013;24(2):325-36. doi: http://dx.doi.org/10.1016/j.pmr.2012.11.004
2. Heesen C, Bohm J, Reich C, et al. Patient perception of bodily functions in multiple sclerosis: gait and visual function are the most valuable. *Mult Scler* 2008;14(7):988-91.
3. Goldman MD, Motl RW, Scagnelli J, et al. Clinically meaningful performance benchmarks in MS: timed 25-foot walk and the real world. *Neurology* 2013;81(21):1856-63.
4. Learmonth YC, Ensari I, Motl RW. Cognitive motor interference in multiple sclerosis: Insights from a systematic, quantitative review. *Arch Phys Med Rehabil* 2016 doi: 10.1016/j.apmr.2016.07.018 [published Online First: 2016/08/21]
5. Latimer-Cheung AE, Pilutti LA, Hicks AL, et al. Effects of exercise training on fitness, mobility, fatigue, and health-related quality of life among adults with multiple sclerosis: A systematic review to inform guideline development. *Archives Of Physical Medicine And Rehabilitation* 2013;94(9):1800-28. doi: 10.1016/j.apmr.2013.04.020
6. Learmonth YC, Ensari I, Motl RW. Physiotherapy and walking outcomes in adults with multiple sclerosis: systematic review and meta-analysis. *Physical Therapy Reviews* 2016:1-13. doi: 10.1080/10833196.2016.1263415
7. Pearson M, Dieberg G, Smart N. Exercise as a therapy for improvement of walking ability in adults with multiple sclerosis: a meta-analysis. *Arch Phys Med Rehabil* 2015;96(7):1339-48.
8. Latimer-Cheung AE, Martin Ginis KA, Hicks AL, et al. Development of evidence-informed physical activity guidelines for adults with multiple sclerosis. *Arch Phys Med Rehabil* 2013;94(9):1829-36.

For peer review only - http://bmjopen.bmj.com/site/about/guidelines.xhtml

9. Garrett M, Hogan N, Larkin A, et al. Exercise in the community for people with minimal gait impairment due to MS: an assessor-blind randomized controlled trial. *Mult Scler* 2013;19(6):782-9.
10. Garrett M, Hogan N, Larkin A, et al. Exercise in the community for people with multiple sclerosis--a follow-up of people with minimal gait impairment. *Mult Scler* 2013;19(6):790-8.
11. Michie S, van Stralen MM, West R. The behaviour change wheel: a new method for characterising and designing behaviour change interventions. *Implementation Science* 2011;6(1):42.
12. Dlugonski D, Wojcicki TR, McAuley E, et al. Social cognitive correlates of physical activity in inactive adults with multiple sclerosis. *Int J Rehabil Res* 2011;34(2):115-20.
13. Motl RW, Dlugonski D, Wojcicki TR, et al. Internet intervention for increasing physical activity in persons with multiple sclerosis. *Mult Scler* 2011;17(1):116-28.
14. Dlugonski D, Motl RW, Mohr DC, et al. Internet-delivered behavioral intervention to increase physical activity in persons with multiple sclerosis: Sustainability and secondary outcomes. *Psychology, Health & Medicine* 2012;17(6):636-51.
15. McAuley E, Courneya Ks Fau - Rudolph DL, Rudolph Dl Fau - Lox CL, et al. Enhancing exercise adherence in middle-aged males and females. (0091-7435 (Print))
16. McAuley E, Motl Rw Fau - Morris KS, Morris Ks Fau - Hu L, et al. Enhancing physical activity adherence and well-being in multiple sclerosis: a randomised controlled trial. (1352-4585 (Print))
17. Coote S, Gallagher S, Msetfi R, et al. A randomised controlled trial of an exercise plus behaviour change intervention in people with multiple sclerosis: the step it up study protocol. *BMC Neurol* 2014;14(241):014-0241.
18. Hadjimichael O, Kerns RD, Rizzo MA, et al. Persistent pain and uncomfortable sensations in persons with multiple sclerosis. *Pain* 2007;127(1-2):35-41.
19. Goldman MD, Motl RW, Rudick RA. Possible clinical outcome measures for clinical trials in patients with multiple sclerosis. *Ther Adv Neurol Disord* 2010;3(4):229-39. doi: 10.1177/1756285610374117 [published Online First: 2010/12/24]
20. Fry DK, Pfalzer LA. Reliability of four functional tests and rating of perceived exertion in persons with multiple sclerosis. *PHYSIOTHERAPY CANADA* 2006;58(3):212-20.
21. Nilsagard Y, Lundholm C, Gunnarsson L-G, et al. Clinical relevance using timed walk tests and'timed up and go'testing in persons with multiple sclerosis. *Physiotherapy Research International* 2007;12(2):105-14.
22. Hobart J, Riazi A, Lamping D, et al. Measuring the impact of MS on walking ability The 12-Item MS Walking Scale (MSWS-12). *Neurology* 2003;60(1):31-36.
23. McGuigan C, Hutchinson M. The multiple sclerosis impact scale (MSIS-29) is a reliable and sensitive measure. *Journal of neurology, neurosurgery, and psychiatry* 2004;75(2):266-69.
24. Motl RW, McAuley E, Mullen S. Longitudinal measurement invariance of the multiple sclerosis walking scale-12. *Journal Of The Neurological Sciences* 2011;305(1):75-79.
25. Cavanaugh JT, Gappmaier VO, Dibble LE, et al. Ambulatory activity in individuals with multiple sclerosis. *Journal of Neurologic Physical Therapy* 2011;35(1):26-33.
26. Csuka M, McCarty DJ. Simple method for measurement of lower extremity muscle strength. *Am J Med* 1985;78(1):77-81.
27. Kuspinar A, Andersen RE, Teng SY, et al. Predicting exercise capacity through submaximal fitness tests in persons with multiple sclerosis. *Archives Of Physical Medicine And Rehabilitation* 2010;91(9):1410-17.
28. Godin G, Shephard RJ. A simple method to assess exercise behavior in the community. *Can J Appl Sport Sci* 1985;10(3):141-6.
29. Baert I, Freeman J, Smedal T, et al. Responsiveness and clinically meaningful improvement, according to disability level, of five walking measures after rehabilitation in multiple sclerosis

For peer review only - http://bmjopen.bmj.com/site/about/guidelines.xhtml

a European multicenter study. *Neurorehabilitation and neural repair* 2014:1545968314521010.
30. Hedges LV. Advances in statistical methods for meta-analysis. *New Directions for Evaluation* 1984;1984(24):25-42.
31. Stubbs B, Vancampfort D, Rosenbaum S, et al. Dropout from exercise randomized controlled trials among people with depression: A meta-analysis and meta regression. *Journal of affective disorders* 2016;190:457-66.
32. Pilutti LA, Platta ME, Motl RW, et al. The safety of exercise training in multiple sclerosis: A systematic review. *Journal Of The Neurological Sciences* 2014
33. Carter A, Daley A, Humphreys L, et al. Pragmatic intervention for increasing self-directed exercise behaviour and improving important health outcomes in people with multiple sclerosis: a randomised controlled trial. (1477-0970 (Electronic))
34. Learmonth YC, Paul L Fau - Miller L, Miller L Fau - Mattison P, et al. The effects of a 12-week leisure centre-based, group exercise intervention for people moderately affected with multiple sclerosis: a randomized controlled pilot study. (1477-0873 (Electronic))
35. Casanova C, Celli B, Barria P, et al. The 6-min walk distance in healthy subjects: reference standards from seven countries. *European Respiratory Journal* 2011;37(1):150-56.
36. Casey B, Coote S, Shirazipour C, et al. Modifiable Psychosocial Constructs Associated with Physical Activity Participation in People with Multiple Sclerosis: A Systematic Review and Meta-Analysis. *Archives of Physical Medicine and Rehabilitation* 2017;in press

For peer review only

Figure 1 *CONSORT Flow Diagram,*

DNA: did not attend

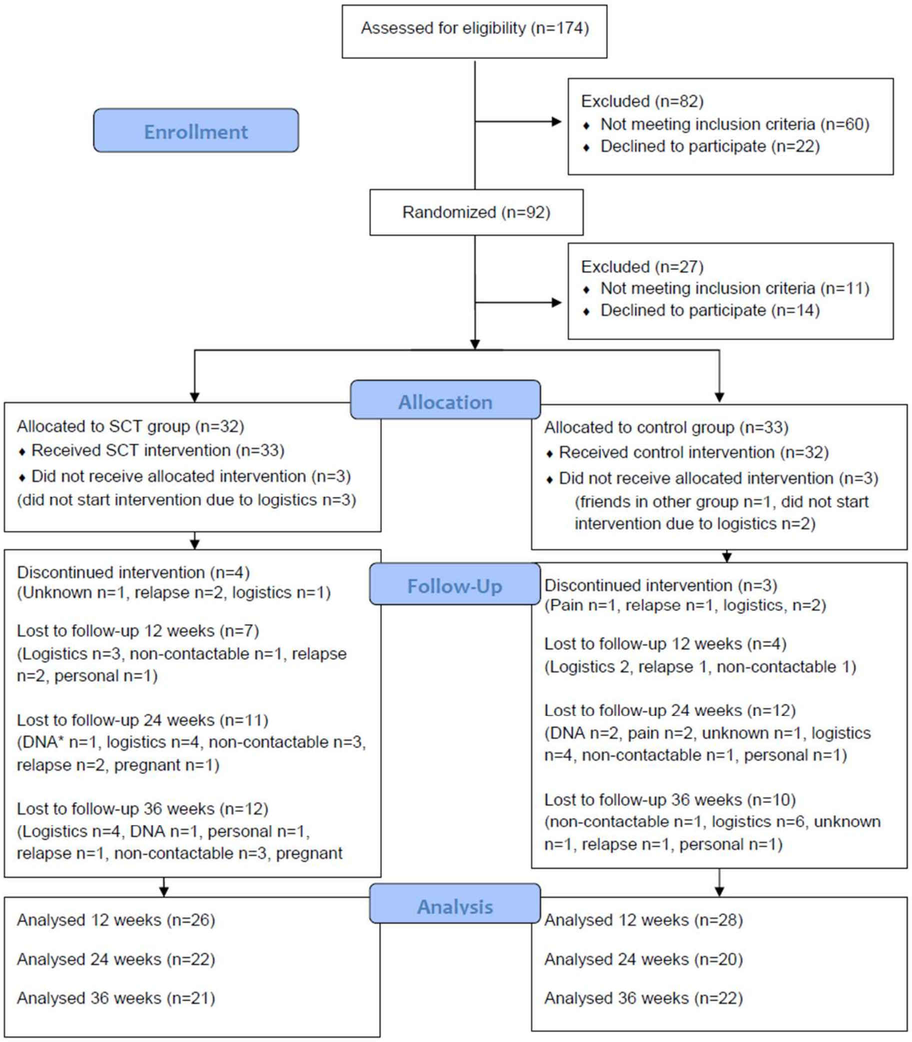

Figure 1 CONSORT Flow Diagram

331x426mm (72 x 72 DPI)

Figure 2 Proportion of participants completing sessions (Exercise Diary data).
SCT = exercise plus social cognitive theory education group, CON = exercise plus contact control education

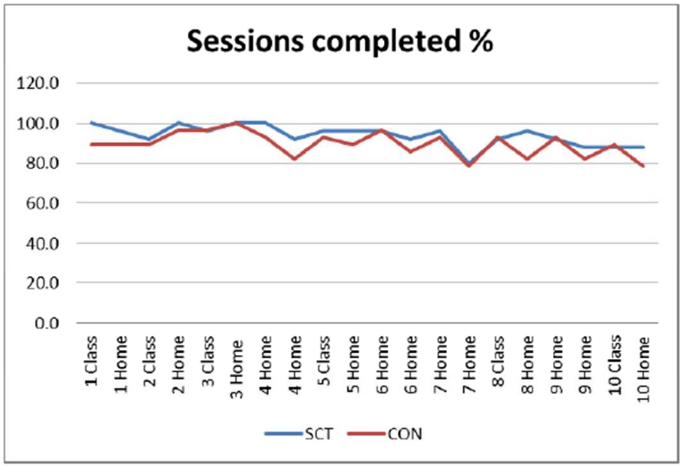

Figure 2 Proportion of participants completing sessions (Exercise Diary data).

277x171mm (72 x 72 DPI)

Figure 3 – Estimated effects on primary and secondary measures using intention to treat and secondary analysis

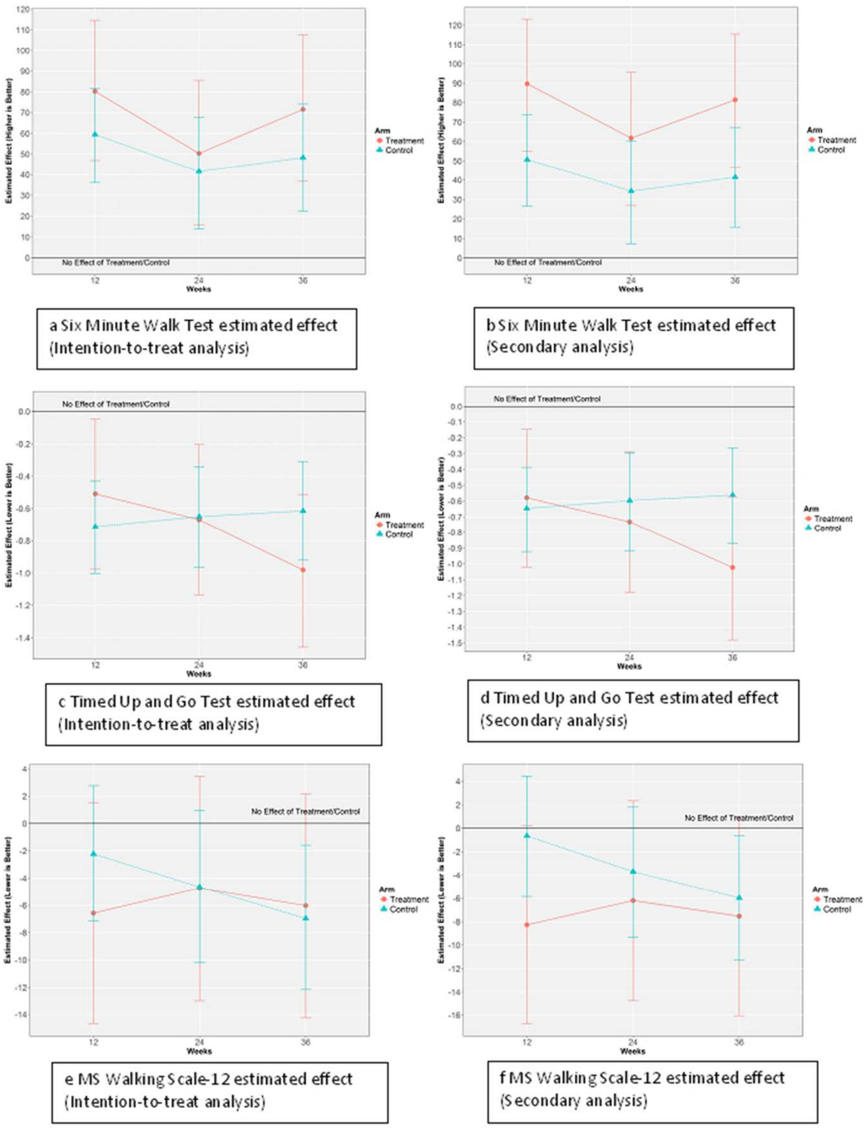

Figure 3 – Estimated effects using intention to treat and secondary analysis

244x337mm (72 x 72 DPI)

TIDieR Template for Intervention Description and Replication

The TIDieR (Template for Intervention Description and Replication) Checklist*:

Information to include when describing an intervention and the location of the information

Item number	Item	Where located **	
		Primary paper (page or appendix number)	Other † (details)
	**BRIEF NAME**		
**1.**	Provide the name or a phrase that describes the intervention.	1	Title; Exercise plus behaviour change intervention
	**WHY**		
**2.**	Describe any rationale, theory, or goal of the elements essential to the intervention.	5,6	We designed a randomised controlled pilot trial called 'Step it Up' that combined a group exercise programme with a theory-based education component for augmenting the effect of exercise on walking outcomes and sustaining these changes over time. We compared SCT based education to attention control education on topics unrelated to exercise. SCT was used to develop the content of the educational element as it has been widely investigated and associated with PA behaviour in people with MS
	**WHAT**		
**3.**	Materials: Describe any physical or informational materials used in the intervention, including those provided to participants or used in intervention delivery or in training of intervention providers. Provide information on where the	9 Protocol paper page 3, 4 https://bmcneu	The exercise log book and exercise pictures are available as an online appendix

TIDieR checklist

	materials can be accessed (e.g. online appendix, URL).	rol.biomedcentral.com/articles/10.1186/s12883-014-0241-9	
**4.**	Procedures: Describe each of the procedures, activities, and/or processes used in the intervention, including any enabling or support activities.	9 Protocol paper page 3	Over the 10-week programme participants attended the group exercise class on six occasions, supplemented with a telephone coaching call in the weeks without classes (intervention weeks 4, 6, 7 and 9). After each of the group exercise classes both groups received an education session and the content is described in the protocol paper
	**WHO PROVIDED**		
**5.**	For each category of intervention provider (e.g. psychologist, nursing assistant), describe their expertise, background and any specific training given.	10	The physiotherapists who delivered the intervention or control group sessions were provided with a one-day training course on the delivery of the intervention for their group.
	**HOW**		
**6.**	Describe the modes of delivery (e.g. face-to-face or by some other mechanism, such as internet or telephone) of the intervention and whether it was provided individually or in a group.	9	There were 44 sessions over 10 weeks; 6 group strengthening classes followed by education, 14 home strengthening classes, 20 home walking sessions and 4 telephone calls
	**WHERE**		
**7.**	Describe the type(s) of location(s) where the intervention occurred, including any necessary infrastructure or relevant features.	9	Recruitment and interventions took place in Cork Galway and Limerick Ireland. All classes happened at community venues, the other sessions were home based.
	**WHEN and HOW MUCH**		
**8.**	Describe the number of times the intervention was delivered	See 6 above	The target walking exercise intensity

TIDieR checklist

For peer review only

	and over what period of time including the number of sessions, their schedule, and their duration, intensity or dose.	Protocol paper page 3 describes intensity of waling and strengthening sessions.	for both groups in the current study was at a rate of 100 steps per minute. Participants started with 10 minutes of walking twice weekly at a rate of 100 steps/minute and increased incrementally in 5 minute intervals over 5 weeks wherein they aimed to reach the guideline of30 minutes twice weekly The intensity and duration of the strengthening component of the intervention was progressed by increasing the number of repetitions and sets and changing the resistance of the elastic resistance band used for each strengthening exercise. Participants started with one set of 10–15 repetitions and gradually increased the number of sets, repetitions and level of resistance until they meet the target of two sets of each exercise twice weekly with sufficient resistance that they are failing on the 12th repetition.
	**TAILORING**		
**9.**	If the intervention was planned to be personalised, titrated or adapted, then describe what, why, when, and how.	Protocol paper page 3	Intensity was personalised based on each participants ability/performance of resistance and aerobic exercise Progression through the programme was based on individual performance in the previous session.
	**MODIFICATIONS**		
**10.**‡	If the intervention was modified during the course of the study, describe the changes (what, why, when, and how).	13, 14	Not all participants met the guideline target by week 6. The proportion of participants in each group reaching the guideline and reasons for not reaching guideline are described in the results

For peer review only - http://bmjopen.bmj.com/site/about/guidelines.xhtml

	**HOW WELL**		
**11.**	Planned: If intervention adherence or fidelity was assessed, describe how and by whom, and if any strategies were used to maintain or improve fidelity, describe them.	12, Protocol paper page 4	Exercise logs, video or/audio recording of sessions and independent evaluation of those recorded sessions were utilised to evaluate fidelity
**12.‡**	Actual: If intervention adherence or fidelity was assessed, describe the extent to which the intervention was delivered as planned.	13,14	Adherence to the programme evaluated using the exercise logs<br>Fidelity was assessed by an independent person using the video/audio recordings

** **Authors** - use N/A if an item is not applicable for the intervention being described. **Reviewers** – use '?' if information about the element is not reported/not sufficiently reported.

† If the information is not provided in the primary paper, give details of where this information is available. This may include locations such as a published protocol or other published papers (provide citation details) or a website (provide the URL).

‡ If completing the TIDieR checklist for a protocol, these items are not relevant to the protocol and cannot be described until the study is complete.

* We strongly recommend using this checklist in conjunction with the TIDieR guide (see *BMJ* 2014;348:g1687) which contains an explanation and elaboration for each item.

* The focus of TIDieR is on reporting details of the intervention elements (and where relevant, comparison elements) of a study. Other elements and methodological features of studies are covered by other reporting statements and checklists and have not been duplicated as part of the TIDieR checklist. When a **randomised trial** is being reported, the TIDieR checklist should be used in conjunction with the CONSORT statement (see www.consort-statement.org) as an extension of **Item 5 of the CONSORT 2010 Statement.** When a **clinical trial protocol** is being reported, the TIDieR checklist should be used in conjunction with the SPIRIT statement as an extension of **Item 11 of the SPIRIT 2013 Statement** (see www.spirit-statement.org). For alternate study designs, TIDieR can be used in conjunction with the appropriate checklist for that study design (see www.equator-network.org).

TIDieR checklist

For peer review only

Your Step it Up exercise Log book

Please use this log book to record **BOTH** the strengthening exercises and the walking exercise that you do throughout this programme. This log book is to be used **both during your exercise classes** with your physiotherapist and **while you are doing your exercises at home**. The reason we ask you to complete this log is so you can keep a reliable record of progress that you are making with the Step it Up programme. It is also important for the research team at UL to track your progress with the programme.

Each week you should fill the relevant table. As you can see, in the last 2 columns of each of the tables- we have asked you to rate your "BORG score" and your "enjoyment score"- at the end of this document you will see instructions on how to complete these ratings.

If you have any questions regarding filling this log book out please do not hesitate to contact Dr. Susan Coote (susan.coote@ul.ie) or the physiotherapist who is delivering your programme. When the 10-week exercise class is finished, please give your log book to the physiotherapist who completes your follow-up assessment (Dr. Sara Hayes). You will be given a date for this assessment closer to the time.

© University of Limerick/University of Illinois, Urbana-Champaign 2014. This information is being provided solely for the purpose of delivering this intervention as part of the HRB funded Step it Up Trial and should not to be reproduced without permission or used for any other purpose.

For peer review only - http://bmjopen.bmj.com/site/about/guidelines.xhtml

Week 1

Date	Strengthening exercises										BORG score (6-20)	Enjoyment score (1-7)
**Class**	**Knee bend**	**Squat**	**Shoulder bend-forward**	**Shoulder bend-sideways**	**Elbow bend**	**Lunge**	**Hip bend-backward**	**Hip bend-sideways**	**Heel raises-standing**	**Ankle bend-sitting**		
	Reps	Reps	Reps	Reps	Reps	Reps	Reps	Reps	Reps	Reps		
	Sets	Sets	Sets	Sets	Sets	Sets	Sets	Sets	Sets	Sets		
	Colour	Colour	Colour	Colour	Colour	Colour	Colour	Colour	Colour	Colour		
**Home**	**Knee bend**	**Squat**	**Shoulder bend-forward**	**Shoulder bends-sideways**	**Elbow bend**	**Lunge**	**Hip bend-backward**	**Hip bends-sideways**	**Heel raises-standing**	**Ankle bend-sitting**		
	Reps	Reps	Reps	Reps	Reps	Reps	Reps	Reps	Reps	Reps		
	Sets	Sets	Sets	Sets	Sets	Sets	Sets	Sets	Sets	Sets		
	Colour	Colour	Colour	Colour	Colour	Colour	Colour	Colour	Colour	Colour		
	**Walking exercise**											
**Home**	**Time spent walking (minutes):**				**Number of steps:**							
**Home**	**Time spent walking (minutes):**				**Number of steps:**							

For peer review only

Week 2

Date	Strengthening exercises										BORG score (6-20)	Enjoyment score (1-7)
**Class**	**Knee bend**	**Squat**	**Shoulder bend-forward**	**Shoulder bends-sideways**	**Elbow bend**	**Lunge**	**Hip bend-backward**	**Hip bend-sideways**	**Heel raises-standing**	**Ankle bend-sitting**		
	Reps	Reps	Reps	Reps	Reps	Reps	Reps	Reps	Reps	Reps		
	Sets	Sets	Sets	Sets	Sets	Sets	Sets	Sets	Sets	Sets		
	Colour	Colour	Colour	Colour	Colour	Colour	Colour	Colour	Colour	Colour		
**Home**	**Knee bend**	**Squat**	**Shoulder bend-forward**	**Shoulder bends-sideways**	**Elbow bend**	**Lunge**	**Hip bend-backward**	**Hip bends-sideways**	**Heel raises-standing**	**Ankle bend-sitting**		
	Reps	Reps	Reps	Reps	Reps	Reps	Reps	Reps	Reps	Reps		
	Sets	Sets	Sets	Sets	Sets	Sets	Sets	Sets	Sets	Sets		
	Colour	Colour	Colour	Colour	Colour	Colour	Colour	Colour	Colour	Colour		
	**Walking exercise**											
**Home**	**Time spent walking (minutes):**					**Number of steps:**						
**Home**	**Time spent walking (minutes):**					**Number of steps:**						

For peer review only

Week 3

Date	Strengthening exercises										BORG score (6-20)	Enjoyment score (1-7)
Class	Knee bend	Squat	Shoulder bend-forward	Shoulder bends-sideways	Elbow bend	Lunge	Hip bend-backward	Hip bend-sideways	Heel raises-standing	Ankle bend-sitting		
	Reps	Reps	Reps	Reps	Reps	Reps	Reps	Reps	Reps	Reps		
	Sets	Sets	Sets	Sets	Sets	Sets	Sets	Sets	Sets	Sets		
	Colour	Colour	Colour	Colour	Colour	Colour	Colour	Colour	Colour	Colour		
Home	Knee bend	Squat	Shoulder bend-forward	Shoulder bends-sideways	Elbow bend	Lunge	Hip bend-backward	Hip bend-sideways	Heel raises-standing	Ankle bend-sitting		
	Reps	Reps	Reps	Reps	Reps	Reps	Reps	Reps	Reps	Reps		
	Sets	Sets	Sets	Sets	Sets	Sets	Sets	Sets	Sets	Sets		
	Colour	Colour	Colour	Colour	Colour	Colour	Colour	Colour	Colour	Colour		
	Walking exercise											
Home	Time spent walking (minutes):				Number of steps:							
Home	Time spent walking (minutes):				Number of steps:							

For peer review only

Week 4

Date	Strengthening exercises										BORG score (6-20)	Enjoyment score (1-7)
**Home**	**Knee bend**	**Squat**	**Shoulder bend-forward**	**Shoulder bends-sideways**	**Elbow bend**	**Lunge**	**Hip bend-backward**	**Hip bends-sideways**	**Heel raises-standing**	**Ankle bend-sitting**		
	Reps	Reps	Reps	Reps	Reps	Reps	Reps	Reps	Reps	Reps		
	Sets	Sets	Sets	Sets	Sets	Sets	Sets	Sets	Sets	Sets		
	Colour	Colour	Colour	Colour	Colour	Colour	Colour	Colour	Colour	Colour		
**Home**	**Knee bend**	**Squat**	**Shoulder bend-forward**	**Shoulder bends-sideways**	**Elbow bend**	**Lunge**	**Hip bend-backward**	**Hip bends-sideways**	**Heel raises-standing**	**Ankle bend-sitting**		
	Reps	Reps	Reps	Reps	Reps	Reps	Reps	Reps	Reps	Reps		
	Sets	Sets	Sets	Sets	Sets	Sets	Sets	Sets	Sets	Sets		
	Colour	Colour	Colour	Colour	Colour	Colour	Colour	Colour	Colour	Colour		
	**Walking exercise**											
**Home**	**Time spent walking (minutes):**				**Number of steps:**							
**Home**	**Time spent walking (minutes):**				**Number of steps:**							

For peer review only

Week 5

Date	Strengthening exercises										BORG score (6-20)	Enjoyment score (1-7)
Class	Knee bend	Squat	Shoulder bend-forward	Shoulder bends-sideways	Elbow bend	Lunge	Hip bend-backward	Hip bends-sideways	Heel raises-standing	Ankle bend-sitting		
	Reps	Reps	Reps	Reps	Reps	Reps	Reps	Reps	Reps	Reps		
	Sets	Sets	Sets	Sets	Sets	Sets	Sets	Sets	Sets	Sets		
	Colour	Colour	Colour	Colour	Colour	Colour	Colour	Colour	Colour	Colour		
Home	Knee bend	Squat	Shoulder bend-forward	Shoulder bends-sideways	Elbow bend	Lunge	Hip bend-backward	Hip bend-sideways	Heel raises-standing	Ankle bend-sitting		
	Reps	Reps	Reps	Reps	Reps	Reps	Reps	Reps	Reps	Reps		
	Sets	Sets	Sets	Sets	Sets	Sets	Sets	Sets	Sets	Sets		
	Colour	Colour	Colour	Colour	Colour	Colour	Colour	Colour	Colour	Colour		
	Walking exercise											
Home	Time spent walking (minutes):				Number of steps:							
Home	Time spent walking (minutes):				Number of steps:							

For peer review only

Week 6

Date	Strengthening exercises										BORG score (6-20)	Enjoyment score (1-7)
Home	**Knee bend**	**Squat**	**Shoulder bend-forward**	**Shoulder bends-sideways**	**Elbow bend**	**Lunge**	**Hip bend-backward**	**Hip bend-sideways**	**Heel raises-standing**	**Ankle bend-sitting**		
	Reps	Reps	Reps	Reps	Reps	Reps	Reps	Reps	Reps	Reps		
	Sets	Sets	Sets	Sets	Sets	Sets	Sets	Sets	Sets	Sets		
	Colour	Colour	Colour	Colour	Colour	Colour	Colour	Colour	Colour	Colour		
Home	**Knee bend**	**Squat**	**Shoulder bend-forward**	**Shoulder bends-sideways**	**Elbow bend**	**Lunge**	**Hip bend-backward**	**Hip bend-sideways**	**Heel raises-standing**	**Ankle bend-sitting**		
	Reps	Reps	Reps	Reps	Reps	Reps	Reps	Reps	Reps	Reps		
	Sets	Sets	Sets	Sets	Sets	Sets	Sets	Sets	Sets	Sets		
	Colour	Colour	Colour	Colour	Colour	Colour	Colour	Colour	Colour	Colour		
	**Walking exercise**											
Home	**Time spent walking (minutes):**				**Number of steps:**							
Home	**Time spent walking (minutes):**				**Number of steps:**							

For peer review only

For peer review only - http://bmjopen.bmj.com/site/about/guidelines.xhtml

Week 7

Date	Strengthening exercises										BORG score (6-20)	Enjoyment score (1-7)
Home	**Knee bend**	**Squat**	**Shoulder bend-forward**	**Shoulder bends-sideways**	**Elbow bend**	**Lunge**	**Hip bend-backward**	**Hip bend-sideways**	**Heel raises-standing**	**Ankle bend-sitting**		
	Reps	Reps	Reps	Reps	Reps	Reps	Reps	Reps	Reps	Reps		
	Sets	Sets	Sets	Sets	Sets	Sets	Sets	Sets	Sets	Sets		
	Colour	Colour	Colour	Colour	Colour	Colour	Colour	Colour	Colour	Colour		
Home	**Knee bend**	**Squat**	**Shoulder bend-forward**	**Shoulder bends-sideways**	**Elbow bend**	**Lunge**	**Hip bend-backward**	**Hip bend-sideways**	**Heel raises-standing**	**Ankle bend-sitting**		
	Reps	Reps	Reps	Reps	Reps	Reps	Reps	Reps	Reps	Reps		
	Sets	Sets	Sets	Sets	Sets	Sets	Sets	Sets	Sets	Sets		
	Colour	Colour	Colour	Colour	Colour	Colour	Colour	Colour	Colour	Colour		
	**Walking exercise**											
Home	**Time spent walking (minutes):**				**Number of steps:**							
Home	**Time spent walking (minutes):**				**Number of steps:**							

For peer review only

Week 8

Date	Strengthening exercises										BORG score (6-20)	Enjoyment score (1-7)
Class	Knee bend	Squat	Shoulder bend-forward	Shoulder bends-sideways	Elbow bend	Lunge	Hip bend-backward	Hip bend-sideways	Heel raises-standing	Ankle bend-sitting		
	Reps	Reps	Reps	Reps	Reps	Reps	Reps	Reps	Reps	Reps		
	Sets	Sets	Sets	Sets	Sets	Sets	Sets	Sets	Sets	Sets		
	Colour	Colour	Colour	Colour	Colour	Colour	Colour	Colour	Colour	Colour		
Home	Knee bend	Squat	Shoulder bend-forward	Shoulder bends-sideways	Elbow bend	Lunge	Hip bend-backward	Hip bend-sideways	Heel raises-standing	Ankle bend-sitting		
	Reps	Reps	Reps	Reps	Reps	Reps	Reps	Reps	Reps	Reps		
	Sets	Sets	Sets	Sets	Sets	Sets	Sets	Sets	Sets	Sets		
	Colour	Colour	Colour	Colour	Colour	Colour	Colour	Colour	Colour	Colour		
	**Walking exercise**											
Home	Time spent walking (minutes):				Number of steps:							
Home	Time spent walking (minutes):				Number of steps:							

For peer review only

Week 9

Date	Strengthening exercises										BORG score (6-20)	Enjoyment score (1-7)
**Home**	**Knee bend**	**Squat**	**Shoulder bend-forward**	**Shoulder bends-sideways**	**Elbow bend**	**Lunge**	**Hip bend-backward**	**Hip bend-sideways**	**Heel raises-standing**	**Ankle bend-sitting**		
	Reps	Reps	Reps	Reps	Reps	Reps	Reps	Reps	Reps	Reps		
	Sets	Sets	Sets	Sets	Sets	Sets	Sets	Sets	Sets	Sets		
	Colour	Colour	Colour	Colour	Colour	Colour	Colour	Colour	Colour	Colour		
**Home**	**Knee bend**	**Squat**	**Shoulder bend-forward**	**Shoulder bends-sideways**	**Elbow bend**	**Lunge**	**Hip bend-backward**	**Hip bend-sideways**	**Heel raises-standing**	**Ankle bend-sitting**		
	Reps	Reps	Reps	Reps	Reps	Reps	Reps	Reps	Reps	Reps		
	Sets	Sets	Sets	Sets	Sets	Sets	Sets	Sets	Sets	Sets		
	Colour	Colour	Colour	Colour	Colour	Colour	Colour	Colour	Colour	Colour		
	**Walking exercise**											
**Home**	**Time spent walking (minutes):**				**Number of steps:**							
**Home**	**Time spent walking (minutes):**				**Number of steps:**							

Week 10

Date	Strengthening exercises										BORG score (6-20)	Enjoyment score (1-7)
**Class**	**Knee bend**	**Squat**	**Shoulder bend-forward**	**Shoulder bends-sideways**	**Elbow bend**	**Lunge**	**Hip bend-backward**	**Hip bend-sideways**	**Heel raises-standing**	**Ankle bend-sitting**		
	Reps	Reps	Reps	Reps	Reps	Reps	Reps	Reps	Reps	Reps		
	Sets	Sets	Sets	Sets	Sets	Sets	Sets	Sets	Sets	Sets		
	Colour	Colour	Colour	Colour	Colour	Colour	Colour	Colour	Colour	Colour		
**Home**	**Knee bend**	**Squat**	**Shoulder bend-forward**	**Shoulder bends-sideways**	**Elbow bend**	**Lunge**	**Hip bend-backward**	**Hip bend-sideways**	**Heel raises-standing**	**Ankle bend-sitting**		
	Reps	Reps	Reps	Reps	Reps	Reps	Reps	Reps	Reps	Reps		
	Sets	Sets	Sets	Sets	Sets	Sets	Sets	Sets	Sets	Sets		
	Colour	Colour	Colour	Colour	Colour	Colour	Colour	Colour	Colour	Colour		
	**Walking exercise**											
**Home**	**Time spent walking (minutes):**					**Number of steps:**						
**Home**	**Time spent walking (minutes):**					**Number of steps:**						

For peer review only

For peer review only - http://bmjopen.bmj.com/site/about/guidelines.xhtml

***BORG scoring instructions**

While doing physical activity, we want you to rate your perception of exertion. This feeling should reflect how heavy and strenuous the exercise feels to you, Combining all sensations and feelings of physical stress, effort, and fatigue. Do not concern yourself with any one factor such as leg pain or shortness of breath, but try to focus on your total feeling of exertion.

Look at the rating scale below while you are engaging in an activity; it ranges from 6 to 20, where 6 means "no exertion at all" and 20 means "maximal exertion." Choose the number from below that best describes your level of exertion. This will give you a good idea of the intensity level of your activity, and you can use this information to speed up or slow down your movements to reach your desired range.

Try to appraise your feeling of exertion as honestly as possible, without thinking about what the actual physical load is. Your own feeling of effort and exertion is important, not how it compares to other people's. Look at the scales and the expressions and then give a number.

---

6 No exertion at all

7 Extremely light

8

9 Very light - (easy walking slowly at a comfortable pace)

10

11 Light

12

13 Somewhat hard (It is quite an effort; you feel tired but can continue)

14

15 Hard (heavy)

16

17 Very hard (very strenuous, and you are very fatigued)

18

19 Extremely hard (You cannot continue for long at this pace)

20 Maximal exertion

© University of Limerick/University of Illinois, Urbana-Champaign 2014. This information is being provided solely for the purpose of delivering this intervention as part of the HRB funded Step it Trial and should not to be reproduced without permission or used for any other purpose.

For peer review only

** Your Enjoyment Scale Score

Enjoyment Scale<br>"How much did you enjoy your exercise session today?"						
1	2	3	4	5	6	7
not at all			somewhat			very much

For peer review only

© University of Limerick/University of Illinois, Urbana-Champaign 2014. This information is being provided solely for the purpose of delivering this intervention as part of the HRB funded Step it Trial and should not to be reproduced without permission or used for any other purpose.

For peer review only

For peer review only - http://bmjopen.bmj.com/site/about/guidelines.xhtml

Knee bend

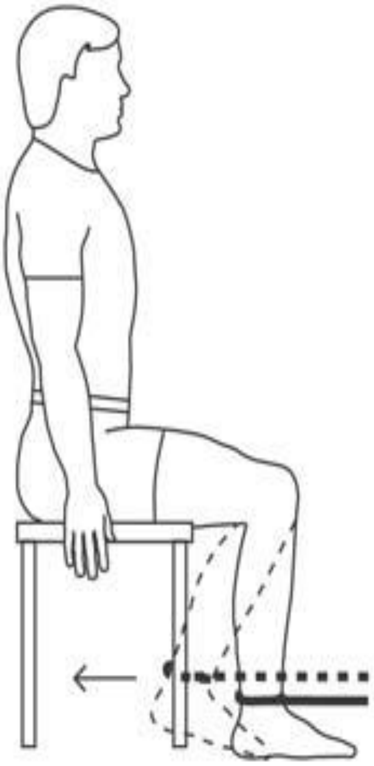

Squat

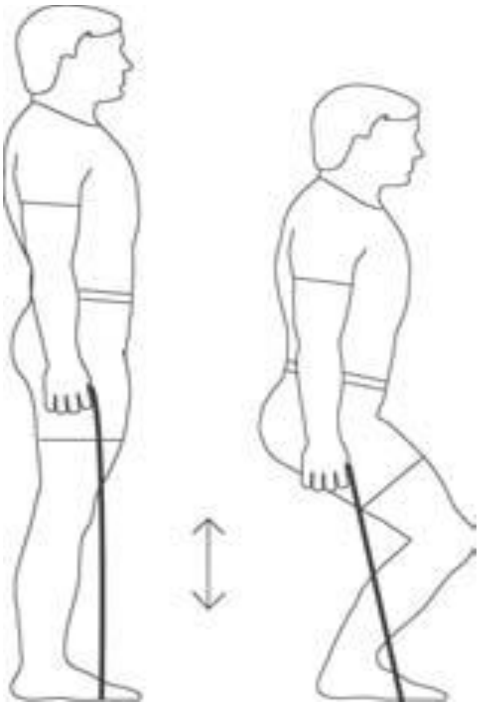

For peer review only - http://bmjopen.bmj.com/site/about/guidelines.xhtml

Shoulder bend- forward

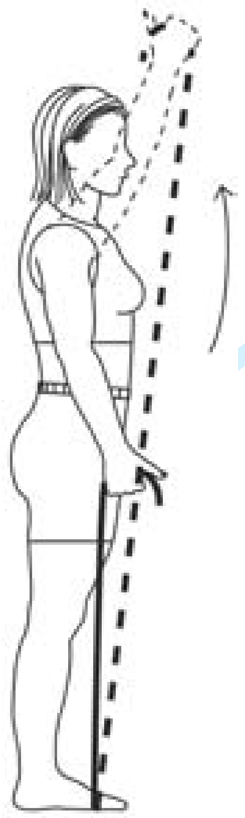

Shoulder bend-sideways

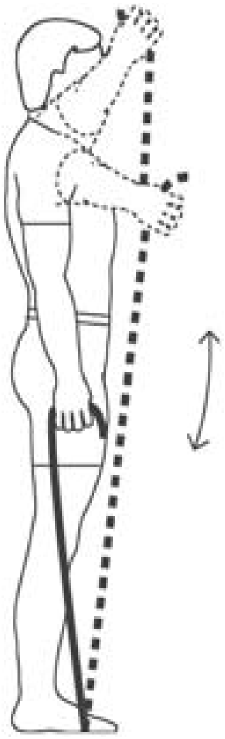

For peer review only

Elbow bend

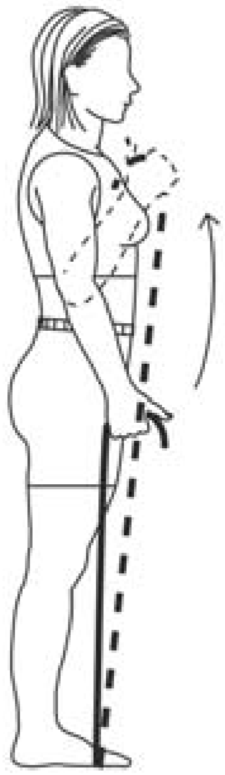

Lunge

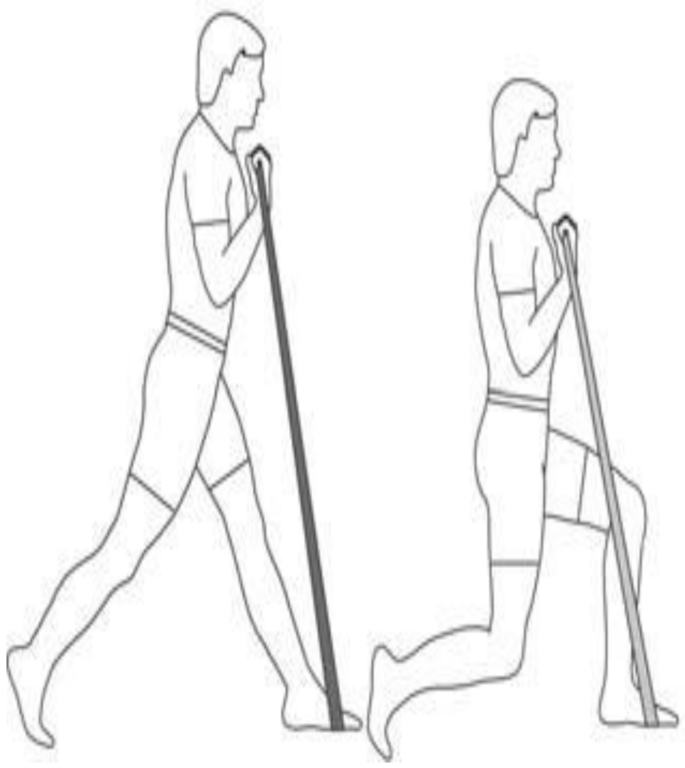

For peer review only

Hip bend- backward

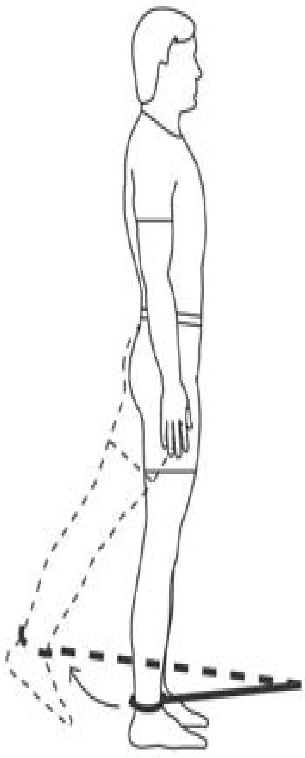

Hip bend- sideways

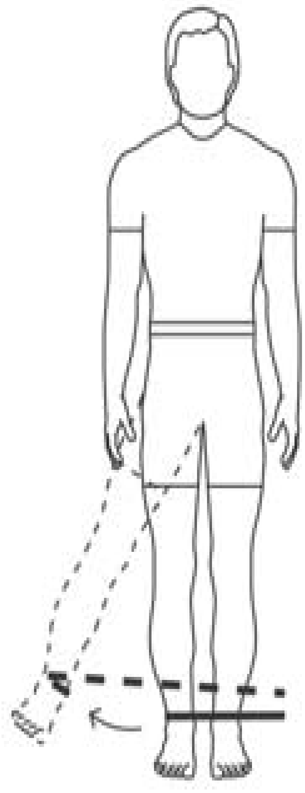

For peer review only

Heel raises- standing

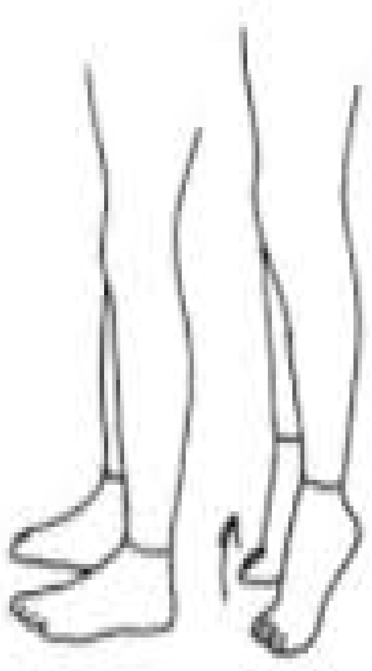

Ankle bend- sitting

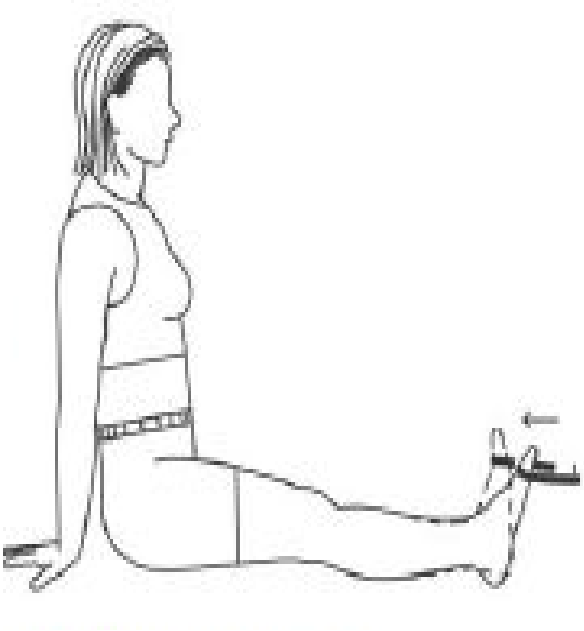

For peer review only

CONSORT checklist of information to include when reporting a pilot trial*

Section/topic and item No	Standard checklist item	Extension for pilot trials	Page No where item is reported
**Title and abstract**			
1a	Identification as a randomised trial in the title	Identification as a pilot or feasibility randomised trial in the title	1
1b	Structured summary of trial design, methods, results, and conclusions (for specific guidance see CONSORT for abstracts)	Structured summary of pilot trial design, methods, results, and conclusions (for specific guidance see CONSORT abstract extension for pilot trials)	3
**Introduction**			
Background and objectives:			
2a	Scientific background and explanation of rationale	Scientific background and explanation of rationale for future definitive trial, and reasons for randomised pilot trial	5
2b	Specific objectives or hypotheses	Specific objectives or research questions for pilot trial	5-6
**Methods**			
Trial design:			
3a	Description of trial design (such as parallel, factorial) including allocation ratio	Description of pilot trial design (such as parallel, factorial) including allocation ratio	6
3b	Important changes to methods after trial commencement (such as eligibility criteria), with reasons	Important changes to methods after pilot trial commencement (such as eligibility criteria), with reasons	10
Participants:			
4a	Eligibility criteria for participants		
4b	Settings and locations where the data were collected		
4c		How participants were identified and consented	6
Interventions:			
5	The interventions for each group with sufficient details to allow replication, including how and when they were actually administered		
Outcomes:			
6a	Completely defined prespecified primary and secondary outcome measures, including how and when they were assessed	Completely defined prespecified assessments or measurements to address each pilot trial objective specified in 2b, including how and when they were assessed	7
6b	Any changes to trial outcomes after the trial commenced, with reasons	Any changes to pilot trial assessments or measurements after the pilot trial commenced, with reasons	N/A
6c		If applicable, prespecified criteria used to judge whether, or how, to proceed with future definitive trial	N/A

Sample size:			
7a	How sample size was determined	Rationale for numbers in the pilot trial	10
7b	When applicable, explanation of any interim analyses and stopping guidelines		
Randomisation:			
Sequence generation:			
8a	Method used to generate the random allocation sequence		
8b	Type of randomisation; details of any restriction (such as blocking and block size)	Type of randomisation(s); details of any restriction (such as blocking and block size)	7
Allocation concealment mechanism:			
9	Mechanism used to implement the random allocation sequence (such as sequentially numbered containers), describing any steps taken to conceal the sequence until interventions were assigned		
Implementation:			
10	Who generated the random allocation sequence, enrolled participants, and assigned participants to interventions		
Blinding:			
11a	If done, who was blinded after assignment to interventions (eg, participants, care providers, those assessing outcomes) and how		
11b	If relevant, description of the similarity of interventions		
Analytical methods:			
12a	Statistical methods used to compare groups for primary and secondary outcomes	Methods used to address each pilot trial objective whether qualitative or quantitative	10, 11
12b	Methods for additional analyses, such as subgroup analyses and adjusted analyses	Not applicable	
**Results**			
Participant flow (a diagram is strongly recommended):			
13a	For each group, the numbers of participants who were randomly assigned, received intended treatment, and were analysed for the primary outcome	For each group, the numbers of participants who were approached and/or assessed for eligibility, randomly assigned, received intended treatment, and were assessed for each objective	Fig 1, p11
13b	For each group, losses and exclusions after randomisation, together with reasons		
Recruitment:			

14a	Dates defining the periods of recruitment and follow-up		
14b	Why the trial ended or was stopped	Why the pilot trial ended or was stopped	10
Baseline data:			
15	A table showing baseline demographic and clinical characteristics for each group		
Numbers analysed:			
16	For each group, number of participants (denominator) included in each analysis and whether the analysis was by original assigned groups	For each objective, number of participants (denominator) included in each analysis. If relevant, these numbers should be by randomised group	Fig 1, 17
Outcomes and estimation:			
17a	For each primary and secondary outcome, results for each group, and the estimated effect size and its precision (such as 95% confidence interval)	For each objective, results including expressions of uncertainty (such as 95% confidence interval) for any estimates. If relevant, these results should be by randomised group	Table 3,4
17b	For binary outcomes, presentation of both absolute and relative effect sizes is recommended	Not applicable	
Ancillary analyses:			
18	Results of any other analyses performed, including subgroup analyses and adjusted analyses, distinguishing prespecified from exploratory	Results of any other analyses performed that could be used to inform the future definitive trial	17
Harms:			
19	All important harms or unintended effects in each group (for specific guidance see CONSORT for harms)		
19a		If relevant, other important unintended consequences	13
**Discussion**			
Limitations:			
20	Trial limitations, addressing sources of potential bias, imprecision, and, if relevant, multiplicity of analyses	Pilot trial limitations, addressing sources of potential bias and remaining uncertainty about feasibility	19-22
Generalisability:			
21	Generalisability (external validity, applicability) of the trial findings	Generalisability (applicability) of pilot trial methods and findings to future definitive trial and other studies	19-22
Interpretation:			
22	Interpretation consistent with results, balancing benefits and harms, and considering other relevant evidence	Interpretation consistent with pilot trial objectives and findings, balancing potential benefits and harms, and considering other relevant evidence	19-22
22a		Implications for progression from pilot to future definitive trial, including any proposed amendments	
**Other information**			

Registration:			
23	Registration number and name of trial registry	Registration number for pilot trial and name of trial registry	4, 23
Protocol:			
24	Where the full trial protocol can be accessed, if available	Where the pilot trial protocol can be accessed, if available	Ref 17
Funding:			
25	Sources of funding and other support (such as supply of drugs), role of funders		
26		Ethical approval or approval by research review committee, confirmed with reference number	23

*Here a pilot trial means any randomised study conducted in preparation for a future definitive RCT, where the main objective of the pilot trial is to assess feasibility.
